# Selective Functionalization with Organophosphite Ligands of Atomically Precise Platinum Chini Clusters

**DOI:** 10.1021/acs.inorgchem.6c01632

**Published:** 2026-05-20

**Authors:** Francesca Forti, Cristiana Cesari, Marco Bortoluzzi, Cristina Femoni, Maria Carmela Iapalucci, Stefano Zacchini

**Affiliations:** † Dipartimento di Chimica Industriale “Toso Montanari”, 9296 Università di Bologna, Via P. Gobetti 85, Bologna 40129 Italy; ‡ Dipartimento di Scienze Molecolari e Nanosistemi, 19047 Ca’ Foscari University of Venice, Via Torino 155, Mestre(Ve) 30175, Italy

## Abstract

Heteroleptic [Pt_3n_(CO)_6n–x_{P­(OR)_3_}_*x*_]^2–^ (n = 3–5; x = 1, 2; R = Me, Et, Ph) Chini clusters have been obtained upon reaction of homoleptic [Pt_3n_(CO)_6n_]^2–^ (n = 3–5) species with increasing amounts of P­(OR)_3_. In the case of P­(OPh)_3_, the whole series of clusters [Pt_3n_(CO)_6n–x_{P­(OPh)_3_}_*x*_]^2–^ (n = 3–5; x = 1, 2) has been spectroscopically characterized. In contrast, by using the stronger σ-bases P­(OMe)_3_ and P­(OEt)_3_, it has been possible to identify only the species [Pt_12_(CO)_22_{P­(OR)_3_}_2_]^2–^, [Pt_9_(CO)_17_{P­(OR)_3_}]^2–^ and [Pt_9_(CO)_16_{P­(OR)_3_}_2_]^2–^ (R = Me, Et). Generally speaking, 1–2 CO ligands may be selectively replaced by P­(OR)_3_ ligands in homoleptic [Pt_3n_(CO)_6n_]^2–^ (n = 3–5) clusters, whereas the addition of a third P­(OR)_3_ ligand results in the elimination of a Pt_3_-triangle and the concomitant formation of a smaller [Pt_3(n–1)_(CO)_6(n–1)_]^2–^ cluster that may be further substituted. The nature in solution of all the species has been elucidated by means of FT-IR, ESI-MS, ^1^H and ^31^P­{^1^H} NMR spectroscopy. The molecular structures of [PMePh_3_]_2_[Pt_9_(CO)_17_{P­(OPh)_3_}]·CH_3_COCH_3_, [PMePh_3_]_2_[Pt_12_(CO)_22_{P­(OPh)_3_}_2_]·solv, [PMePh_3_]_2_[Pt_12_(CO)_22_{P­(OMe)_3_}_2_], [PMePh_3_]_2_[Pt_15_(CO)_28_{P­(OPh)_3_}_2_]·2CH_3_COCH_3_·C_6_H_14_ have been determined by single-crystal X-ray diffraction (SC-XRD). Computational studies have been carried out to get insights into the torsional isomers of [Pt_12_(CO)_22_{P­(OR)_3_}_2_]^2–^ (R = Me, Ph) and the positional isomers of [Pt_9_(CO)_18–x_{P­(OMe)_3_}_*x*_]^2–^ (x = 1–3) and related species.

## Graphical abstract

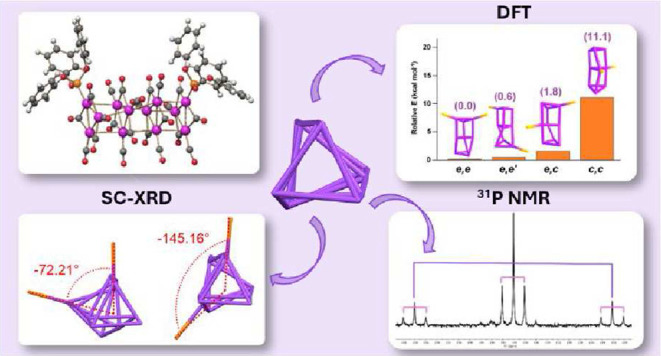

## 1 Introduction

Molecular homometallic homoleptic carbonyl clusters of general formula [Pt_3n_(CO)_6n_]^2–^ (n = 1–8), widely known as Chini clusters, represented a breakthrough in inorganic and cluster chemistry.^1–4^ They consist of stackings of “n” [Pt_3_(μ-CO)_3_(CO)_3_] units along a common *pseudo*-*C*_3_ axis in a slightly twisted trigonal prismatic fashion.^5–8^ They contain shorter intratriangular Pt–Pt bonds [2.65–2.68 Å] and longer intertriangular ones [3.02–3.24 Å]. All these clusters carry a −2 charge irrespective of the nuclearity and, thus, the formal oxidation state of each Pt atom is negative with an absolute value which decreases as the nuclearity increases. For instance, the formal oxidation state of Pt is −0.667 in [Pt_3_(CO)_6_]^2–^ (n = 1), −0.333 in [Pt_6_(CO)_12_]^2–^ (n = 2), −0.222 in [Pt_9_(CO)_18_]^2–^ (n = 3), −0.167 in [Pt_12_(CO)_24_]^2–^ (n = 4), −0.133 in [Pt_15_(CO)_30_]^2–^ (n = 5), and so on. This point has chemical, spectroscopic and structural consequences.

From a chemical point of view, oxidation of a [Pt_3n_(CO)_6n_]^2–^ Chini cluster results in a larger [Pt_3(n+1)_(CO)_6(n+1)_]^2–^ species, whereas its reduction leads to [Pt_3(n–1)_(CO)_6(n–1)_]^2–^.^1,4,9–11^ Therefore, the nuclearity of Chini clusters may be reversibly increased/decreased by a Pt_3_-unit upon chemical oxidation/reduction. This represents a unique case where the size of a molecular cluster may be varied in a modular fashion just using simple redox reactions.

The decrease of the charge to size ratio upon oxidation of [Pt_3n_(CO)_6n_]^2–^ to [Pt_3(n+1)_(CO)_6(n+1)_]^2–^ reduces the π-back-donation from the Pt cluster to CO ligands, whereas π-back-donation is favored upon reduction of Chini clusters.^1,4,5,12^ Thus, their ν_CO_ stretching bands move toward higher wavenumbers following oxidation, and toward lower wavenumbers upon reduction. FT-IR spectroscopy is, therefore, a very valuable and direct technique to determine the nuclearity of Chini clusters. Moreover, the HOMO–LUMO gap increases upon decreasing cluster size, resulting in blue-shifted absorption bands in the UV–vis spectra.^13^ Chini clusters exhibit vivid colors, corresponding to the complementary of absorbed ones, that vary with cluster size. This provides a visual indicator of [Pt_3n_(CO)_6n_]^2^^–^ cluster nuclearity: olive green (n = 6), yellow-green (n = 5), blue-green (n = 4), violet-red (n = 3), orange-red (n = 2), and pink-red (n = 1).^1^

As the nuclearity increases, the negative charge is spread over larger clusters reducing the electrostatic repulsion between anions. Consequently, Chini clusters with n ≥ 5 often self-assemble in the solid state, affording infinite conductive molecular Pt-wires, that may display low electrical resistivities.^14–16^ This makes larger Chini clusters attractive as molecular semiconductive or conductive materials.^17,18^ Chini clusters have found further applications as catalysts,^19–21^ precursors of catalysts and electrocatalysts,^22,23^ fluorescent quantum dots,^24^ substrates to obtain metal–organic frameworks and alloy nanoclusters,^25–28^ precursors of metal nanoparticles and nanowires.^29–34^ Their controlled thermolysis leads to globular molecular Pt nanoclusters, such as [Pt_19_(CO)_22_]^4–^, [Pt_23_(CO)_27_]^2–^, [Pt_24_(CO)_30_]^2–^, [Pt_26_(CO)_32_]^2–^, [Pt_27_(CO)_31_]^4–^, [Pt_33_(CO)_38_]^2–^, [Pt_36_(CO)_44_]^2–^, [Pt_38_(CO)_44_]^2–^, and [Pt_40_(CO)_40_]^6–^.^35–37^

Based on this rich literature on Chini clusters, they have historically contributed and still contribute to the very actual field of atomically precise Pt nanoclusters from two point of views.^38^ First of all, as molecular entities, they are perfectly defined nanoclusters which may selectively grow in one dimension up to the formation of infinite 1-D molecular Pt wires.^4,6,14,16^ Moreover, they may be used as precursors for the synthesis of homometallic and heterometallic molecular nanoclusters and ultrasmall nanoclusters.^18,25–37^

Broadly speaking, molecular Pt nanoclusters stabilized by miscellaneous ligands, sometimes also referred to as atomically precise Pt nanoclusters, have recently attracted great interest in nanochemistry, particularly because of their potential applications in catalysis and electrocatalysis.^38–48^ Atomically precise nanoclusters somehow fill the gaps among conventional coordination, cluster, colloidal, and catalytic chemistry.^49–62^ Key issues are the possibility of precisely controlling the number of Pt atoms and the overall shape of the metal core, as well as tailoring the surface properties by using various types of ligands. The choice of suitable ligands is indeed of fundamental importance in order to tune the structural, physical and chemical properties of ligand protected molecular nanoclusters in view of their potential applications.^52^

Within this framework, the chemistry of Chini clusters may be further expanded upon selective replacement of a few CO ligands with phosphines.^4^ A competition between the nonredox substitution with retention of the nuclearity and the redox fragmentation of the cluster has been observed while studying the reactivity of [Pt_3n_(CO)_6n_]^2–^ (n = 2–5) with monodentate and bidentate phosphines. The former process leads to heteroleptic Chini clusters with general formulas [Pt_3n_(CO)_6n–x_(PR_3_)_*x*_]^2–^ (n = 2–5; x = 1–5; PR_3_ = PPh_3_, PTA; PTA = 1,3,5-triaza-7-phosphaadamantane) and [Pt_3n_(CO)_6n–2x_(P–P)_*x*_]^2–^ (n = 2–6; x = 1–3; P–P = Ph_2_PCH_2_PPh_2_, Ph_2_PCH_2_CH_2_PPh_2_, *R*-Ph_2_PCH­(CH_3_)­CH_2_PPh_2_), whereas redox fragmentation results in lower nuclearity homoleptic [Pt_3(n–1)_(CO)_6(n–1)_]^2–^ species with concomitant elimination of Pt(0)-CO-PR_3_ species.^63–67^ Functionalization of Chini clusters may be exploited for their applications in catalysis, biology, medicinal chemistry, as chiral compounds and material precursors.

Herein, we expand the scope of Chini clusters functionalization using monodentate P­(OR)_3_ (R = Me, Et, Ph) organophosphite ligands, which show a greater π-acidity compared to phosphines. This allowed for the first time the direct functionalization of larger Chini clusters such as [Pt_15_(CO)_30_]^2–^ leading to [Pt_15_(CO)_28_{P­(OPh)_3_}_2_]^2–^, and the structural characterization by single-crystal X-ray diffraction (SC-XRD) of a monosubstituted Chini cluster, that is, [Pt_9_(CO)_17_{P­(OPh)_3_}]^2–^. The results herein reported clearly point out that carefully tuning ligand properties, it is possible to perform the controlled functionalization of lower to higher nuclearity Chini clusters.

## 2 Results and Discussion

### 2.1 Reactions of [Pt_3n_(CO)_6n_]^2–^ (n = 3–5) with P­(OPh)_3_: Synthesis and Spectroscopic Characterization of [Pt_3n_(CO)_6n–x_{P­(OPh)_3_}_*x*_]^2–^ (n = 3–5; x = 1, 2)

The outcome of all the reactions described in this paper does not depend on the nature of the cation employed, that is, [PMePh_3_]^+^, [NEt_4_]^+^, or [NBu_4_]^+^. Thus, only the formulas of the cluster anions will be indicated in general, except in the case of crystals, for which both cations and anions will be indicated.

The reaction of [Pt_15_(CO)_30_]^2–^ with one mole equivalent of P­(OPh)_3_ affords [Pt_15_(CO)_29_{P­(OPh)_3_}]^2–^ by substitution of one CO ligand (Scheme 1). A second carbonyl may be replaced upon addition of a further mole equivalent of P­(OPh)_3_, resulting in the formation of [Pt_15_(CO)_28_{P­(OPh)_3_}_2_]^2–^. The addition of a third mole equivalent of P­(OPh)_3_ results in the formal elimination of a Pt_3_ unit and the formation of a mixture of [Pt_12_(CO)_24_]^2–^, [Pt_12_(CO)_23_{P­(OPh)_3_}]^2–^ and [Pt_12_(CO)_22_{P­(OPh)_3_}_2_]^2–^, as evidenced by FT-IR and ^31^P­{^1^H} NMR spectroscopy. Pt atoms are likely eliminated as neutral Pt(0) complexes.^63–67^ Indeed, a few crystals of [Pt­{P­(OPh)_3_}_3_]·THF suitable for SC-XRD analyses have been isolated during the work up of the reaction mixture, together with crystals of [PMePh_3_]_2_[Pt_12_(CO)_24_].

**Scheme 1:**
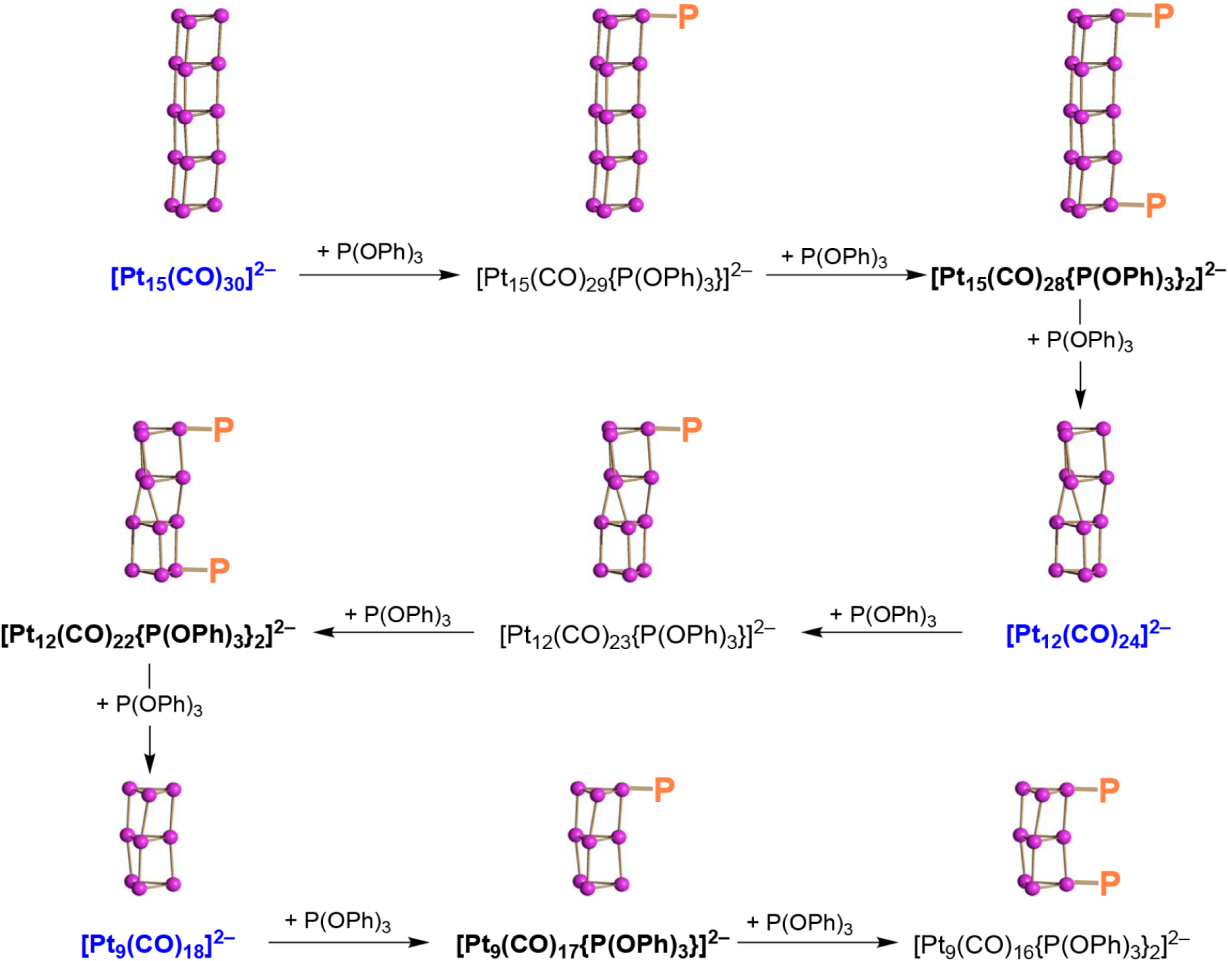
Reactions of [Pt_3n_(CO)_6n_]^2–^ (n = 3–5) with Increasing Amounts of P­(OPh)_3_ ^a^

a Homoleptic Chini clusters are reported in blue. The structures of the compounds reported in bold have been determined by SC-XRD. Only Pt and P atoms have been included in the scheme for clarity.

Thus, to obtain purer [Pt_12_(CO)_23_{P­(OPh)_3_}]^2–^ and [Pt_12_(CO)_22_{P­(OPh)_3_}_2_]^2–^, it is better to add one or two, respectively, mole equivalents of P­(OPh)_3_ to [Pt_12_(CO)_24_]^2–^. A further addition of P­(OPh)_3_ results in the elimination of one Pt_3_ unit affording mixtures of [Pt_9_(CO)_18_]^2–^, [Pt_9_(CO)_17_{P­(OPh)_3_}]^2–^ and [Pt_9_(CO)_16_{P­(OPh)_3_}_2_]^2–^, sometimes together with some unreacted [Pt_12_(CO)_22_{P­(OPh)_3_}_2_]^2–^. Also in this case, the best method to obtain [Pt_9_(CO)_17_{P­(OPh)_3_}]^2–^ and [Pt_9_(CO)_16_{P­(OPh)_3_}_2_]^2–^ consists in the addition of one or two, respectively, mole equivalents of P­(OPh)_3_ to [Pt_9_(CO)_18_]^2–^.

All the new heteroleptic Chini clusters [Pt_3n_(CO)_6n–x_{P­(OPh)_3_}_*x*_]^2–^ (n = 3–5; x = 1, 2) have been characterized by FT-IR, ^1^H and ^31^P­{^1^H} NMR spectroscopy (Figures S1–S47 in the Supporting Information). Moreover, the molecular structures of [PMePh_3_]_2_[Pt_9_(CO)_17_{P­(OPh)_3_}]·CH_3_COCH_3_, [PMePh_3_]_2_[Pt_12_(CO)_22_{P­(OPh)_3_}_2_] and [PMePh_3_]_2_[Pt_15_(CO)_28_{P­(OPh)_3_}_2_]·2CH_3_COCH_3_·C_6_H_14_ have been determined by SC-XRD.

FT-IR spectra recorded in acetone solution (Figure 1 and Table 1) indicate that the ν_CO_ bands are moved toward lower wavenumbers by 4–10 cm^–1^ upon each substitution of a CO ligand with one P­(OPh)_3_ ligand, as previously found for phosphine ligands.^63–67^ Due to the reduced basicity of P­(OPh)_3_ compared to PPh_3_, it has been possible to isolate the Pt_15_-derivatives [Pt_15_(CO)_29_{P­(OPh)_3_}]^2–^ and [Pt_15_(CO)_28_{P­(OPh)_3_}_2_]^2–^. In contrast, the reaction of [Pt_15_(CO)_30_]^2–^ with PPh_3_ produced [Pt_12_(CO)_24_]^2–^ and its derivatives [Pt_12_(CO)_23_(PPh_3_)]^2–^ and [Pt_12_(CO)_22_(PPh_3_)_2_]^2–^.^67^ It is noteworthy that [Pt_15_(CO)_28_{P­(OPh)_3_}_2_]^2–^ displays ν_CO_ bands at lower wavenumbers than [Pt_12_(CO)_24_]^2–^ and very close to those of [Pt_12_(CO)_23_{P­(OPh)_3_}]^2–^. Since all these Pt_15_ and Pt_12_ derivatives are green in solution, their identification based solely on FT-IR spectroscopy is not trivial. Further information may be obtained using ^31^P­{^1^H} NMR spectroscopy as described below, and the structure of [Pt_15_(CO)_28_{P­(OPh)_3_}_2_]^2–^ has been unequivocally determined by SC-XRD.

**Figure 1:**
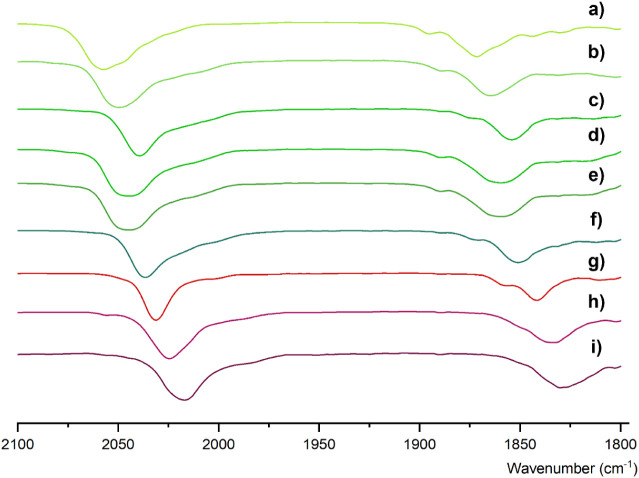
FT-IR spectra in the ν_CO_ region, recorded at room temperature in acetone solution, of compounds: (**a**) [Pt_15_(CO)_30_]^2–^, (**b**) [Pt_15_(CO)_29_{P­(OPh)_3_}]^2–^, (**c**) [Pt_15_(CO)_28_{P­(OPh)_3_}_2_]^2–^, (**d**) [Pt_12_(CO)_24_]^2–^, (**e**) [Pt_12_(CO)_23_{P­(OPh)_3_}]^2–^, (**f**) [Pt_12_(CO)_22_{P­(OPh)_3_}_2_]^2–^, (**g**) [Pt_9_(CO)_18_]^2–^, (**h**) [Pt_9_(CO)_17_{P­(OPh)_3_}]^2–^, (**i**) [Pt_9_(CO)_16_{P­(OPh)_3_}_2_]^2–^. Clusters (**b**–**f**) are synthesized from the stepwise addition of P­(OPh)_3_ to [Pt_15_(CO)_30_]^2–^, while (**h**–**i**) from the stepwise addition of P­(OPh)_3_ to [Pt_9_(CO)_18_]^2–^.

**Table 1:** ν_CO_ Bands of Homoleptic Chini Clusters and Their Heteroleptic Derivatives Containing Organophosphite or Phosphine Ligands

Compound	P(OPh)_3_ ^a^	P(OMe)_3_ ^a^	P(OEt)_3_ ^a^	PPh_3_ ^b^	PTA^b^
[Pt_15_(CO)_30_]^2–^	2056(s), 1872(m)				
[Pt_15_(CO)_29_(L)]^2–^	2050(s), 1864(m)	-	-	-	
[Pt_15_(CO)_28_(L)_2_]^2–^	2040(s), 1855(m)	-	-	-	
[Pt_15_(CO)_25_(L)_5_]^2–^	-	-	-	-	2015(s), 1834(m)
[Pt_12_(CO)_24_]^2–^	2046(s), 1859(m)				
[Pt_12_(CO)_23_(L)]^2–^	2042(s), 1859(m)	-	-	2042(s), 1854(m)	2040(s), 1854(m)
[Pt_12_(CO)_22_(L)_2_]^2–^	2037(s), 1854(m)	2038(s), 1853(m)	2039(s), 1854(m)	2036(s), 1848(m)	2035(s), 1844(m)
[Pt_12_(CO)_20_(L)_4_]^2–^	-	-	-	-	2014(s), 1830(m)
[Pt_9_(CO)_18_]^2–^	2032(s), 1841(m)				
[Pt_9_(CO)_17_(L)]^2–^	2023(s), 1833(m)	2025(s), 1834(m)	2024(s), 1835(m)	2024(s), 1830(m)	2025(s), 1831(m)
[Pt_9_(CO)_16_(L)_2_]^2–^	2017(s), 1831(m)	2016(s), 1826(m)	2018(s), 1830(m)	2018(s), 1820(m)	2014(s), 1826(m)
[Pt_6_(CO)_12_]^2–^	2001(s), 1802(m)				
[Pt_6_(CO)_11_(L)]^2–^	-	-	-		1992(s), 1779(m)
[Pt_6_(CO)_10_(L)_2_]^2–^	-	1979(s), 1775(m)	-	1973(s), 1756(m)	1976(s), 1759(m)

a In acetone.

b In CH_3_CN. Data taken from ref 67 (PPh_3_) and ref 64 (PTA).

All the species [Pt_3n_(CO)_6n–x_{P­(OPh)_3_}_*x*_]^2–^ (n = 3–5; x = 1, 2) show a very similar pattern of the ^31^P­{^1^H} NMR spectra, consisting of a single complex multiplet (Figure 2 and Table 2). This is in keeping with the presence of a single P­(OPh)_3_ ligand in monosubstituted species [Pt_3n_(CO)_6n–1_{P­(OPh)_3_}]^2–^ (n = 3–5). In the case of disubstituted clusters [Pt_3n_(CO)_6n–2_{P­(OPh)_3_}_2_]^2–^ (n = 3–5), the presence of a single ^31^P­{^1^H} NMR resonance indicates that the two ligands are equivalent, in keeping with the fact that both P­(OPh)_3_ ligands are bonded to the two external Pt_3_ unit, as found in the solid state structures. The complex pattern of the multiplet arises from the isotopic distribution of Pt, and the strong coupling of P with the directly bonded Pt atom (^1^J_Pt–P_ = 8706–8810 Hz) and a weaker coupling with the other two equivalent Pt-atoms within the same Pt_3_ triangle (^2^J_Pt–P_ = 983–1004 Hz). No coupling to Pt atoms of the internal unsubstituted Pt_3_ triangles is observed, as previously found in related heteroleptic Chini clusters^63,64,67^ and in the ^13^C NMR spectra of the homoleptic [Pt_3n_(CO)_6n_]^2–^ (n = 2–6) species.^68^ Since the coupling is limited to a single Pt_3_ unit, the observed coupling pattern does not depend on the cluster size and is the same for mono- and (symmetrically) disubstituted clusters.

**Figure 2:**
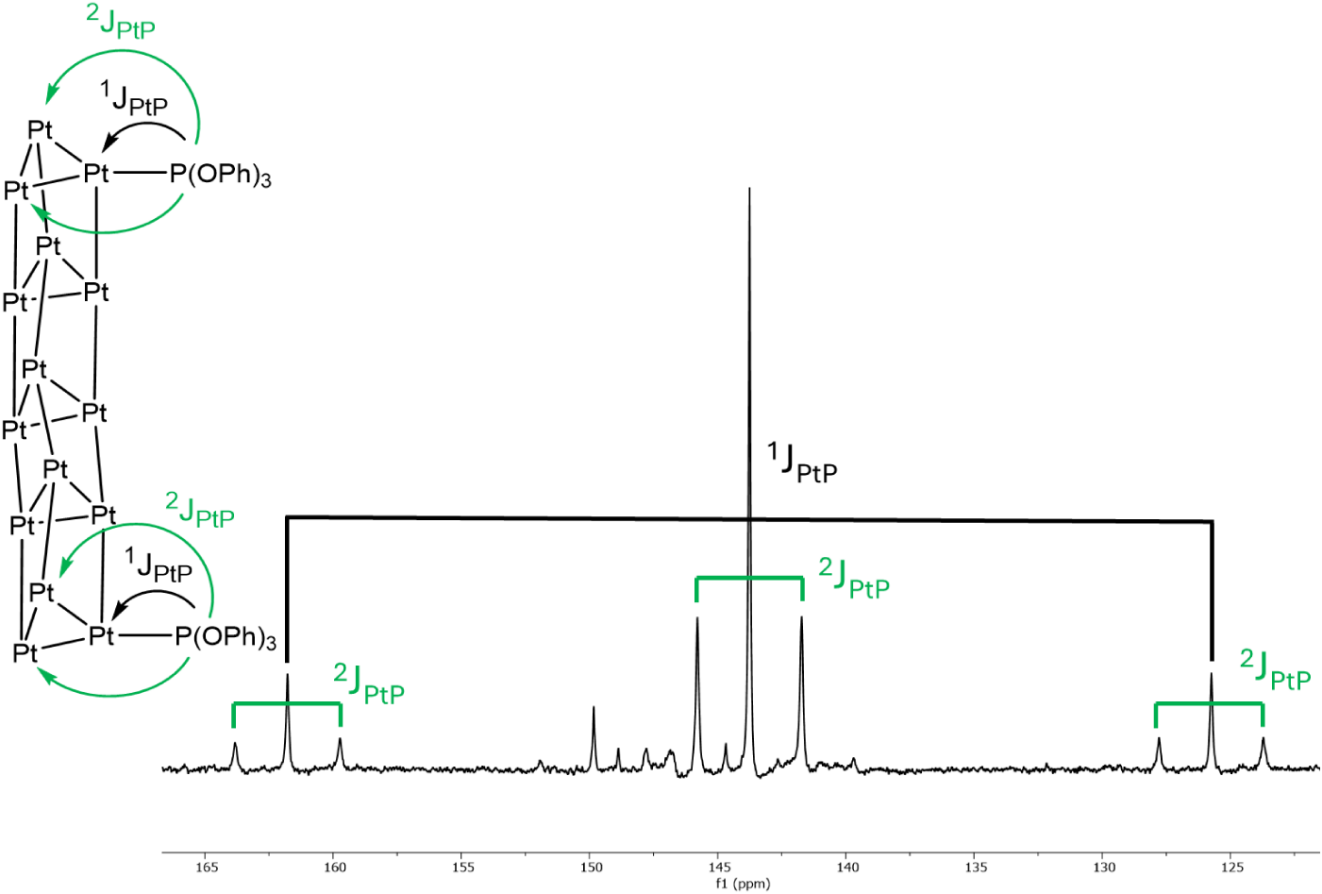
^31^P­{^1^H} NMR spectrum of [PMePh_3_]_2_[Pt_15_(CO)_28_{P­(OPh)_3_}_2_] in acetone-d_6_. The two P atoms are equivalent. Signals not indicated by brackets are due to unidentified impurities.

**Table 2:** ^31^P­{^1^H} NMR Data of [Pt_3n_(CO)_6n–x_{P­(OPh)_3_}*_x_*]^2–^ (n = 3–5; x = 1, 2) Recorded at Room Temperature in Deuterated Acetone

Compound	δ_P_ (ppm)	^1^J_Pt**–**P_ (Hz)	^2^J_Pt**–**P_ (Hz)
P(OPh)_3_	128.0	-	-
[Pt_15_(CO)_29_{P(OPh)_3_}]^2–^	140.1	8706	983
[Pt_15_(CO)_28_{P(OPh)_3_}_2_]^2–^	143.8	8755	987
[Pt_12_(CO)_23_{P(OPh)_3_}]^2–^	144.7	8768	987
[Pt_12_(CO)_22_{P(OPh)_3_}_2_]^2–^	148.1	8778	992
[Pt_9_(CO)_17_{P(OPh)_3_}]^2–^	148.6	8810	992
[Pt_9_(CO)_16_{P(OPh)_3_}_2_]^2–^	149.6	8731	1004

It is noteworthy that the ^31^P chemical shift of the [Pt_3n_(CO)­6n_–x_{P­(OPh)_3_}_*x*_]^2–^ (n = 3–5; x = 1, 2) clusters systematically moves toward higher frequencies as the nuclearity of the cluster decreases and the number of P­(OPh)_3_ ligands increases. A similar trend was previously observed for related PPh_3_-derivatives of Chini clusters.^67^

### 2.2 Reactions of [Pt_3n_(CO)_6n_]^2–^ (n = 3–5) with P­(OMe)_3_ and P­(OEt)_3_: Synthesis and Spectroscopic Characterization of [Pt_3n_(CO)_6n–x_{P­(OR)_3_}_*x*_]^2–^ (n = 3–4; x = 1, 2; R = Me, Et)

Some interesting differences may be noticed using P­(OMe)_3_ and P­(OEt)_3_, that are stronger σ-bases compared to P­(OPh)_3_. Indeed, their behavior resembles that previously observed for PPh_3_.^67^ Thus, addition of P­(OMe)_3_ and P­(OEt)_3_ to [Pt_15_(CO)_30_]^2–^ results in its reduction to [Pt_12_(CO)_24_]^2–^ via the elimination of a Pt_3_ triangle (Scheme 2). Even by using substoichiometric amounts of P­(OR)_3_, mixtures of [Pt_12_(CO)_24_]^2–^ and unreacted [Pt_15_(CO)_30_]^2–^ have been observed rather than substituted Pt_15_ clusters. Also, the species [Pt_12_(CO)_23_{P­(OR)_3_}]^2–^ seems to be very elusive and only the disubstituted clusters [Pt_12_(CO)_22_{P­(OR)_3_}_2_]^2–^ (R = Me, Et) have been isolated while studying the reaction of [Pt_12_(CO)_24_]^2–^ with P­(OMe)_3_ and P­(OEt)_3_. In contrast, starting from the more reduced [Pt_9_(CO)_18_]^2–^, it has been possible to spectroscopically characterize both [Pt_9_(CO)_17_{P­(OR)_3_}]^2–^ and [Pt_9_(CO)_16_{P­(OR)_3_}_2_]^2–^.

**Scheme 2:**
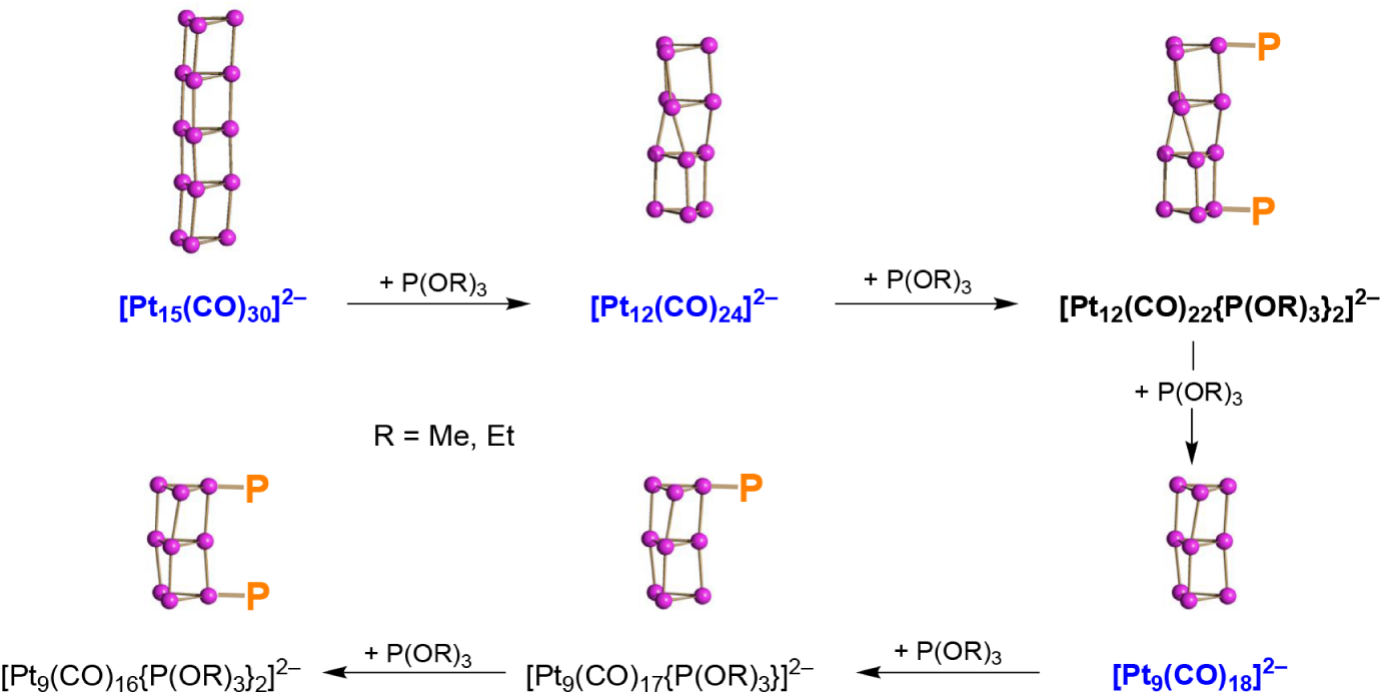
Reactions of [Pt_3n_(CO)_6n_]^2–^ (n = 3–5) with Increasing Amounts of P­(OR)_3_ (R = Me, Et)[Fn sch2-fn2]

a Homoleptic Chini clusters are reported in blue. The structures of the compounds reported in bold have been determined by SC-XRD. Only Pt and P atoms have been included in the scheme for clarity.

All the new heteroleptic Chini clusters [Pt_12_(CO)_22_{P­(OR)_3_}_2_]^2–^, [Pt_9_(CO)_17_{P­(OR)_3_}]^2–^ and [Pt_9_(CO)_16_{P­(OR)_3_}_2_]^2–^ (R = Me, Et) have been characterized by FT-IR, ^1^H and ^31^P­{^1^H} NMR spectroscopy (Figures S1–S47 in the Supporting Information). Moreover, the molecular structure of [MePPh_3_]_2_[Pt_12_(CO)_22_{P­(OMe)_3_}_2_] has been determined by SC-XRD. The spectroscopic FT-IR and NMR spectra display patterns and trends very similar to those previously commented on the related P­(OPh)_3_ derivatives (Figures 3–6 and Tables 3–4).

**Figure 3:**
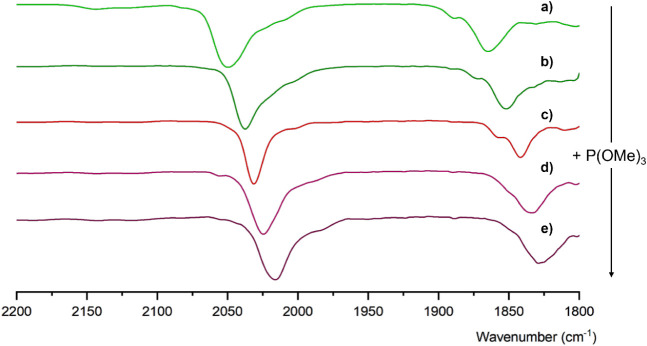
FT-IR spectra in the ν_CO_ region, recorded at room temperature in acetone solution, obtained upon the stepwise addition of P­(OMe)_3_ to [Pt_12_(CO)_24_]^2–^. (**a**) [Pt_12_(CO)_24_]^2–^, (**b**) [Pt_12_(CO)_22_{P­(OMe)_3_}_2_]^2–^, (**c**) [Pt_9_(CO)_18_]^2–^, (**d**) [Pt_9_(CO)_17_{P­(OMe)_3_}]^2–^, (**e**) [Pt_9_(CO)_16_{P­(OMe)_3_}_2_]^2–^.

**Figure 4:**
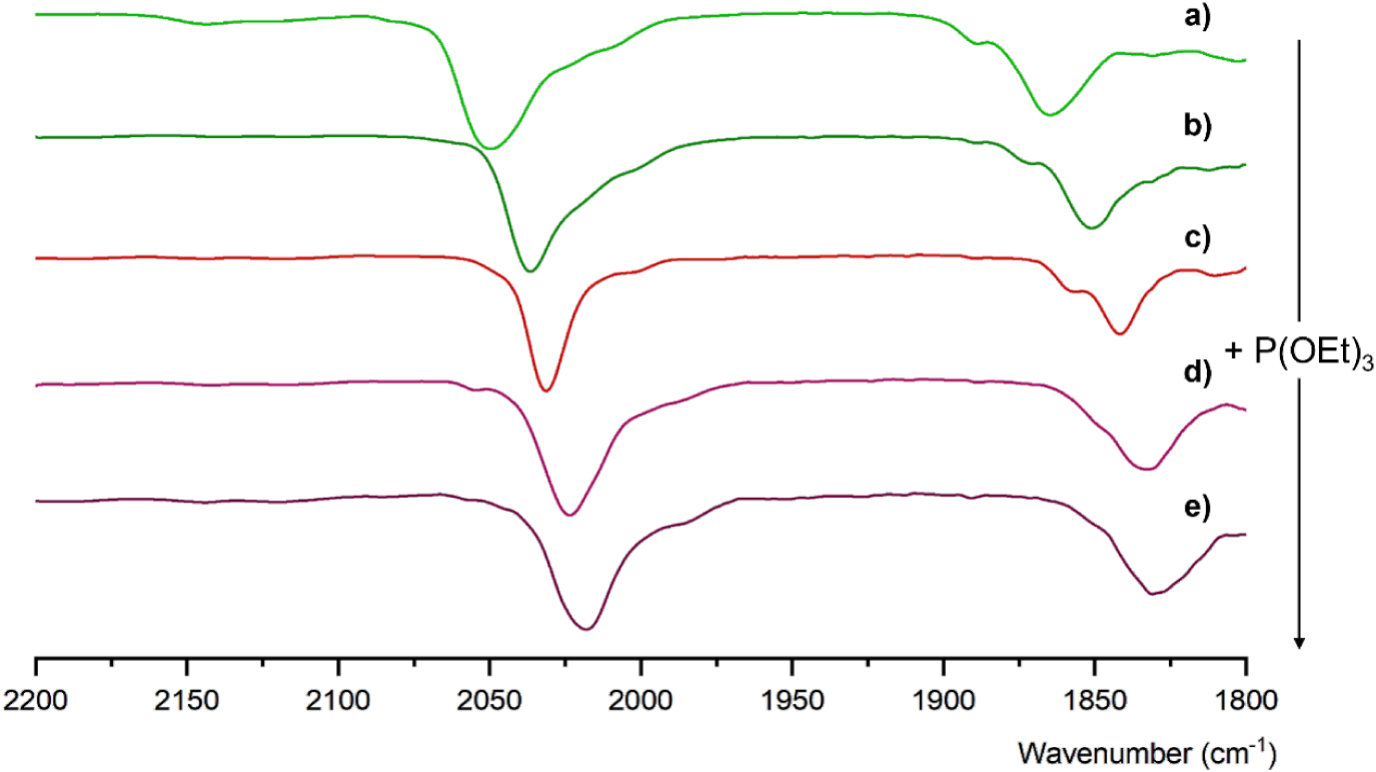
FT-IR spectra in the ν_CO_ region, recorded at room temperature in acetone solution, obtained upon the stepwise addition of P­(OEt)_3_ to [Pt_12_(CO)_24_]^2–^. (**a**) [Pt_12_(CO)_24_]^2–^, (**b**) [Pt_12_(CO)_22_{P­(OEt)_3_}_2_]^2–^, (**c**) [Pt_9_(CO)_18_]^2–^, (**d**) [Pt_9_(CO)_17_{P­(OEt)_3_}]^2–^, (**e**) [Pt_9_(CO)_16_{P­(OEt)_3_}_2_]^2–^.

**5:**
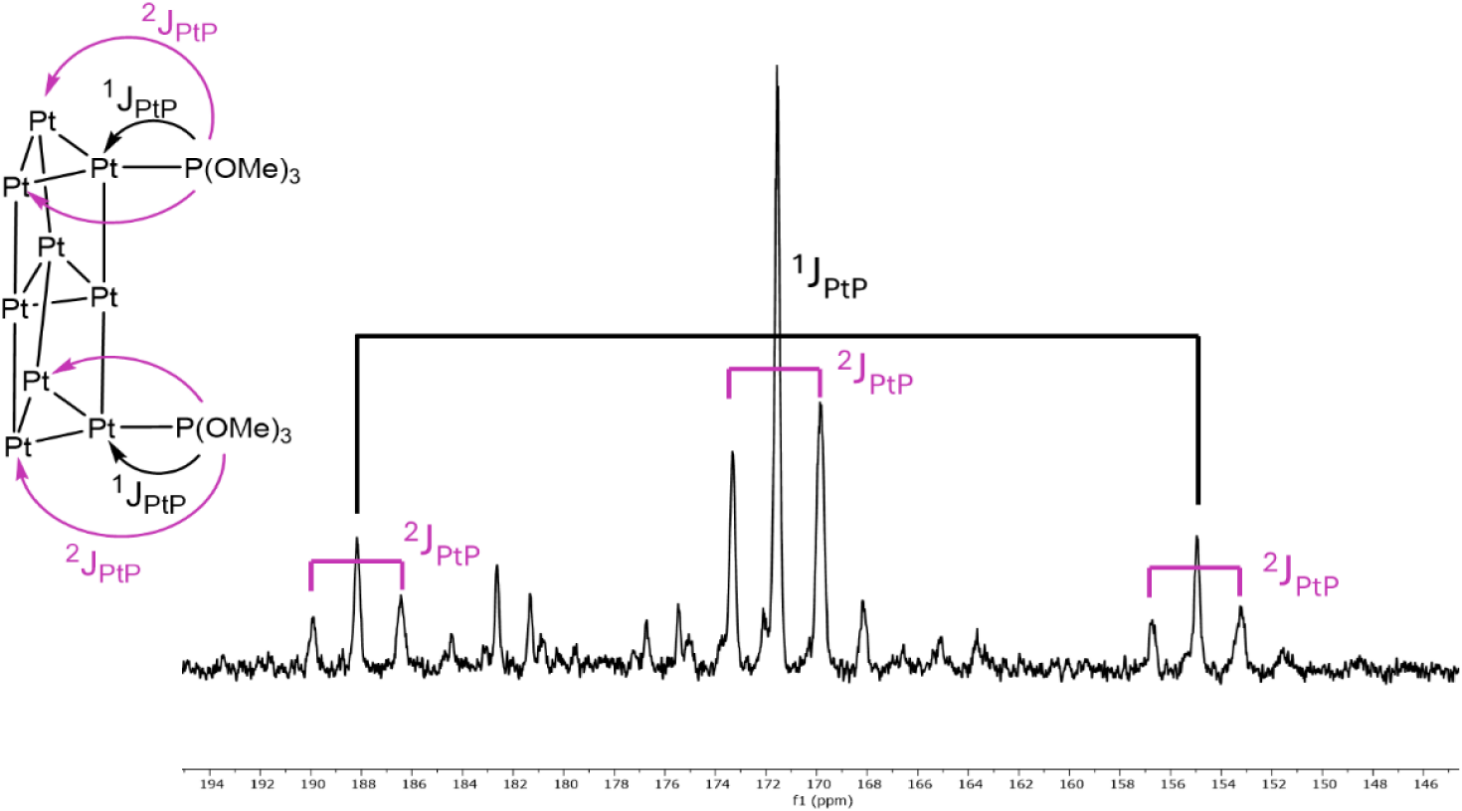
^31^P­{^1^H} NMR spectrum of [NEt_4_]_2_[Pt_9_(CO)_16_{P­(OMe)_3_}_2_] in acetone-d_6_. The two P atoms are equivalent. Signals not indicated by brackets are due to unidentified impurities.

**Figure 6:**
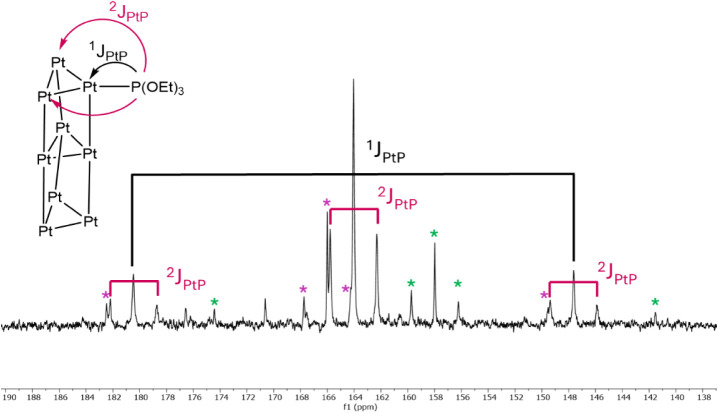
^31^P­{^1^H} NMR spectrum of [PMePh_3_]_2_[Pt_9_(CO)_16_{P­(OEt)_3_}] in acetone-d_6_ (signals indicated by brackets). Traces of compounds [PMePh_3_]_2_[Pt_12_(CO)_22_{P­(OEt)_3_}_2_] (green stars) and [PMePh_3_]_2_[Pt_9_(CO)_16_{P­(OMe)_3_}_2_] (purple stars). Signals not indicated by brackets are due to unidentified impurities.

**Table 3:** ^31^P­{^1^H} NMR Data of [Pt_3n_(CO)_6n–x_{P­(OMe)_3_}*_x_*]^2–^ (n = 3–4; x = 1, 2) Recorded at Room Temperature in Deuterated Acetone

Compound	δ_P_ (ppm)	^1^J_Pt**–**P_ (Hz)	^2^J_Pt**–**P_ (Hz)
P(OMe)_3_	140.5	-	-
[Pt_12_(CO)_22_{P(OMe)_3_}_2_]^2–^	163.8	8071	861
[Pt_9_(CO)_17_{P(OMe)_3_}]^2–^	169.8	8053	843
[Pt_9_(CO)_16_{P(OMe)_3_}_2_]^2–^	171.5	8068	842

**Table 4:** ^31^P­{^1^H} NMR Data of [Pt_3n_(CO)_6n–x_{P­(OEt)_3_}*_x_*]^2–^ (n = 3–4; x = 1, 2) Recorded at Room Temperature in Deuterated Acetone

Compound	δ_P_ (ppm)	^1^J_Pt**–**P_ (Hz)	^2^J_Pt**–**P_ (Hz)
P(OEt)_3_	138.4	-	-
[Pt_12_(CO)_22_{P(OEt)_3_}_2_]^2–^	158.0	7998	851
[Pt_9_(CO)_17_{P(OEt)_3_}]^2–^	164.0	7979	845
[Pt_9_(CO)_16_{P(OEt)_3_}_2_]^2–^	165.6	7998	845

The highly reduced [Pt_6_(CO)_12_]^2–^ cluster seems to be very reluctant to CO substitution with P­(OR)_3_ ligands (R = Me, Et, Ph). Indeed, using stoichiometric amounts of P­(OR)_3_ does not result in any apparent reaction. Only after the addition of a slight excess of P­(OMe)_3_ in MeCN solution, [Pt_6_(CO)_12_]^2–^ is partially converted into a new species displaying ν_CO_ bands at 1979 and 1775 cm^–1^. By comparison with analogous reactions with PPh_3_,^67^ such FT-IR data are more in agreement with a disubstituted [Pt_6_(CO)_10_{P­(OMe)_3_}_2_]^2–^ species than monosubstituted [Pt_6_(CO)_11_{P­(OMe)_3_}]^2–^. Because of limited stability, it has not been possible to further characterize this compound.

The [Pt_3n_(CO)_6n–x_{P­(OR)_3_}_*x*_]^2–^ (n = 3–5; x = 1, 2; R = Me, Et, Ph) clusters are stable under CO atmosphere. The higher nuclearity clusters (n = 4, 5) are reduced to mixtures of [Pt_9_(CO)_18_]^2–^, [Pt_9_(CO)_17_{P­(OR)_3_}]^2–^ and [Pt_9_(CO)_16_{P­(OR)_3_}_2_]^2–^ upon exposure to H_2_ atmosphere. The [Pt_3n_(CO)_6n–x_{P­(OR)_3_}_*x*_]^2–^ (n = 3–5; x = 1, 2; R = Me, Et, Ph) clusters are oxidized to higher nuclearity species by strong acids such as HBF_4_·Et_2_O, but this process often is accompanied by elimination of the P­(OR)_3_ ligand and formation of homoleptic Chini clusters.

### 2.3 Molecular Structures of [Pt_9_(CO)_17_{P­(OPh)_3_}]^2–^, [Pt_12_(CO)_22_{P­(OPh)_3_}_2_]^2–^, [Pt_12_(CO)_22_{P­(OMe)_3_}_2_]^2–^, and [Pt_15_(CO)_28_{P­(OPh)_3_}_2_]^2**–**^

The molecular structures of the heteroleptic anions [Pt_9_(CO)_17_{P­(OPh)_3_}]^2–^, [Pt_12_(CO)_22_{P­(OPh)_3_}_2_]^2–^, [Pt_12_(CO)_22_{P­(OMe)_3_}_2_]^2–^ and [Pt_15_(CO)_28_{P­(OPh)_3_}_2_]^2–^ have been determined by SC-XRD on their [PMePh_3_]_2_[Pt_9_(CO)_17_{P­(OPh)_3_}]·CH_3_COCH_3_, [PMePh_3_]_2_[Pt_12_(CO)_22_{P­(OPh)_3_}_2_]·solv, [PMePh_3_]_2_[Pt_12_(CO)_22_{P­(OMe)_3_}_2_], [PMePh_3_]_2_[Pt_15_(CO)_28_{P­(OPh)_3_}_2_]·2CH_3_COCH_3_·C_6_H_14_ salts (Figures S54–S59 in the Supporting Information for thermal ellipsoid plots).

[Pt_9_(CO)_17_{P­(OPh)_3_}]^2–^ represents the first structurally characterized monosubstituted heteroleptic Pt Chini cluster (Figure 7 and Table 5). It retains the prismatic structure of the parent [Pt_9_(CO)_18_]^2–^,^5,28,63^ where one terminal CO ligand of one external Pt_3_-triangle has been replaced by a P­(OPh)_3_ ligand. The average intra- [2.6609(14) Å] and intertriangular [3.0401(14) Å] Pt–Pt distances of [Pt_9_(CO)_17_{P­(OPh)_3_}]^2–^ are comparable to those of the parent [Pt_9_(CO)_18_]^2–^ ^5,28,63^ [2.66(9) and 3.05(4) Å, respectively] and the related disubstituted species [Pt_9_(CO)_16_(PPh_3_)_2_]^2–^ [2.670(2) and 3.043(2) Å, respectively].^67^ The intratriangular Pt–Pt distances of [Pt_9_(CO)_17_{P­(OPh)_3_}]^2–^ are comprised in a very narrow range [2.6537(5)–2.6647(5) Å] and shorter than the intertriangular ones [3.0238(6)–3.0544(6) Å], as systematically found in all homoleptic and heteroleptic Pt carbonyl Chini clusters.^4^ The intertriangular Pt–Pt bonding contacts of [Pt_9_(CO)_17_{P­(OPh)_3_}]^2–^ display a very narrow distribution, as found in the parent [Pt_9_(CO)_18_]^2–^ [3.04(2)–3.06(2) Å].^5^ Conversely, the intertriangular Pt–Pt bonds of [Pt_9_(CO)_16_(PPh_3_)_2_]^2–^ are more scattered [3.0015(9)–3.1098(8) Å].^67^ This suggests that monosubstitution does not significantly alter the Pt_9_ cage of the cluster, whereas disubstitution introduces some deformations of the intertriangular stacking. The Pt–P bond of [Pt_9_(CO)_17_{P­(OPh)_3_}]^2–^ [2.186(3) Å] is slightly shorter than in [Pt_9_(CO)_16_(PPh_3_)_2_]^2–^ [2.265(4) and 2.287(4) Å], due to the greater π-acid character of P­(OPh)_3_ compared to PPh_3_.

**Figure 7:**
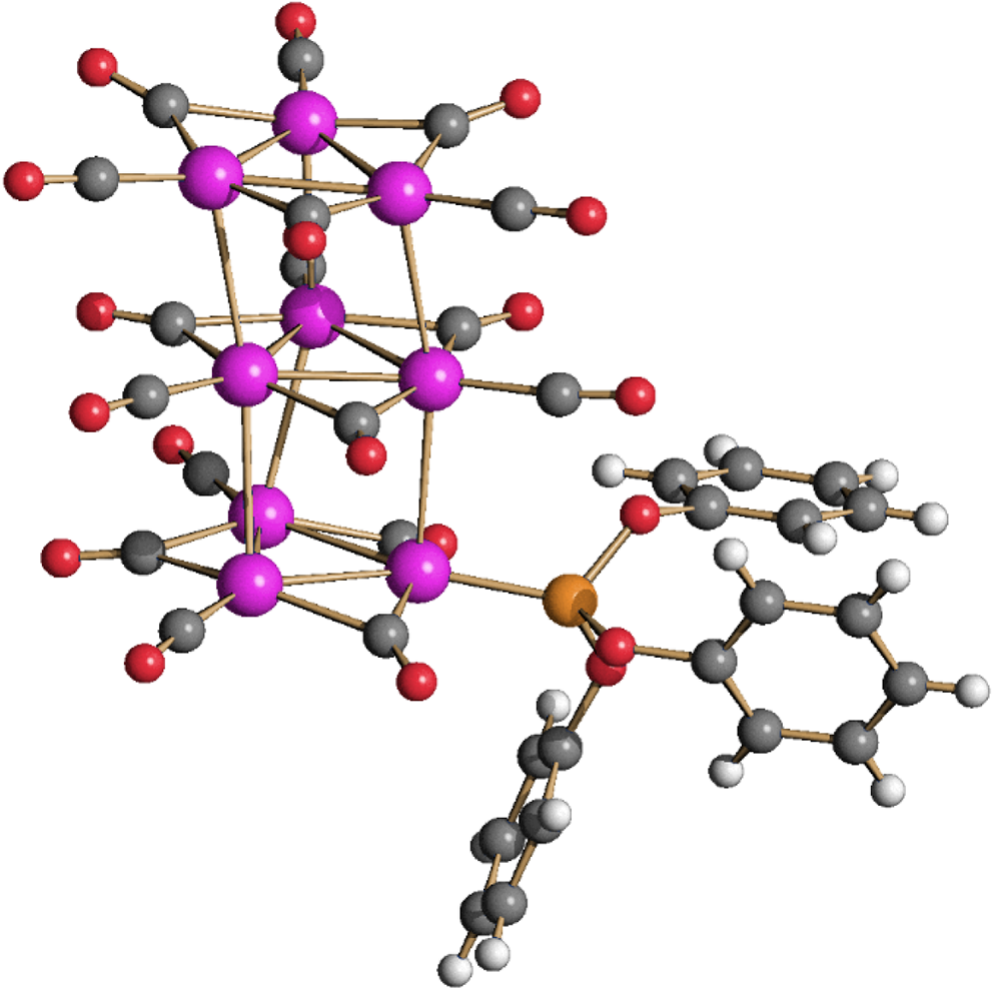
Molecular structure of [Pt_9_(CO)_17_{P­(OPh)_3_}]^2–^ (purple, Pt; orange, P; red, O; gray, C; white, H).

**Table 5:** Main Bond Distances (Å) of [Pt_15_(CO)_28_{P­(OPh)_3_}_2_]^2–^, [Pt_12_(CO)_22_{P­(OPh)_3_}_2_]^2–^, [Pt_12_(CO)_22_{P­(OMe)_3_}_2_]^2–^ and [Pt_9_(CO)_17_{P­(OPh)_3_}]^2–^ Compared to [Pt_15_(CO)_30_]^2–^, [Pt_12_(CO)_24_]^2–^, [Pt_12_(CO)_22_(PPh_3_)_2_]^2–^, [Pt_12_(CO)_22_(PPh_2_Py)_2_]^2–^, [Pt_9_(CO)_16_(PPh_3_)_2_]^2–^, and [Pt_9_(CO)_18_]^2–^

	Pt–Pt Intratriangular	Pt–Pt Intertriangular	Pt–P
[Pt_15_(CO)_30_]^2–^ ^a^	2.649(2)–2.6703(19)	2.9972(14)–3.0783(18)	-
	Average 2.658(6)	Average 3.036(4)	
[Pt_15_(CO)_28_{P(OPh)_3_}_2_]^2–^	2.6579(6)–2.6706(7)	3.0219(5)–3.1066(5)	2.190(3)
	Average 2.6654(18)	Average 3.0549(12)	
[Pt_12_(CO)_24_]^2–^ ^b^	2.6587(5)–2.6707(5)	3.0465(5)–3.0597(5)	-
	Average 2.6656(12)	Average 3.0535(12)	
[Pt_12_(CO)_22_{P(OPh)_3_}_2_]^2–^	2.6529(11)–2.6716(16)	3.0054(12)–3.0754(12)	2.189(5), 2.198(5)
	Average 2.663(4)	Average 3.050(3)	
[Pt_12_(CO)_22_{P(OMe)_3_}_2_]^2–^	2.6578(6)–2.6771(7)	3.0143(6)–3.0926(6)	2.204(3), 2.218(3)
	Average 2.667(2)	Average 3.0511(18)	
[Pt_12_(CO)_22_(PPh_3_)_2_]^2–^ ^c^	2.6539(9)–2.6756(9)	3.0184(9)–3.2067(11)	2.281(4)
	Average 2.666(2)	Average 3.086(2)	
[Pt_12_(CO)_22_(PPh_2_Py)_2_]^2–^ ^a^	2.6505(15)–2.6681(17)	2.9919(16)–3.173(2)	2.265(7)
	Average 2.660(4)	Average 3.054(4)	
[Pt_9_(CO)_18_]^2–^ ^b^	2.65(2)–2.67(8)	3.04(2)–3.06(2)	-
	Average 2.66(9)	Average 3.05(4)	
[Pt_9_(CO)_17_{P(OPh)_3_}]^2–^	2.6537(5)–2.6647(5)	3.0238(6)–3.0544(6)	2.186(3)
	Average 2.6609(14)	Average 3.0401(14)	
[Pt_9_(CO)_16_(PPh_3_)_2_]^2–^ ^c^	2.6597(8)–2.6814(9)	3.0015(9)–3.1098(8)	2.265(4), 2.287(4)
	Average 2.670(2)	Average 3.043(2)	

a From ref 63.

b From ref 5.

c From ref 67.

As previously found in [Pt_12_(CO)_22_(PPh_3_)_2_]^2–^ and [Pt_12_(CO)_22_(PPh_2_Py)_2_]^2–^,^63,67^ the two P­(OR)_3_ (R = Me, Ph) ligands of [Pt_12_(CO)_22_{P­(OPh)_3_}_2_]^2–^ and [Pt_12_(CO)_22_{P­(OMe)_3_}_2_]^2–^ are terminally bonded to the two external Pt_3_ triangles (Figures 8 and 9, and Table 5). The intratriangular Pt–Pt bonding distances are almost identical in all the [Pt_12_(CO)_22_(L)_2_]^2–^ [L = P­(OPh)_3_, P­(OMe)_3_, PPh_3_, PPh_2_Py] species and in the homoleptic [Pt_12_(CO)_24_]^2–^ (Table 5).^5,6,9,16,67^ It is noteworthy that the spread of the intertriangular Pt–Pt bond distances increases in the series [Pt_12_(CO)_24_]^2–^ < [Pt_12_(CO)_22_{P­(OR)_3_}_2_]^2–^ (R = Ph, Me) < [Pt_12_(CO)_22_(PR_3_)_2_]^2–^ (PR_3_ = PPh_3_, PPh_2_Py). This is somehow related to the fact that organophosphite ligands are less bulky and more flexible than triarylphosphines.

**Figure 8:**
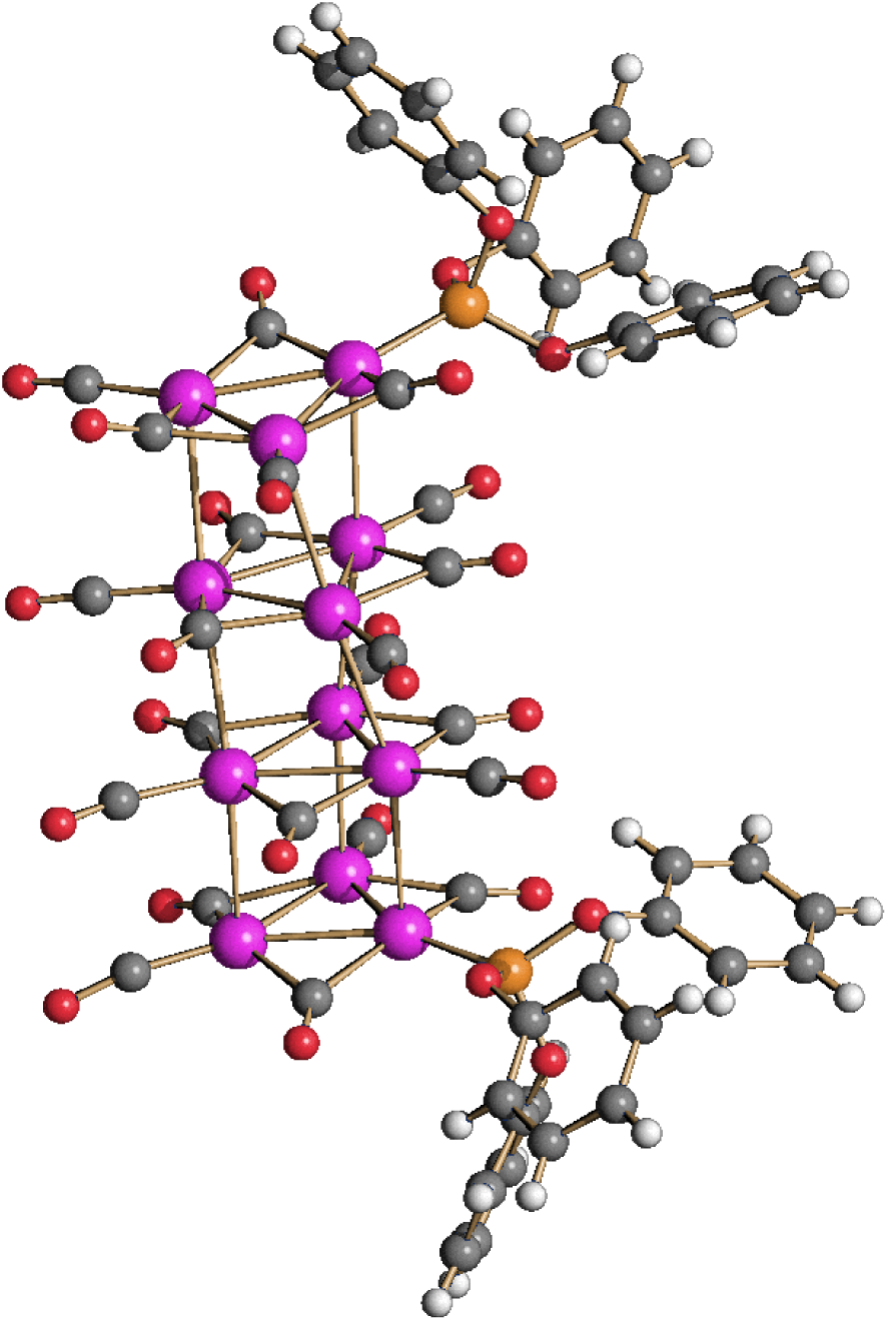
Molecular structure of [Pt_12_(CO)_22_{P­(OPh)_3_}_2_]^2–^ (purple, Pt; orange, P; red, O; gray, C; white, H).

**Figure 9:**
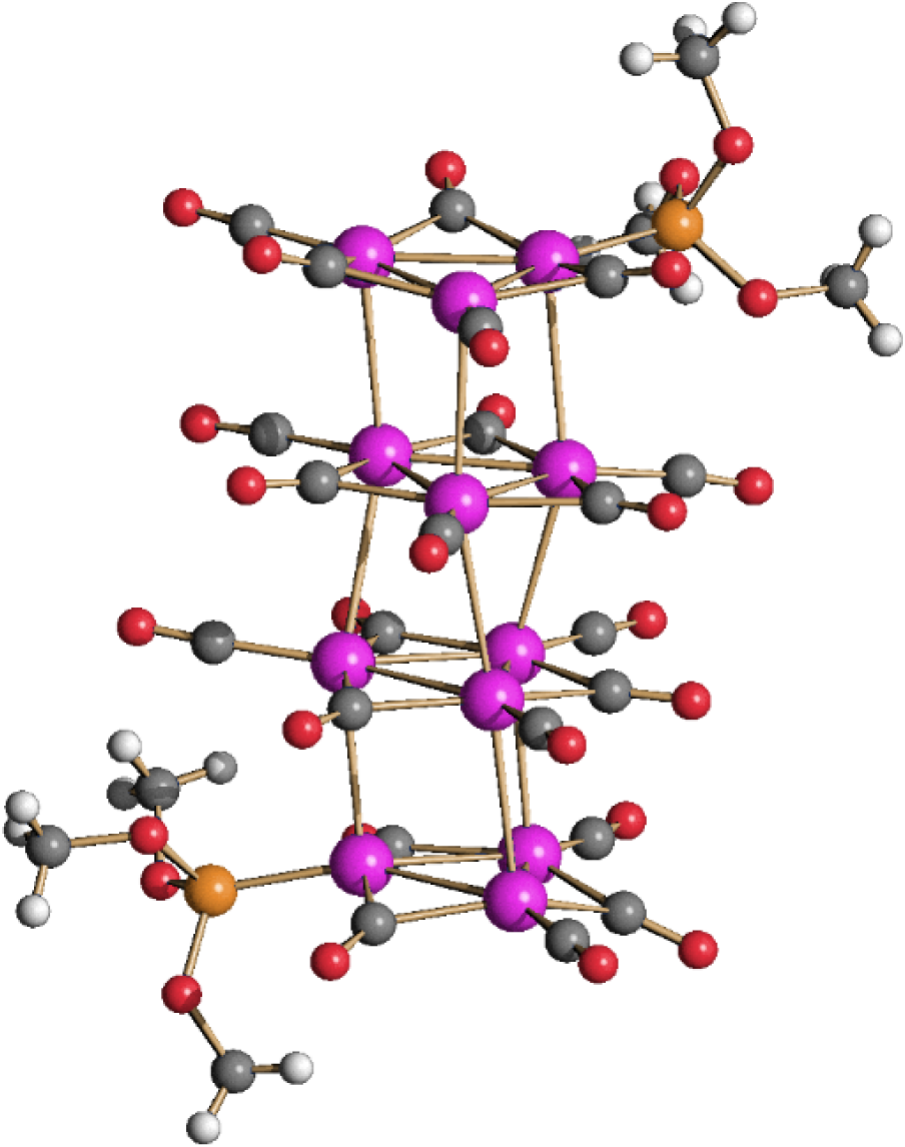
Molecular structure of [Pt_12_(CO)_22_{P­(OMe)_3_}_2_]^2–^ (purple, Pt; orange, P; red, O; gray, C; white, H).

The Pt–P bonds of [Pt_12_(CO)_22_{P­(OPh)_3_}_2_]^2–^ [2.189(5) and 2.198(5) Å] are slightly shorter than in [Pt_12_(CO)_22_{P­(OMe)_3_}_2_]^2–^ [2.204(3) and 2.218(3) Å], in keeping with the greater π-acidic character of P­(OPh)_3_ compared to P­(OMe)_3_.

The mutual arrangement of the P-donor ligands of [Pt_12_(CO)_22_{P­(OPh)_3_}_2_]^2–^, [Pt_12_(CO)_22_{P­(OMe)_3_}_2_]^2–^, [Pt_12_(CO)_22_(PPh_3_)_2_]^2–^ and [Pt_12_(CO)_22_(PPh_2_Py)_2_]^2–^ deserves some comments.^63,67^ A convenient way to represent the disposition of the P-ligands is to use the P_1_Ct_1_Ct_2_P_2_ torsion angle as defined in Scheme 3 (Ct_1_ and Ct_2_ are the centroids of the two external Pt_3_ triangles). The nomenclature used in organic chemistry for torsional isomers may be adapted to the present case.^69^ [Pt_12_(CO)_22_(PPh_3_)_2_]^2–^ and [Pt_12_(CO)_22_(PPh_2_Py)_2_]^2–^ display torsion angles close to −40° and may be defined as (−) synclinal torsional isomers. Even though significantly larger, the torsion angle of [Pt_12_(CO)_22_{P­(OPh)_3_}_2_]^2–^ [−72.21°] is also comprised in the range for a (−) synclinal arrangement [−30° to −90°]. In contrast, [Pt_12_(CO)_22_{P­(OMe)_3_}_2_]^2–^ displays a very large torsion angle [−145.16°] in the range for a (−) anticlinal conformation [−90° to −150°].

**Scheme 3:**
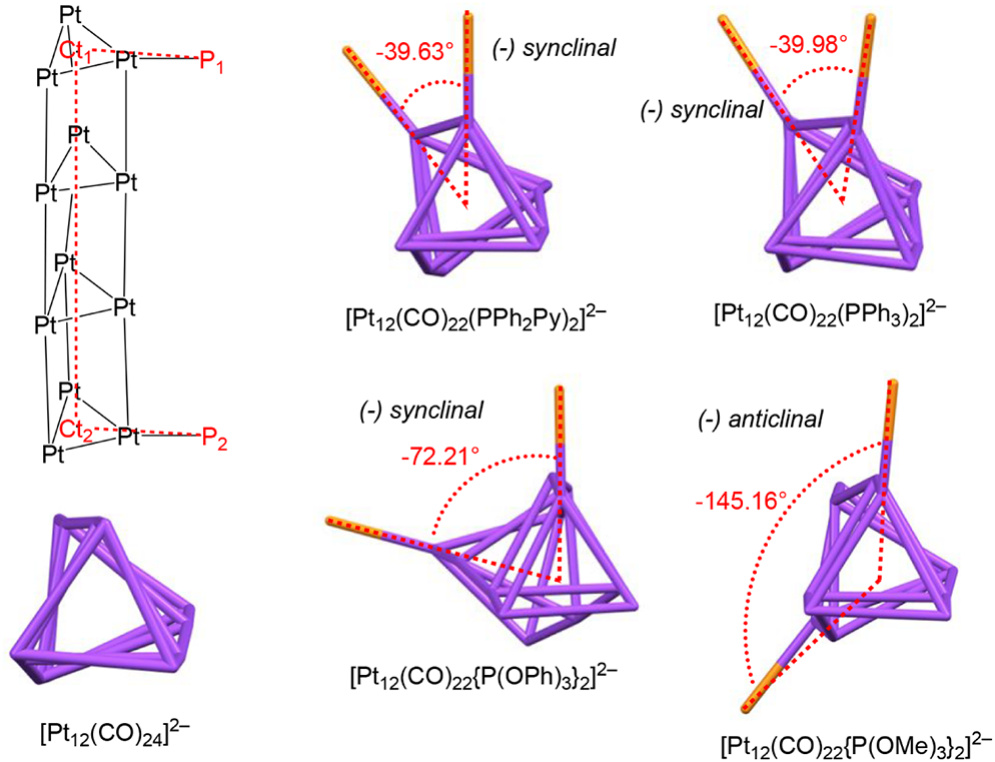
Representation of the Relative Disposition of the P-Ligands in the Species [Pt_12_(CO)_22_{P­(OPh)_3_}_2_]^2–^, [Pt_12_(CO)_22_{P­(OMe)_3_}_2_]^2–^, [Pt_12_(CO)_22_(PPh_3_)_2_]^2–^ and [Pt_12_(CO)_22_(PPh_2_Py)_2_]^2–^ [Fn sch3-fn3]

a The value of the P_1_Ct_1_Ct_2_P_2_ torsion angle is reported (purple, Pt; orange, P).

The different conformations displayed in the solid state by [Pt_12_(CO)_22_{P­(OR)_3_}_2_]^2–^ (R = Me, Ph) prompted a computational investigation starting from the SC-XRD data. The gas-phase DFT optimization of the two clusters in different arrangements revealed limited energy difference (ΔE) between the two conformations, as shown in Figure S60 in the Supporting Information. DFT calculations predicted slightly higher stability of the synclinal arrangement for both the clusters, but the small ΔE values are at the limit of accuracy of the computational method used. Moreover, the different configurations observed by SC-XRD are possibly related to the additional energy contribution from packing forces, not considered in the computational study. The mutual rotation between the two external [Pt_3_(CO)_2_(μ-CO)_3_{P­(OMe)_3_}] rings was also investigated for the P­(OMe)_3_ derivative. The energy profile obtained (Figure S61 in the Supporting Information) suggests low energy barriers for the torsional isomerization, a result in line with the ^31^P NMR spectra, where only one species is present on the NMR time scale at all temperatures considered (193–298 K).

As in the structures discussed above, also in the case of [Pt_15_(CO)_28_{P­(OPh)_3_}_2_]^2–^ the substitution occurs at the two external Pt_3_ triangles (Figure 10 and Table 5). The bonding parameters of [Pt_15_(CO)_28_{P­(OPh)_3_}_2_]^2–^ are very close to that of the parent [Pt_15_(CO)_30_]^2–^,^5,6,9,16,66^ indicating the P­(OPh)_3_/CO replacement at the external triangles does not significantly alter the Pt_15_ cage of this large cluster. In this case, the P_1_Ct_1_Ct_2_P_2_ torsion angle [21.17°] is in the range for a (+) synperiplanar isomer [0° to +30°].

**Figure 10:**
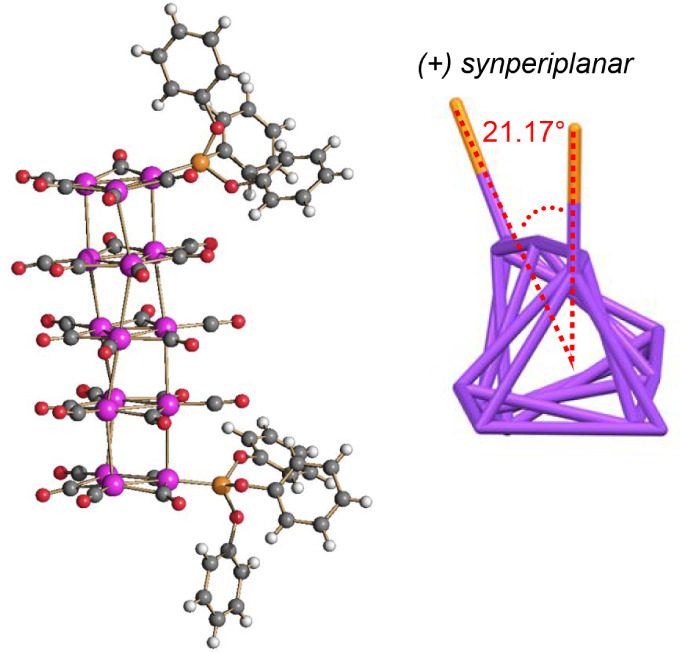
Molecular structure of [Pt_15_(CO)_28_{P­(OPh)_3_}_2_]^2–^ (purple, Pt; orange, P; red, O; gray, C; white, H), and schematic representation of the mutual arrangement of the two P­(OPh)_3_ ligands.

### 2.4 ESI-MS Studies

To get further insight into the reactions between [Pt_3n_(CO)_6n_]^2–^ (n = 3–5) and P­(OR)_3_, some ESI-MS studies have been performed (Figures S48–S53 and Tables S1–S6 in the Supporting Information). It must be remarked that homoleptic Chini clusters are partially oxidized and reduced during ionization with formation of some [Pt_3(n+1)_(CO)_6(n+1)_]^2–^ and [Pt_3(n–1)_(CO)_6(n–1)_]^2–^, respectively.^2,4^ In the case of heteroleptic clusters, previous studies on PPh_3_-derivatives of Chini clusters indicate that their ESI-MS spectra are further complicated by formation of poly substituted species during ionization, in addition to redox reactions.^67^ All these points have been confirmed in the present studies using organophosphites as ligands.

As an example, the ESI-MS spectrum of [Pt_9_(CO)_16_{P­(OPh)_3_}_2_]^2–^ in CH_3_CN solution (Figure 11 and Table 6), just obtained by mixing [Pt_9_(CO)_18_]^2–^ and two mole equivalents of P­(OPh)_3_, displays peaks attributable to the species (relative intensities in parentheses) [Pt_9_(CO)_18_]^2–^ (15), [Pt_9_(CO)_17_{P­(OPh)_3_}]^2–^ (50), [Pt_9_(CO)_16_{P­(OPh)_3_}_2_]^2–^ (65), [Pt_9_(CO)_15_{P­(OPh)_3_}_3_]^2–^ (8), [Pt_12_(CO)_24_]^2–^ (10), [Pt_12_(CO)_23_{P­(OPh)_3_}]^2–^ (50), [Pt_12_(CO)_22_{P­(OPh)_3_}_2_]^2–^ (100), [Pt_12_(CO)_21_{P­(OPh)_3_}_3_]^2–^ (65), [Pt_12_(CO)_20_{P­(OPh)_3_}_4_]^2–^ (15), [Pt_15_(CO)_29_{P­(OPh)_3_}]^2–^ (10) and [Pt_15_(CO)_28_{P­(OPh)_3_}_2_]^2–^ (5). Further examples may be found in the Supporting Information.

**Figure 11:**
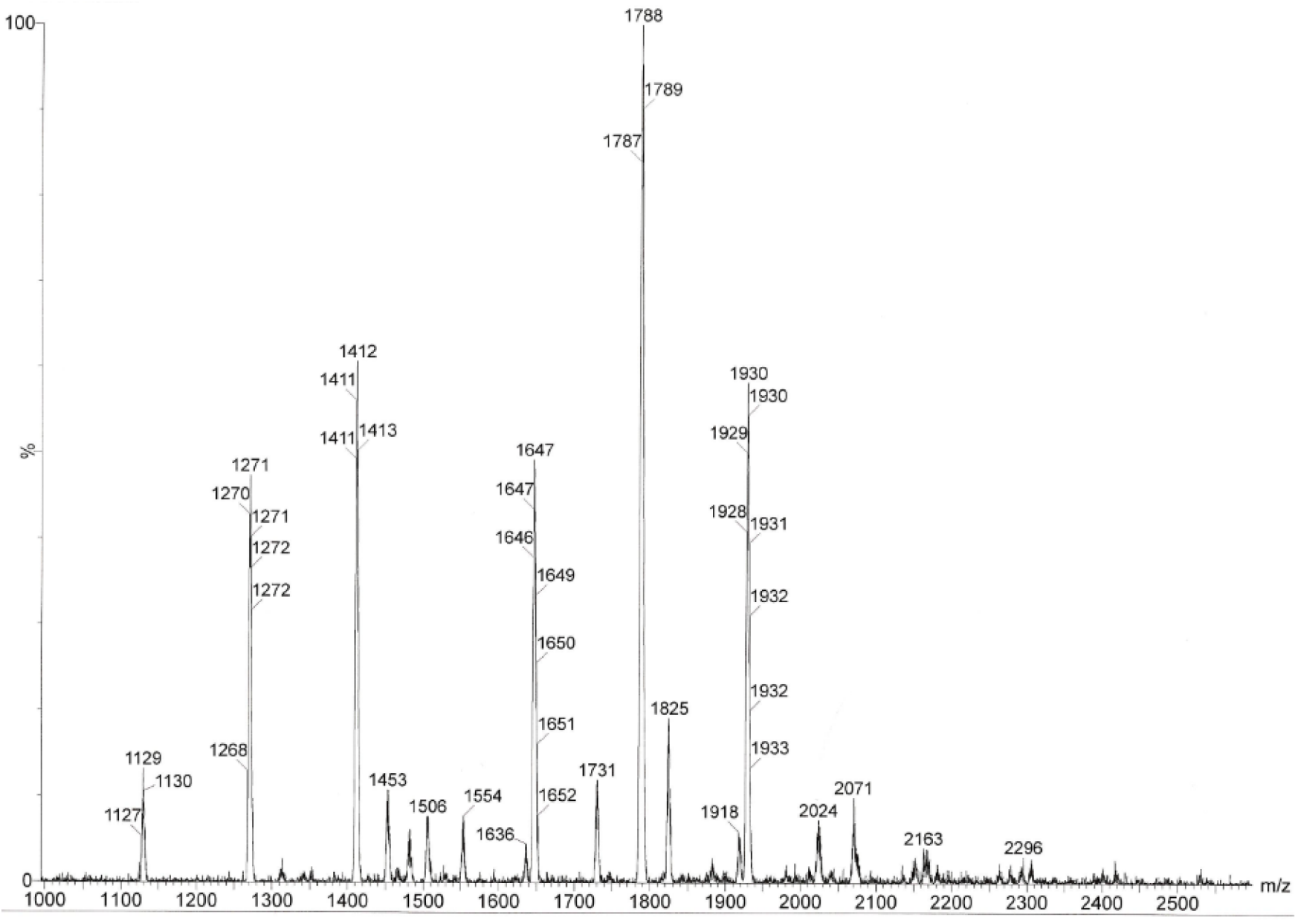
ESI-MS spectrum (relative intensity (%) vs *m*/*z*) in MeCN solution (ES-) of the crude of the reaction of [Pt_9_(CO)_18_]^2–^ with two mole equivalents of P­(OPh)_3_.

**Table 6:** Peak Assignment of the ESI-MS Spectrum of the Crude of the Reaction of [Pt_9_(CO)_18_]^2–^ with Two Mole Equivalents of P­(OPh)_3_

*m*/*z*	Relative intensity	Ion
1129	15	[Pt_9_(CO)_18_]^2–^
1271	50	[Pt_9_(CO)_17_{P(OPh)_3_}]^2–^
1412	65	[Pt_9_(CO)_16_{P(OPh)_3_}_2_]^2–^
1506	10	[Pt_12_(CO)_24_]^2–^
1554	8	[Pt_9_(CO)_15_{P(OPh)_3_}_3_]^2–^
1647	50	[Pt_12_(CO)_23_{P(OPh)_3_}]^2–^
1788	100	[Pt_12_(CO)_22_{P(OPh)_3_}_2_]^2–^
1930	65	[Pt_12_(CO)_21_{P(OPh)_3_}_3_]^2–^
2024	10	[Pt_15_(CO)_29_{P(OPh)_3_}]^2–^
2071	15	[Pt_12_(CO)_20_{P(OPh)_3_}_4_]^2–^
2163	5	[Pt_15_(CO)_28_{P(OPh)_3_}_2_]^2–^

As independently shown by FT-IR and ^31^P­{^1^H} NMR spectroscopy, some of the unsubstituted, mono- and disubstituted species are present in the starting solution. Conversely, more substituted and more oxidized clusters are likely to be formed during ionization. Even though such processes complicate the interpretation of the ESI-MS spectra of heteroleptic Chini clusters, which are not fully representative of the species present in solution, they suggest that, at least in the gas phase, poly substituted species with more than two P-ligands may be formed.

Formation of poly substituted species during ESI-MS analyses deserves some further comments. In the case of P­(OR)_3_ and PPh_3_ ligands, there is only spectroscopic (FT-IR and NMR) evidence in solution of mono- and disubstituted species, and this has been further corroborated by SC-XRD analyses. The only heteroleptic Chini clusters containing more than two P-ligands spectroscopically characterized in solution are [Pt_9_(CO)_15_(PTA)_3_]^2–^, [Pt_12_(CO)_20_(PTA)_4_]^2–^ and [Pt_15_(CO)_25_(PTA)_5_]^2–^; the latter two species have been also structurally characterized by SC-XRD.^64^ [Pt_9_(CO)_15_(PTA)_3_]^2–^ has been obtained by reacting [Pt_9_(CO)_18_]^2–^ and PTA, while [Pt_12_(CO)_20_(PTA)_4_]^2–^ and [Pt_15_(CO)_25_(PTA)_5_]^2–^ resulted from the oxidation of [Pt_9_(CO)_15_(PTA)_3_]^2–^. In all these poly substituted species, there is one PTA ligand per Pt_3_ triangle, including external and internal ones. Based on the Tolman cone angle (PTA 103°, P­(OMe)_3_ 107°, P­(OPh)_3_ 128°, PPh_3_ 145°), PTA is the least sterically demanding ligand in the series.^70,71^ At the same time, PTA is the strongest σ-base and weakest π-acid.

### 2.5 Computational Studies of Positional Isomerism in P­(OR)_3_-Substituted Chini Clusters

The relative energies of the possible positional isomers of [Pt_9_(CO)_17_{P­(OMe)_3_}]^2–^, [Pt_9_(CO)_16_{P­(OMe)_3_}_2_]^2–^ and [Pt_9_(CO)_15_{P­(OMe)_3_}_3_]^2–^ were initially investigated by means of DFT calculations with the PBEh-3c method. Trimethylphosphite was chosen as ligand not only to facilitate the computational simulations, but also because the Tolman angle of P­(OMe)_3_ (107°) is close to that of PTA (103°), the only P-donor ligand for which a trisubstituted {Pt_9_} heteroleptic Chini cluster, [Pt_9_(CO)_15_(PTA)_3_]^2–^, has been spectroscopically characterized in solution.^64^ For clarity, the substitutions on the central or on the external {Pt_3_} triangles will be indicated with the *c-* and *e-* prefixes, respectively, in the following discussion. If two different external triangles are substituted, the *e-* and *e*′*-* prefixes will be used.

The monosubstituted [Pt_9_(CO)_17_{P­(OMe)_3_}]^2–^ cluster can have two positional isomers, [Pt_9_(CO)_17_{*e-*P­(OMe)_3_}]^2–^ and [Pt_9_(CO)_17_{*c-*P­(OMe)_3_}]^2–^. The energy difference between the two species indicates that the substitution at the external triangles is more favored (Figure S62 in the Supporting Information), supporting the observation of the *e*-isomer of [Pt_9_(CO)_17_{P­(OPh)_3_}]^2–^ by means of SC-XRD. Such a result was confirmed by TPSS0/def2-TVZP calculations (caption of Figure S62 in the Supporting Information). It is worth noting that the arrangement of one of the external triangles in the DFT-optimized structure of [Pt_9_(CO)_17_{*c-*P­(OMe)_3_}]^2–^ is very far from the stacking along a common *pseudo*-*C*_3_ axis present in the unsubstituted Chini cluster.

In the case of the disubstituted [Pt_9_(CO)_16_{P­(OMe)_3_}_2_]^2–^ cluster, the two phosphites can be on the same triangle, with the formation of the [Pt_9_(CO)_16_{*e-*P­(OMe)_3_}_2_]^2–^ and [Pt_9_(CO)_16_{*c-*P­(OMe)_3_}_2_]^2–^ isomers, or on two different triangles, leading to the [Pt_9_(CO)_16_{*e-*P­(OMe)_3_}­{*c-*P­(OMe)_3_}]^2–^ and [Pt_9_(CO)_16_{*e-*P­(OMe)_3_}­{*e*′*-*P­(OMe)_3_}]^2–^ isomers (Figure S63 in the Supporting Information). Torsional isomerism is also present for the last two species, and it is relevant for [Pt_9_(CO)_16_{*e-*P­(OMe)_3_}­{*c-*P­(OMe)_3_}]^2–^, as shown in Figure S64 in the Supporting Information. The energy comparison among the positional isomers using different computational methods and including implicit solvation did not allow to unequivocally determine the most stable species among [Pt_9_(CO)_16_{*e-*P­(OMe)_3_}_2_]^2–^, [Pt_9_(CO)_16_{*e-*P­(OMe)_3_}­{*e′-*P­(OMe)_3_}]^2–^ and the staggered isomer of [Pt_9_(CO)_16_{*e-*P­(OMe)_3_}­{*c-*P­(OMe)_3_}]^2–^ (Figure S63 and Table S8 in the Supporting Information), despite the fact that the experimental outcomes indicate the exclusive formation of [Pt_9_(CO)_16_{*e-*P­(OMe)_3_}­{*e′-*P­(OMe)_3_}]^2–^. Nonetheless, these results somehow agree with the fact that bidentate ligands have been found bridging one external and one central triangle.^65,66^ The double substitution on the central triangle appears thermodynamically unfavored, since [Pt_9_(CO)_16_{*c-*P­(OMe)_3_}_2_]^2–^ is meaningfully less stable than [Pt_9_(CO)_16_{*e-*P­(OMe)_3_}_2_]^2–^.

Selected isomers of the P­(OPh)_3_ derivatives were also compared at C-PCM/PBEh-3c level, and the energy difference between [Pt_9_(CO)_16_{*e-*P­(OPh)_3_}_2_]^2–^ and [Pt_9_(CO)_16_{*e-*P­(OPh)_3_}­{*e′-*P­(OPh)_3_}]^2–^ resulted negligible. The experimental evidence of only one species in solution appears hardly explainable only on the relative thermodynamic stability of the isomers. It is likely to suppose that the substitution reactions on [Pt_9_(CO)_18_]^2–^ by organophosphites are at least in part kinetically controlled.

For completeness, the possible positional isomers of [Pt_9_(CO)_15_{P­(OMe)_3_}_3_]^2–^ were also considered to better investigate this type of isomerism in enneanuclear derivatives of platinum Chini clusters (Figure S65 in the Supporting Information). PBEh-3c calculations were carried out on the staggered geometries of the positional isomers [Pt_9_(CO)_15_{*e-*P­(OMe)_3_}_3_]^2–^, [Pt_9_(CO)_15_{*e-*P­(OMe)_3_}_2_{*c-*P­(OMe)_3_}]^2–^, [Pt_9_(CO)_15_{*e-*P­(OMe)_3_}_2_{*e′-*P­(OMe)_3_}]^2–^, [Pt_9_(CO)_15_{*e-*P­(OMe)_3_}­{*c-*P­(OMe)_3_}_2_]^2–^, [Pt_9_(CO)_15_{*e-*P­(OMe)_3_}­{*c-*P­(OMe)_3_}­{*e′-*P­(OMe)_3_}_3_]^2–^ and [Pt_9_(CO)_15_{*c-*P­(OMe)_3_}_3_]^2–^. The energy values of [Pt_9_(CO)_15_{*e-*P­(OMe)_3_}_3_]^2–^, [Pt_9_(CO)_15_{*e-*P­(OMe)_3_}_2_{*e′-*P­(OMe)_3_}]^2–^ and [Pt_9_(CO)_15_{*e-*P­(OMe)_3_}_2_{*c-*P­(OMe)_3_}]^2–^ are very similar, as expected from the results obtained for the disubstituted clusters. Moreover, [Pt_9_(CO)_15_{*e-*P­(OMe)_3_}­{*c-*P­(OMe)_3_}­{*e′-*P­(OMe)_3_}]^2–^ resulted only slightly less stable than [Pt_9_(CO)_15_{*e-*P­(OMe)_3_}_2_{*c-*P­(OMe)_3_}]^2–^. On the other hand, the presence of more than one phosphite on the central triangle is unfavored, as revealed by the relative energy values of [Pt_9_(CO)_15_{*e-*P­(OMe)_3_}­{*c-*P­(OMe)_3_}_2_]^2–^ and [Pt_9_(CO)_15_{*c-*P­(OMe)_3_}_3_]^2–^ (Figure S65 in the Supporting Information).

## 3 Conclusions

A detailed chemical, spectroscopic, structural and computational study of the selective functionalization of [Pt_3n_(CO)_6n_]^2–^ (n = 3–5) Chini clusters with organophosphite P­(OR)_3_ (R = Me, Et, Ph) ligands has been herein reported. Heteroleptic [Pt_3n_(CO)_6n–x_{P­(OR)_3_}_*x*_]^2–^ (n = 3–5; x = 1, 2; R = Me, Et, Ph) Chini clusters have been synthesized and spectroscopically characterized. The molecular structures of [Pt_9_(CO)_17_{P­(OPh)_3_}]^2–^, [Pt_12_(CO)_22_{P­(OPh)_3_}_2_]^2–^, [Pt_12_(CO)_22_{P­(OMe)_3_}_2_]^2–^ and [Pt_15_(CO)_28_{P­(OPh)_3_}_2_]^2–^ have been determined by SC-XRD on miscellaneous salts.

The experimental evidence points out that CO substitution selectively takes place at the external Pt_3_ triangles. First, one terminal CO is replaced in one external triangle leading to [Pt_3n_(CO)_6n–1_{*e*-P­(OR)_3_}]^2–^ and then, the second substitution occurs on the opposite external triangle affording [Pt_3n_(CO)_6n–2_{*e*-P­(OR)_3_}­{*e*′-P­(OR)_3_}]^2–^. DFT calculations on the possible positional isomers of disubstituted Pt_9_ clusters suggested that the structure of the final cluster cannot be rationalized only by means of considerations regarding the relative stability of the species. In addition, it must be remarked that previous results indicated an associative mechanism for CO-substitution in Chini clusters.^63^ It is likely that addition of a second P-donor ligand to the same triangle would be somehow unfavored due to steric and electronic reasons.

The addition of a third P­(OR)_3_ ligand causes the elimination of a Pt_3_ triangle, as Pt(0) CO/P­(OR)_3_ complexes, and formation of a reduced [Pt_3(n–1)_(CO)_6(n–1)_]^2–^ cluster, which can be then further substituted. There is no spectroscopic nor SC-XRD evidence of species containing more than two P­(OR)_3_ ligands in solution or in the solid state. Conversely, poly substituted species [Pt_3n_(CO)_6n–x_{P­(OR)_3_}_*x*_]^2–^ (*x* ≥ 3) have been observed in the gas phase during ESI-MS experiments on mono- and disubstituted clusters. It is likely that intercluster rearrangements occur in the gas phase, and indeed mixtures of mono-, di-, poly and nonsubstituted clusters have been detected during ESI-MS experiments, even starting from a single species in solution. It must be remarked that ligand exchange has not been observed in solution, at least in the NMR time scale.

Besides positional isomerism, also torsional isomers have been observed in the case of disubstituted clusters. Indeed, both synclinal and anticlinal conformations have been observed in the solid state by SC-XRD for [Pt_12_(CO)_22_(L)_2_]^2–^ (L = PPh_3_, PPh_2_Py, P­(OMe)_3_, P­(OPh)_3_) species, whereas [Pt_15_(CO)_28_{P­(OPh)_3_}_2_]^2–^ adopts a synperiplanar conformation. The computed energy barriers for the torsional isomerization are rather low, suggesting free rotation of the substituted triangles in solution. Thus, the different conformations observed in the solid state are likely to be due to packing forces.

The results herein reported highlight analogies but also some significant differences comparing the behavior of organosphosphite and phosphine ligands toward Chini clusters. Indeed, the behavior of organophosphites P­(OR)_3_ (R = Me, Et, Ph) toward Chini clusters resembles that of PPh_3_ when [Pt_9_(CO)_18_]^2–^ and [Pt_12_(CO)_24_]^2–^ are considered. Moreover, also the reactions of P­(OMe)_3_ and P­(OEt)_3_ with [Pt_15_(CO)_30_]^2–^ are like those observed with PPh_3_, resulting in its reduction to [Pt_12_(CO)_24_]^2–^. Conversely, the mono- and disubstituted products [Pt_15_(CO)_29_{P­(OPh)_3_}]^2–^ and [Pt_15_(CO)_28_{P­(OPh)_3_}_2_]^2–^ have been obtained employing P­(OPh)_3_. It is noteworthy that these represent the first examples of higher nuclearity Chini clusters which have been directly functionalized upon direct CO substitution. This is likely to be due to electronic rather than steric effects. Indeed, the Tolman cone angle of P­(OPh)_3_ (128°) is intermediate between that of PPh_3_ (145°) and those of P­(OMe)_3_ (107°) and P­(OEt)_3_ (109°).^70,71^ Conversely, P­(OPh)_3_ displays the highest electronic parameter (2086.1 cm^–1^) compared to PPh_3_ (2068.9 cm^–1^), P­(OMe)_3_ (2079.5 cm^–1^) and P­(OEt)_3_ (2076.3 cm^–1^).^70,71^ This indicates that P­(OPh)_3_ is a stronger π-acid and weaker σ-base compared to the other three ligands, favoring CO substitution rather than reduction of [Pt_15_(CO)_30_]^2–^. In the case of more reduced clusters such as [Pt_9_(CO)_18_]^2–^ and [Pt_12_(CO)_24_]^2–^, further reduction is more difficult and, therefore, similar substitution products can be obtained with all the ligands considered.

PTA is the least sterically demanding (Tolman cone angle 103°) and most basic (electronic parameter 2064.4 cm^–1^) ligand compared to P­(OR)_3_ (R = Me, Et, Ph) and PPh_3_. PTA reduces [Pt_15_(CO)_30_]^2–^ as the other basic ligands, and results in mono- and disubstitution when reacted with [Pt_9_(CO)_18_]^2–^ and [Pt_12_(CO)_24_]^2–^. In view of its reduced cone angle, it has been possible also to spectroscopically detect in solution the trisubstituted cluster [Pt_9_(CO)_15_(PTA)_3_]^2–^ upon reaction of [Pt_9_(CO)_18_]^2–^ with three mole equivalents of PTA. Moreover, controlled oxidation of [Pt_9_(CO)_15_(PTA)_3_]^2–^ afforded the poly substituted Chini clusters [Pt_12_(CO)_20_(PTA)_4_]^2–^ and [Pt_15_(CO)_25_(PTA)_5_]^2–^, which are not accessible by direct substitution. The latter two clusters have been structurally characterized by SC-XRD, showing that there is one terminal PTA ligand per Pt_3_ triangle.

In conclusion, 50 years since they discovery, platinum Chini carbonyl clusters still show a rich and variegated chemistry. Several ligands may be introduced without altering their unique 1-D structure, and the products obtained result from a subtle balance of steric and electronic properties of the ligands, as well as the stoichiometry of the reaction. Further developments might be achieved using chiral ligands or ligands suitable for cluster anchoring on supports, widening the scope of the applications of Chini clusters.^14–37^

## 4 Experimental Section

### 4.1 General Procedures

All reactions and sample manipulations were carried out using standard Schlenk techniques under nitrogen and/or carbon monoxide atmosphere and in dried solvents. All the reagents were commercial products (Sigma-Aldrich, Merk, VWR) of the highest purity available and used as received, except [CAT]_2_[Pt_3n_(CO)_6n_] (n = 2–5; [CAT]^+^ = [PMePh_3_]^+^, [NEt_4_]^+^, [NBu_4_]^+^) which have been prepared according to the literature.^1^ Analyses of C, H and N were obtained with a Thermo Quest Flash EA 1112NC instrument. FT-IR spectra were recorded on a PerkinElmer Spectrum One interferometer in CaF_2_ cells. ^1^H and ^31^P­{^1^H} NMR measurements were performed on Bruker Avance III 600 MHz instruments. The proton chemical shift was referenced to the nondeuterated aliquot of the solvent. The phosphorus chemical shifts were referenced to external H_3_PO_4_ (85% in D_2_O). ESI mass spectra were recorded on a Waters Micromass ZQ4000 instrument using CH_3_CN as solvent (Source Temperature = 150 °C; Capillary Voltage = 2.54 kV; Infusion Flow = 20 μL/min; Cone Voltage = 10 V). Structure drawings have been performed with Shakal.^72^

### 4.2 Stepwise Reaction of [CAT]_2_[Pt_12_(CO)_24_] with P­(OR)_3_ (R = Me, Et; [CAT]^+^ = [PMePh_3_]^+^, [NEt_4_]^+^, [NBu_4_]^+^)


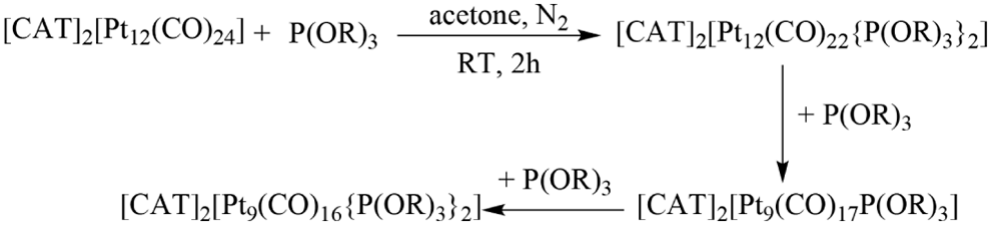


The stepwise reactions of [CAT]_2_[Pt_12_(CO)_24_] with P­(OR)_3_ (R = Me, Et) were studied in acetone by adding increasing amounts of P­(OR)_3_ to an acetone solution of the cluster and monitoring the progress of the reaction via FT-IR. As an example, the experimental procedure for the reaction of [PMePh_3_]_2_[Pt_12_(CO)_24_] will be given. Liquid P­(OR)_3_ was added in small portions (1 eq, 0.168 mmol, per time) to an acetone (30 mL) solution of [PMePh_3_]_2_[Pt_12_(CO)_24_] (0.600 g, 0.168 mmol). The solution was stirred at room temperature under nitrogen after each addition for 30 min and then, its outcome checked via FT-IR in the same solvent. The different spectra recorded are reported in Figures 3 and 4. The same procedure was employed for all the clusters of the [PMePh_3_]_2_[Pt_3n_(CO)_6n_] (n = 2–4) series with P­(OR)_3_ (R = Me, Et) affording analogous results. The outcome of these reactions does not depend on the cation employed and, thus, similar results have been obtained using [NEt_4_]^+^ and [NBu_4_]^+^ instead of [PMePh_3_]^+^.

### 4.3 Stepwise Reaction of [CAT]_2_[Pt_15_(CO)_30_] with P­(OPh)_3_ ([CAT]^+^ = [PMePh_3_]^+^, [NEt_4_]^+^, [NBu_4_]^+^)


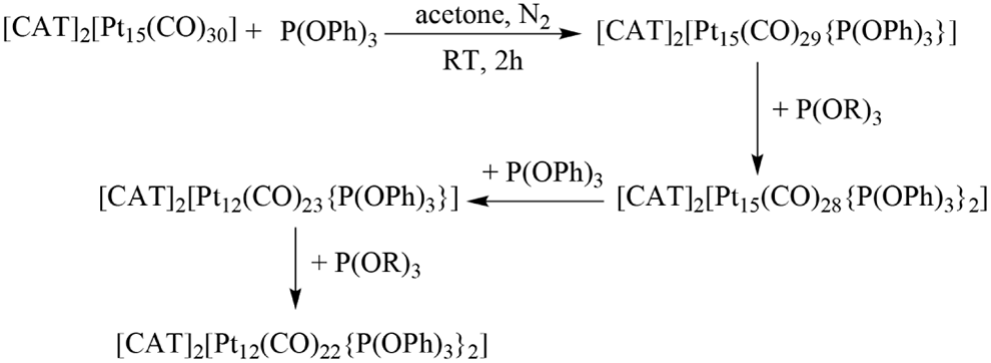


The stepwise reactions of [CAT]_2_[Pt_15_(CO)_30_] with P­(OPh)_3_ were studied in acetone by adding increasing amounts of P­(OPh)_3_ to an acetone solution of the cluster and monitoring the progress of the reaction via FT-IR. As an example, the experimental procedure for the reaction of [PMePh_3_]_2_[Pt_15_(CO)_30_] will be given. Liquid P­(OPh)_3_ was added in small portions (1 eq, 0.139 mmol, per time) to an acetone (30 mL) solution of [PMePh_3_]_2_[Pt_15_(CO)_30_] (0.600 g, 0.139 mmol). The solution was stirred at room temperature under nitrogen after each addition for 30 min and then, its outcome checked via FT-IR in the same solvent. The different spectra recorded are reported in Figure 1. The same procedure was employed for all the clusters of the [PMePh_3_]_2_[Pt_3n_(CO)_6n_] (n = 4, 5) series with P­(OPh)_3_ affording analogous results. The outcome of these reactions does not depend on the cation employed and, thus, similar results have been obtained using [NEt_4_]^+^ and [NBu_4_]^+^ instead of [PMePh_3_]^+^.

### 4.4 Synthesis of [PMePh_3_]_2_[Pt_15_(CO)_29_{P­(OPh)_3_}]

$${[{\mathrm{PMePh}}_{3}]}_{2}\left[{\mathrm{Pt}}_{15}{\left(\mathrm{CO}\right)}_{30}\right]{+P(\mathrm{OPh})}_{3}\overset{{\mathrm{acetone},N}_{2}}{\underset{\mathrm{RT},2h}{\to }}{[{\mathrm{PMePh}}_{3}]}_{2}\left[{\mathrm{Pt}}_{15}{\left(\mathrm{CO}\right)}_{29}{\{P(\mathrm{OPh})}_{3}\}\right]$$

Triphenyl phosphite (24.3 μL, 0.0926 mmol) was added to a solution of [PMePh_3_]_2_[Pt_15_(CO)_30_] (0.400 g, 0.0926 mmol) in acetone (25 mL). The resulting mixture was stirred for 2 h at room temperature. Then, the solvent was removed under reduced pressure and the residue washed with toluene (2 × 20 mL), 2-propanol (2 × 20 mL), *n*-hexane (3 × 10 mL) and finally extracted with acetone (15 mL). The presence of [PMePh_3_]_2_[Pt_15_(CO)_29_{P­(OPh)_3_}] in solution was corroborated by FT-IR, ^1^H and ^31^P­{^1^H} NMR spectroscopy analyses. A microcrystalline powder of [PMePh_3_]_2_[Pt_15_(CO)_29_{P­(OPh)_3_}] was obtained upon adding *n*-hexane (50 mL) to the acetone solution (0.362 g, yield 85% based on Pt).

**[PMePh_3_]_2_[Pt_15_(CO)_29_{P­(OPh)_3_}]**. C_85_H_51_O_32_P_3_Pt_15_ (4603.39): calcd. (%): C 22.18, H 1.12; found: C 22.41, H 1.35. FT-IR (acetone, 298 K) ν_CO_: 2050(s), 1864­(m) cm^–1^. ^1^H NMR (acetone, 298 K) δ_H_: 7.95–7.79 (m, CH_aryl_, cation), 7.28–7.09 (m, CH_aryl_, ligand), 3.20 (d, ^2^J_H–P_ 14 Hz, CH_3_, cation) ppm. ^31^P­{^1^H} NMR (acetone, 298 K) δ_P_: 140.1 ppm (m, ^1^J_Pt–P_ 8706 Hz, ^2^J_Pt–P_ 983 Hz, ligand), 22.1 (s, cation) ppm.

### 4.5 Synthesis of [PMePh_3_]_2_[Pt_15_(CO)_28_{P­(OPh)_3_}_2_]·2CH_3_COCH_3_·C_6_H_14_

$${[{\mathrm{PMePh}}_{3}]}_{2}\left[{\mathrm{Pt}}_{15}{\left(\mathrm{CO}\right)}_{30}\right]{+P(\mathrm{OPh})}_{3}\overset{{\mathrm{acetone},N}_{2}}{\underset{\mathrm{RT},2h}{\to }}{[{\mathrm{PMePh}}_{3}]}_{2}\left[{\mathrm{Pt}}_{15}{\left(\mathrm{CO}\right)}_{28}{{\{P(\mathrm{OPh})}_{3}\}}_{2}\right]$$

Triphenyl phosphite (47.2 μL, 0.180 mmol) was added in two portions, over a period of 2 h, to a solution of [PMePh_3_]_2_[Pt_15_(CO)_30_] (0.390 g, 0.0902 mmol) in acetone (20 mL). The resulting mixture was stirred at room temperature for 2 h. Then, the solvent was removed under reduced pressure and the residue washed with toluene (2 × 20 mL), 2-propanol (2 × 20 mL), *n*-hexane (3 × 10 mL) and finally extracted with acetone (15 mL). Slow diffusion of *n*-hexane (50 mL) on the acetone solution afforded crystals of [PMePh_3_]_2_[Pt_15_(CO)_28_{P­(OPh)_3_}_2_]·2CH_3_COCH_3_·C_6_H_14_ suitable for SC-XRD analyses (0.308 g, yield 67% based on Pt).

**[PMePh_3_]_2_[Pt_15_(CO)_28_{P­(OPh)_3_}_2_]·2CH_3_COCH_3_·C_6_H_14_**. C_114_H_92_O_36_P_4_Pt_15_ (5088.10): calcd. (%): C 26.91, H 1.82; found: C 27.12, H 1.98. FT-IR (acetone, 298 K) ν_CO_: 2041(s), 1855­(m) cm^–1^. FT-IR (nujol mull, 298 K) ν_CO_: 2043(s), 1856­(m) cm^–1^. ^1^H NMR (acetone, 298 K) δ_H_: 7.93–7.77 (m, CH_aryl_, ligand), 7.27–7.06 (m, CH_aryl_, cation), 3.16 (d, ^2^J_H–P_ 14 Hz, CH_3_, cation) ppm. ^31^P­{^1^H} NMR (acetone, 298 K) δ_P_: 143.8 (^1^J_Pt–P_ 8755 Hz, ^2^J_Pt–P_ 987 Hz, ligand), 22.1 (s, cation) ppm.

### 4.6 Synthesis of [PMePh_3_]_2_[Pt_12_(CO)_23_{P­(OPh)_3_}]

$${[{\mathrm{PMePh}}_{3}]}_{2}\left[{\mathrm{Pt}}_{12}{\left(\mathrm{CO}\right)}_{24}\right]{+P(\mathrm{OPh})}_{3}\overset{{\mathrm{acetone},N}_{2}}{\underset{\mathrm{RT},2h}{\to }}{[{\mathrm{PMePh}}_{3}]}_{2}\left[{\mathrm{Pt}}_{12}{\left(\mathrm{CO}\right)}_{23}{\{P(\mathrm{OPh})}_{3}\}\right]$$

Triphenyl phosphite (27.5 μL, 0.105 mmol) was added to a solution of [PMePh_3_]_2_[Pt_12_(CO)_24_] (0.375 g, 0.105 mmol) in acetone (20 mL). The resulting mixture was stirred for 2 h at room temperature. Then, the solvent was removed under reduced pressure and the residue washed with toluene (2 × 20 mL), 2-propanol (2 × 20 mL), *n*-hexane (3 × 10 mL) and finally extracted with acetone (15 mL). A microcrystalline powder of [PMePh_3_]_2_[Pt_12_(CO)_23_{P­(OPh)_3_}] was obtained upon adding *n*-hexane (50 mL) to the acetone solution (0.331 g, yield 82% based on Pt).

#### [PMePh_3_]_2_[Pt_12_(CO)_23_{P­(OPh)_3_}]

C_79_H_51_O_26_P_3_Pt_12_ (3850.09): calcd. (%): C 24.64, H 1.34; found: C 24.28, H 1.52. FT-IR (acetone, 298 K) ν_CO_: 2042(s), 1859­(m) cm^–1^. ^1^H NMR (acetone, 298 K) δ_H_: 7.93–7.77 (m, CH_aryl_, cation), 7.25–7.03 (m, CH_aryl_, ligand), 3.15 (d, ^2^J_H–P_ 14 Hz, CH_3_, cation) ppm. ^31^P­{^1^H} NMR (acetone, 298 K) δ_P_: 144.7 (^1^J_Pt–P_ 8768 Hz, ^2^J_Pt–P_ 987 Hz), 22.1 (s, cation) ppm.

### 4.7 Synthesis of [PMePh_3_]_2_[Pt_12_(CO)_22_{P­(OMe)_3_}_2_]

$${[{\mathrm{PMePh}}_{3}]}_{2}\left[{\mathrm{Pt}}_{12}{\left(\mathrm{CO}\right)}_{24}\right]{+P(\mathrm{OMe})}_{3}\overset{{\mathrm{acetone},N}_{2}}{\underset{\mathrm{RT},2h}{\to }}{[{\mathrm{PMePh}}_{3}]}_{2}\left[{\mathrm{Pt}}_{12}{\left(\mathrm{CO}\right)}_{22}{{\{P(\mathrm{OMe})}_{3}\}}_{2}\right]$$

Trimethyl phosphite (23.8 μL, 0.202 mmol) was added in two portions, over a period of 2 h, to a solution of [PMePh_3_]_2_[Pt_12_(CO)_24_] (0.360 g, 0.101 mmol) in acetone (20 mL). The resulting mixture was stirred at room temperature. Then, the solvent was removed under reduced pressure and the residue washed with toluene (2 × 20 mL), 2-propanol (2 × 20 mL), *n*-hexane (3 × 10 mL) and finally extracted with acetone (15 mL). Slow diffusion of *n*-hexane (50 mL) on the acetone solution afforded crystals of [PMePh_3_]_2_[Pt_12_(CO)_22_{P­(OMe)_3_}_2_] suitable for SC-XRD analyses (0.285 g, yield 75% based on Pt).

**[PMePh_3_]_2_[Pt_12_(CO)_22_{P­(OMe)_3_}_2_]**. C_66_H_54_O_28_P_4_Pt_12_ (3760.04): calcd. (%): C 21.08, H 1.45; found: C 21.19, H 1.29. FT-IR (acetone, 298 K) ν_CO_: 2038(s), 1853­(m) cm^–1^. FT-IR (nujol mull, 298 K) ν_CO_: 2034(s), 1841­(m) cm^–1^. ^1^H NMR (acetone, 298 K) δ_H_: 7.95–7.79 (m, CH_aryl_, cation), 3.66 (m, OCH_3_, ligand), 3.20 (d, ^2^J_H–P_ 14 Hz, CH_3_, cation) ppm. ^31^P­{^1^H} NMR (acetone, 298 K) δ_P_: 163.8 (^1^J_Pt–P_ 8071 Hz, ^2^J_Pt–P_ 861 Hz), 22.1 (s, cation) ppm.

### 4.8 Synthesis of [PMePh_3_]_2_[Pt_12_(CO)_22_{P­(OEt)_3_}_2_]

$${[{\mathrm{PMePh}}_{3}]}_{2}\left[{\mathrm{Pt}}_{12}{\left(\mathrm{CO}\right)}_{24}\right]{+P(\mathrm{OEt})}_{3}\overset{{\mathrm{acetone},N}_{2}}{\underset{\mathrm{RT},2h}{\to }}{[{\mathrm{PMePh}}_{3}]}_{2}\left[{\mathrm{Pt}}_{12}{\left(\mathrm{CO}\right)}_{22}{{\{P(\mathrm{OEt})}_{3}\}}_{2}\right]$$

Triethyl phosphite (34.3 μL, 0.200 mmol) was added in two portions, over a period of 2 h, to a solution of [PMePh_3_]_2_[Pt_12_(CO)_24_] (0.355 g, 0.100 mmol) in acetone (20 mL). The resulting mixture was stirred at room temperature. Then, the solvent was removed under reduced pressure and the residue washed with toluene (2 × 20 mL), 2-propanol (2 × 20 mL), *n*-hexane (3 × 10 mL) and finally extracted with acetone (15 mL). A microcrystalline powder of [PMePh_3_]_2_[Pt_12_(CO)_22_{P­(OEt)_3_}_2_] was obtained upon adding *n*-hexane (50 mL) to the acetone solution (0.288 g, yield 75% based on Pt).

**[PMePh_3_]_2_[Pt_12_(CO)_22_{P­(OEt)_3_}_2_]**. C_72_H_66_O_28_P_4_Pt_12_ (3844.11): calcd. (%): C 22.50, H 1.73; found: C 22.31, H 1.96. FT-IR (acetone, 298 K) ν_CO_: 2039(s), 1854­(m) cm^–1^. ^1^H NMR (acetone, 298 K) δ_H_: 7.95–7.79 (m, CH_aryl_, cation), 4.07 (m, OCH_2_, ligand), 3.21 (d, ^2^J_H–P_ 14 Hz, CH_3_, cation), 1.26 (t, ^2^J_H–P_ 13 Hz, CH_3_, ligand) ppm. ^31^P­{^1^H} NMR (acetone, 298 K) δ_P_: 158.0 (^1^J_Pt–P_ 7998 Hz, ^2^J_Pt–P_ 851 Hz), 22.1 (s, cation) ppm.

### 4.9 Synthesis of [PMePh_3_]_2_[Pt_12_(CO)_22_{P­(OPh)_3_}_2_]·solv

$${[{\mathrm{PMePh}}_{3}]}_{2}\left[{\mathrm{Pt}}_{12}{\left(\mathrm{CO}\right)}_{24}\right]{+P(\mathrm{OPh})}_{3}\overset{{\mathrm{acetone},N}_{2}}{\underset{\mathrm{RT},2h}{\to }}{[{\mathrm{PMePh}}_{3}]}_{2}\left[{\mathrm{Pt}}_{12}{\left(\mathrm{CO}\right)}_{22}{{\{P(\mathrm{OPh})}_{3}\}}_{2}\right]$$

Triphenyl phosphite (52.9 μL, 0.202 mmol) was added in two portions, over a period of 2 h, to a solution of [PMePh_3_]_2_[Pt_12_(CO)_24_] (0.360 g, 0.101 mmol) in acetone (20 mL). The resulting mixture was stirred at room temperature. Then, the solvent was removed under reduced pressure and the residue washed with toluene (2 × 20 mL), 2-propanol (2 × 20 mL), *n*-hexane (3 × 10 mL) and finally extracted with acetone (15 mL). Slow diffusion of *n*-hexane (50 mL) on the acetone solution afforded crystals of [PMePh_3_]_2_[Pt_12_(CO)_22_{P­(OPh)_3_}_2_]·solv suitable for SC-XRD analyses (0.285 g, yield 75% based on Pt).

**[PMePh_3_]_2_[Pt_12_(CO)_22_{P­(OPh)_3_}_2_]·solv**. C_96_H_66_O_28_P_4_Pt_12_ (4132.44): calcd. (%): C 27.90, H 1.61; found: C 27.75, H 1.94. FT-IR (acetone, 298 K) ν_CO_: 2037(s), 1854­(m) cm^–1^. FT-IR (nujol mull, 298 K) ν_CO_: 2034(s), 1841­(m) cm^–1^. ^1^H NMR (acetone, 298 K) δ_H_: 7.94–7.78 (m, CH_aryl_, cation), 7.24–7.16 (m, CH_aryl_, ligand), 3.17 (d, ^2^J_H–P_ 14 Hz, CH_3_, cation) ppm. ^31^P­{^1^H} NMR (acetone, 298 K) δ_P_: 148.1 (^1^J_Pt–P_ 8778 Hz, ^2^J_Pt–P_ 992 Hz), 22.1 (s, cation) ppm.

### 4.10 Synthesis of [PMePh_3_]_2_[Pt_9_(CO)_17_{P­(OMe)_3_}]

$${[{\mathrm{PMePh}}_{3}]}_{2}\left[{\mathrm{Pt}}_{9}{\left(\mathrm{CO}\right)}_{18}\right]{+P(\mathrm{OMe})}_{3}\overset{{\mathrm{acetone},N}_{2}}{\underset{\mathrm{RT},2h}{\to }}{[{\mathrm{PMePh}}_{3}]}_{2}\left[{\mathrm{Pt}}_{9}{\left(\mathrm{CO}\right)}_{17}{\{P(\mathrm{OPh})}_{3}\}\right]$$

Trimethyl phosphite (13.8 μL, 0.117 mmol) was added to a solution of [PMePh_3_]_2_[Pt_9_(CO)_18_] (0.380 g, 0.117 mmol) in acetone (20 mL). The resulting mixture was stirred for 2 h at room temperature. Then, the solvent was removed under reduced pressure and the residue washed with toluene (2 × 20 mL), 2-propanol (2 × 20 mL), *n*-hexane (3 × 10 mL) and finally extracted with acetone (15 mL). A microcrystalline powder of [PMePh_3_]_2_[Pt_9_(CO)_17_{P­(OMe)_3_}] was obtained upon adding *n*-hexane (50 mL) to the acetone solution (0.275 g, yield 85% based on Pt).

**[PMePh_3_]_2_[Pt_9_(CO)_17_{P­(OMe)_3_}]**. C_58_H_45_O_20_P_3_Pt_9_ (2910.59): calcd. (%): C 23.93, H 1.56; found: C 24.11, H 1.71. FT-IR (acetone, 298 K) ν_CO_: 2025(s), 1834­(m) cm^–1^. ^1^H NMR (acetone, 298 K) δ_H_: 7.94–7.78 (m, CH_aryl_, cation), 3.64 (m, CH_3_, ligand), 3.19 (d, ^2^J_H–P_ 14 Hz, CH_3_, cation) ppm. ^31^P­{^1^H} NMR (acetone, 298 K) δ_P_: 169.8 (^1^J_Pt–P_ 8053 Hz, ^2^J_Pt–P_ 843 Hz), 22.1 (s, cation) ppm.

### 4.11 Synthesis of [PMePh_3_]_2_[Pt_9_(CO)_17_{P­(OEt)_3_}]

$${[{\mathrm{PMePh}}_{3}]}_{2}\left[{\mathrm{Pt}}_{9}{\left(\mathrm{CO}\right)}_{18}\right]{+P(\mathrm{OEt})}_{3}\overset{{\mathrm{acetone},N}_{2}}{\underset{\mathrm{RT},2h}{\to }}{[{\mathrm{PMePh}}_{3}]}_{2}\left[{\mathrm{Pt}}_{9}{\left(\mathrm{CO}\right)}_{17}{\{P(\mathrm{OEt})}_{3}\}\right]$$

Triethyl phosphite (22.1 μL, 0.129 mmol) was added to a solution of [PMePh_3_]_2_[Pt_9_(CO)_18_] (0.385 g, 0.129 mmol) in acetone (20 mL). The resulting mixture was stirred for 2 h at room temperature. Then, the solvent was removed under reduced pressure and the residue washed with toluene (2 × 20 mL), 2-propanol (2 × 20 mL), *n*-hexane (3 × 10 mL) and finally extracted with acetone (15 mL). A microcrystalline powder of [PMePh_3_]_2_[Pt_9_(CO)_17_{P­(OEt)_3_] was obtained upon adding *n*-hexane (50 mL) to the acetone solution (0.275 g, yield 85% based on Pt).

**[PMePh_3_]_2_[Pt_9_(CO)_17_{P­(OEt)_3_}]**. C_61_H_51_O_20_P_3_Pt_9_ (2952.67): calcd. (%): C 24.81, H 1.74; found: C 24.58, H 1.95. FT-IR (acetone, 298 K) ν_CO_: 2024(s), 1835­(m) cm^–1^. ^1^H NMR (acetone, 298 K) δ_H_: 7.96–7.79 (m, CH_aryl_, cation), 4.07 (m, OCH_2_, ligand), 3.20 (d, ^2^J_H–P_ 14 Hz, CH_3_, cation), 1.26 (m, CH_3_, ligand) ppm. ^31^P­{^1^H} NMR (acetone, 298 K) δ_P_: 164.1 (^1^J_Pt–P_ 7079 Hz, ^2^J_Pt–P_ 845 Hz), 22.1 (s, cation) ppm.

### 4.12 Synthesis of [MePPh_3_]_2_[Pt_9_(CO)_17_{P­(OPh)_3_}]·CH_3_COCH_3_

$${[{\mathrm{PMePh}}_{3}]}_{2}\left[{\mathrm{Pt}}_{9}{\left(\mathrm{CO}\right)}_{18}\right]{+P(\mathrm{OPh})}_{3}\overset{{\mathrm{acetone},N}_{2}}{\underset{\mathrm{RT},2h}{\to }}{[{\mathrm{PMePh}}_{3}]}_{2}\left[{\mathrm{Pt}}_{9}{\left(\mathrm{CO}\right)}_{17}{\{P(\mathrm{OPh})}_{3}\}\right]$$

Triphenyl phosphite (36.4 μL, 0.139 mmol) was added to a solution of [PMePh_3_]_2_[Pt_9_(CO)_18_] (0.390 g, 0.139 mmol) in acetone (20 mL). The resulting mixture was stirred for 2 h at room temperature. Then, the solvent was removed under reduced pressure and the residue washed with toluene (2 × 20 mL), 2-propanol (2 × 20 mL), *n*-hexane (3 × 10 mL) and finally extracted with acetone (15 mL). Slow diffusion of *n*-hexane (50 mL) on the acetone solution afforded crystals of [PMePh_3_]_2_[Pt_9_(CO)_17_{P­(OPh)_3_}]·CH_3_COCH_3_ suitable for SC-XRD analyses (0.319 g, yield 73% based on Pt).

**[PMePh_3_]_2_[Pt_9_(CO)_17_{P­(OPh)_3_}]·CH_3_COCH_3_**. C_76_H_57_O_21_P_3_Pt_9_ (3154.93): calcd. (%): C 28.93, H 1.82; found: C 29.11, H 2.09. FT-IR (acetone, 298 K) ν_CO_: 2023(s), 1833­(m) cm^–1^. FT-IR (nujol mull, 298 K) ν_CO_: 2020(s), 1820­(m) cm^–1^. ^1^H NMR (acetone, 298 K) δ_H_: 7.92–7.76 (m, CH_aryl_, cation), 7.23–6.98 (m, CH_aryl_, ligand), 3.10 (d, ^2^J_H–P_ 14 Hz, CH_3_, cation) ppm. ^31^P­{^1^H} NMR (acetone, 298 K) δ_P_: 148.6 (^1^J_Pt–P_ 8810 Hz, ^2^J_Pt–P_ 992 Hz), 22.1 (s, cation) ppm.

### 4.13 Synthesis of [NEt_4_]_2_[Pt_9_(CO)_16_{P­(OMe)_3_}_2_]

$${[{\mathrm{NEt}}_{4}]}_{2}\left[{\mathrm{Pt}}_{9}{\left(\mathrm{CO}\right)}_{18}\right]{+P(\mathrm{OMe})}_{3}\overset{{\mathrm{acetone},N}_{2}}{\underset{\mathrm{RT},2h}{\to }}{[{\mathrm{NEt}}_{4}]}_{2}\left[{\mathrm{Pt}}_{9}{\left(\mathrm{CO}\right)}_{16}{{\{P(\mathrm{OMe})}_{3}\}}_{2}\right]$$

Trimethyl phosphite (36.5 μL, 0.310 mmol) was added in two portions, over a period of 2 h, to a solution of [NEt_4_]_2_[Pt_9_(CO)_18_] (0.390 g, 0.155 mmol) in acetone (20 mL). The resulting mixture was stirred at room temperature. Then, the solvent was removed under reduced pressure and the residue washed with toluene (2 × 20 mL), 2-propanol (2 × 20 mL), *n*-hexane (3 × 10 mL) and finally extracted with acetone (15 mL). A microcrystalline powder of [NEt_4_]_2_[Pt_9_(CO)_16_{P­(OMe)_3_}_2_] was obtained upon adding *n*-hexane (50 mL) to the acetone solution (0.336 g, yield 80% based on Pt).

**[NEt_4_]_2_[Pt_9_(CO)_16_{P­(OMe)_3_}_2_]**. C_38_H_58_N_2_O_22_P_2_Pt_9_ (2712.52): calcd. (%): C 16.82, H 2.16, N 1.03; found: C 16.94, H 1.95, N 1.21. FT-IR (acetone, 298 K) ν_CO_: 2016(s), 1826­(m) cm^–1^. ^1^H NMR (acetone, 298 K) δ_H_: 3.66 (m, CH_3_, ligand), 3.45 (q, CH_2_, cation), 1.36 (t, CH_3_, cation) ppm. ^31^P­{^1^H} NMR (acetone, 298 K) δ_P_: 171.5 (^1^J_Pt–P_ 8068 Hz, ^2^J_Pt–P_ 842 Hz) ppm.

### 4.14 Synthesis of [NEt_4_]_2_[Pt_9_(CO)_16_{P­(OEt)_3_}_2_]

$${[{\mathrm{NEt}}_{4}]}_{2}\left[{\mathrm{Pt}}_{9}{\left(\mathrm{CO}\right)}_{18}\right]{+P(\mathrm{OEt})}_{3}\overset{{\mathrm{acetone},N}_{2}}{\underset{\mathrm{RT},2h}{\to }}{[{\mathrm{NEt}}_{4}]}_{2}\left[{\mathrm{Pt}}_{9}{\left(\mathrm{CO}\right)}_{16}{{\{P(\mathrm{OEt})}_{3}\}}_{2}\right]$$

Triethyl phosphite (51.8 μL, 0.302 mmol) was added in two portions, over a period of 2 h, to a solution of [NEt_4_]_2_[Pt_9_(CO)_18_] (0.380 g, 0.151 mmol) in acetone (20 mL). The resulting mixture was stirred at room temperature. Then, the solvent was removed under reduced pressure and the residue washed with toluene (2 × 20 mL), 2-propanol (2 × 20 mL), *n*-hexane (3 × 10 mL) and finally extracted with acetone (15 mL). A microcrystalline powder of [NEt_4_]_2_[Pt_9_(CO)_16_{P­(OEt)_3_}_2_] was obtained upon adding *n*-hexane (50 mL) to the acetone solution (0.316 g, yield 75% based on Pt).

**[NEt_4_]_2_[Pt_9_(CO)_16_{P­(OEt)_3_}_2_]**. C_44_H_70_N_2_O_22_P_2_Pt_9_ (2796.68): calcd. (%): C 18.90, H 2.52, N 1.00; found: C 18.68, H 2.31, N 0.88. FT-IR (acetone, 298 K) ν_CO_: 2018(s), 1830­(m) cm^–1^. ^1^H NMR (acetone, 298 K) δ_H_: 4.08 (m, OCH_2_, ligand), 3.45 (q, CH_2_, cation), 1.36 (t, CH_3_, cation), 1.27 (m, CH_3_, ligand) ppm. ^31^P­{^1^H} NMR (acetone, 298 K) δ_P_: 165.6 (^1^J_Pt–P_ 7998 Hz, ^2^J_Pt–P_ 845 Hz) ppm.

### 4.15 Synthesis of [PMePh_3_]_2_[Pt_9_(CO)_16_{P­(OPh)_3_}_2_]

$${[{\mathrm{PMePh}}_{3}]}_{2}\left[{\mathrm{Pt}}_{9}{\left(\mathrm{CO}\right)}_{18}\right]{+P(\mathrm{OPh})}_{3}\overset{{\mathrm{acetone},N}_{2}}{\underset{\mathrm{RT},2h}{\to }}{[{\mathrm{PMePh}}_{3}]}_{2}\left[{\mathrm{Pt}}_{9}{\left(\mathrm{CO}\right)}_{16}{{\{P(\mathrm{OPh})}_{3}\}}_{2}\right]$$

Triphenyl phosphite (70.8 μL, 0.270 mmol) was added in two portions, over a period of 2 h, to a solution of [PMePh_3_]_2_[Pt_9_(CO)_18_] (0.380 g, 0.135 mmol) in acetone (20 mL). The resulting mixture was stirred at room temperature. Then, the solvent was removed under reduced pressure and the residue washed with toluene (2 × 20 mL), 2-propanol (2 × 20 mL), *n*-hexane (3 × 10 mL) and finally extracted with acetone (15 mL). A microcrystalline powder of [PMePh_3_]_2_[Pt_9_(CO)_16_{P­(OPh)_3_}_2_] was obtained upon adding *n*-hexane (50 mL) to the acetone solution (0.296 g, yield 65% based on Pt).

**[PMePh_3_]_2_[Pt_9_(CO)_16_{P­(OPh)_3_}_2_]**. C_90_H_66_O_22_P_4_Pt_9_ (3379.07): calcd. (%): C 31.99, H 1.97; found: C 32.12, H 1.79. FT-IR (acetone, 298 K) ν_CO_: 2017(s), 1831­(m) cm^–1^. ^1^H NMR (acetone, 298 K) δ_H_: 7.93–7.77 (m, CH_aryl_, cation), 7.25–7.03 (m, CH_aryl_, ligand), 3.15 (d, ^2^J_H–P_ 14 Hz, CH_3_, cation) ppm. ^31^P­{^1^H} NMR (acetone, 298 K) δ_P_: 149.6 (^1^J_Pt–P_ 8731 Hz, ^2^J_Pt–P_ 1004 Hz), 22.1 (s, cation) ppm.

### 4.16 X-ray Crystallographic Study

Crystal data and collection details for [PMePh_3_]_2_[Pt_9_(CO)_17_{P­(OPh)_3_}]·CH_3_COCH_3_, [PMePh_3_]_2_[Pt_12_(CO)_22_{P­(OPh)_3_}_2_]·solv, [PMePh_3_]_2_[Pt_12_(CO)_22_{P­(OMe)_3_}_2_], [PMePh_3_]_2_[Pt_15_(CO)_28_{P­(OPh)_3_}_2_]**·**2CH_3_COCH_3_·C_6_H_14_, [PMePh_3_]_2_[Pt_12_(CO)_24_] and [Pt­{P­(OPh)_3_}_3_]·THF are reported in Table S7 in the Supporting Information. The diffraction experiments were carried out on a Bruker APEX II diffractometer equipped with a PHOTON2 detector using Mo–Kα radiation. Data were corrected for Lorentz polarization and absorption effects (empirical absorption correction SADABS).^73^ Structures were solved by direct methods and refined by full-matrix least-squares based on all data using *F* ^2^.^74^ Hydrogen atoms were fixed at calculated positions and refined by a riding model. All non-hydrogen atoms were refined with anisotropic displacement parameters, unless otherwise stated. Further details can be found in the Supporting Information, together with ORTEP representations of all the structures (Figures S54–S59 in the Supporting Information).

### 4.17 Computational Simulations

Geometry optimizations and relaxed scans were carried out in the PBEh-3c method,^75^ which is a reparametrized version of PBE (with 42% HF exchange) that uses a split-valence double-ζ basis set (def2-mSVP) with relativistic effective core potentials on Pt.^76–78^ The method adds three corrections considering dispersion, basis set superposition, and other basis set incompleteness effects.^79–81^ Selected PBEh-3c calculations were carried out in combination with the C-PCM solvation model, considering acetone as continuous medium.^82^ Further optimizations were performed the hybrid meta-GGA DFT functional TPSS0, with 25% HF exchange,^83^ in combination with Ahlrichs’ def2-TZVP basis set and relativistic ECP for platinum.^76–78^ The software used was ORCA version 6.1.0.^84,85^ Cartesian coordinates of the DFT-optimized structures are collected in a supplementary xyz file.

## Supplementary Material







## Notes

DFT-optimized coordinates in XYZ format (XYZ)
Molecular structure of a platinum carbonyl cluster with phosphite ligands (GIF)
Supplementary experimental and computational figures and tables; crystal data and collection details; computational input files (PDF)
