# Supplementary figures and images for: Selective Functionalization with Organophosphite Ligands of Atomically Precise Platinum Chini Clusters
Source: Inorg Chem. 2026 May 20;65(22):12661–77. doi: 10.1021/acs.inorgchem.6c01632 (PMC13250994; doi:10.1021/acs.inorgchem.6c01632)

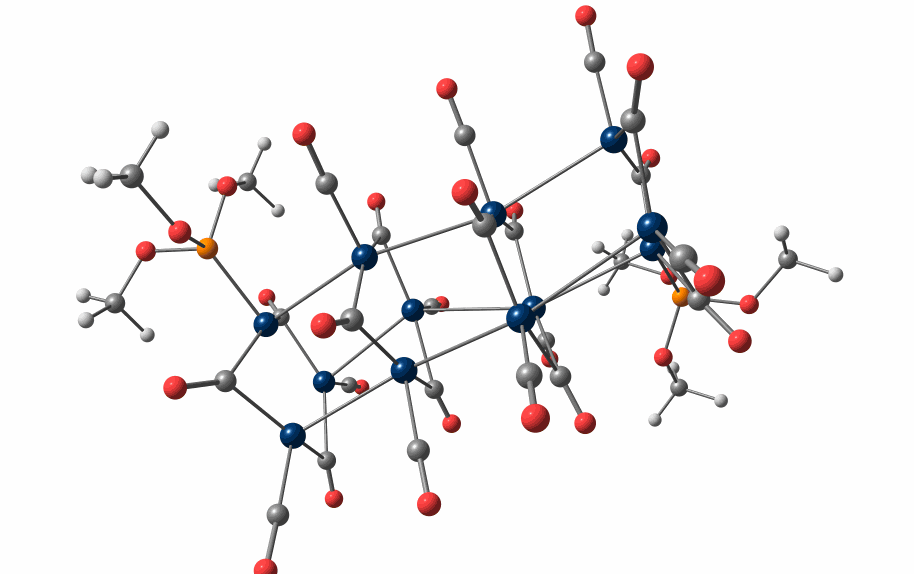

Supplement: Supplementary file 2 [file ic6c01632_si_002.gif]
