# Supplementary material for: Selective Functionalization with Organophosphite Ligands of Atomically Precise Platinum Chini Clusters
Source: Inorg Chem. 2026 May 20;65(22):12661–77. doi: 10.1021/acs.inorgchem.6c01632 (PMC13250994; doi:10.1021/acs.inorgchem.6c01632)
Supplement: Supplementary file 3 [file ic6c01632_si_003.pdf]

Supporting Information for

Selective Functionalization with Organophosphite Ligands of Atomically Precise Platinum Chini Clusters

Francesca Forti,[a] Cristiana Cesari,[a] Marco Bortoluzzi,[b] Cristina Femoni,[a] Maria Carmela Iapalucci,[a] and Stefano Zacchini[a]*

[a] Dipartimento di Chimica Industriale "Toso Montanari", Università di Bologna, Via P. Gobetti 85 - 40129 Bologna. Italy. E-mail: stefano.zacchini@unibo.it

[b] Dipartimento di Scienze Molecolari e Nanosistemi, Ca' Foscari University of Venice, Via Torino 155 – 30175 Mestre (Ve), Italy.

	Page/s
IR spectra	S1-S10
NMR spectra	S11-S25
ESI-MS	S26-S31
Supplementary SC-XRD figures and tables	S32-S39
Supplementary computational figures and tables	S40-S43
References	S44

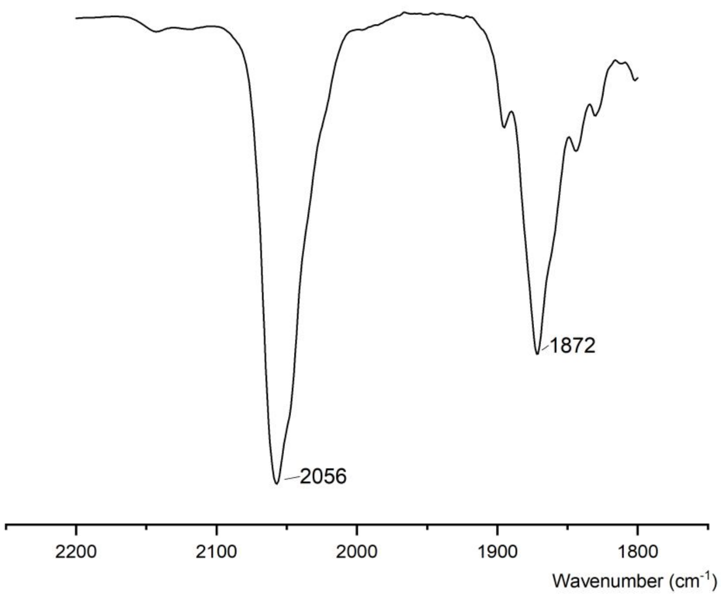

Figure S1. ν_{CO} region of the FT-IR spectrum of $[Pt_{15}(CO)_{30}]^{2-}$ in acetone.

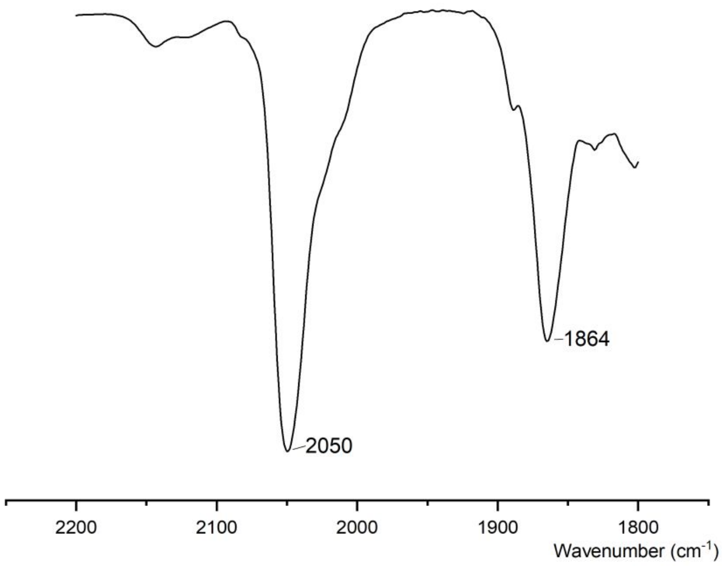

Figure S2. ν_{CO} region of the FT-IR spectrum of $[Pt_{15}(CO)_{29}\{P(OPh)_3\}]^{2-}$ in acetone.

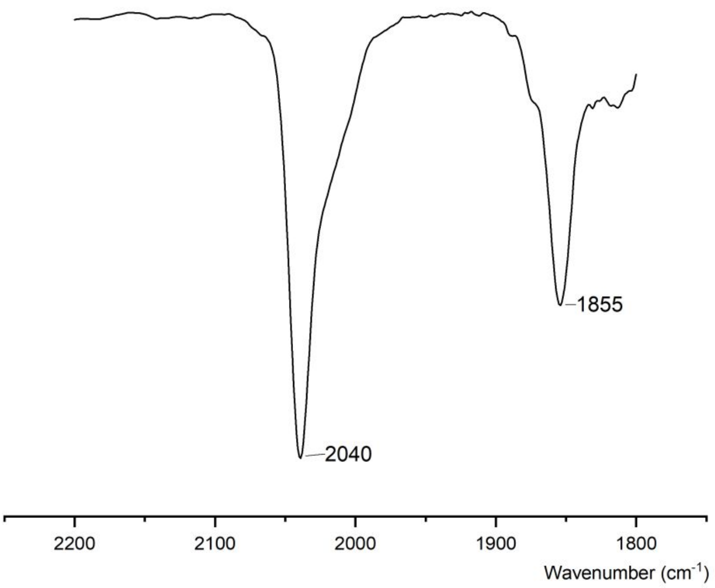

Figure S3. ν_{CO} region of the FT-IR spectrum of $[Pt_{15}(CO)_{28}\{P(OPh)_3\}_2]^{2-}$ in acetone.

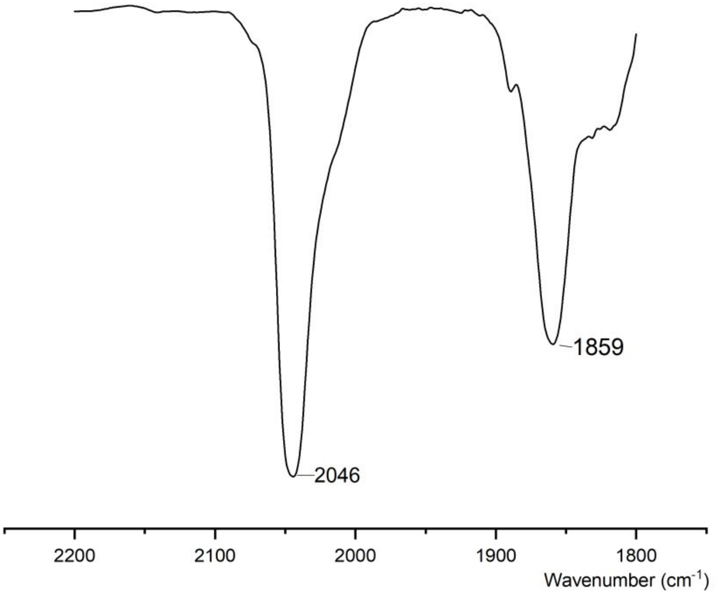

Figure S4. ν_{CO} region of the FT-IR spectrum of $[Pt_{12}(CO)_{24}]^{2-}$ in acetone.

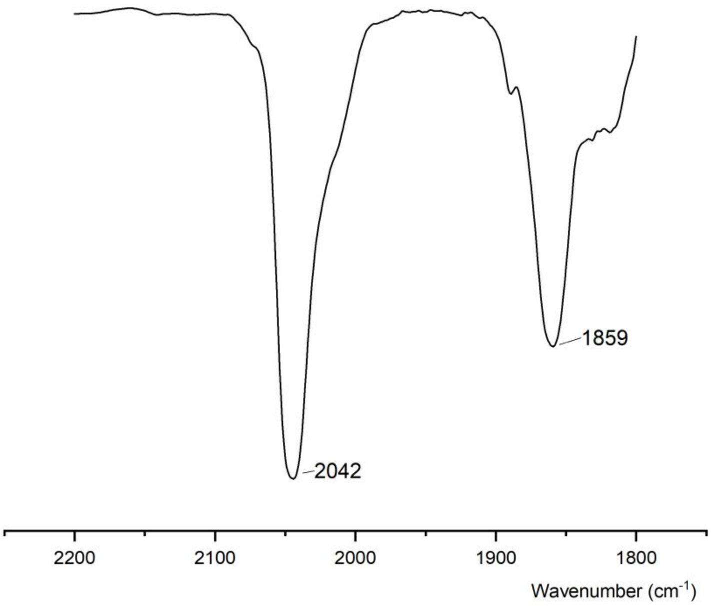

Figure S5. ν_{CO} region of the FT-IR spectrum of $[Pt_{12}(CO)_{23}\{P(OPh)_3\}]^{2-}$ in acetone.

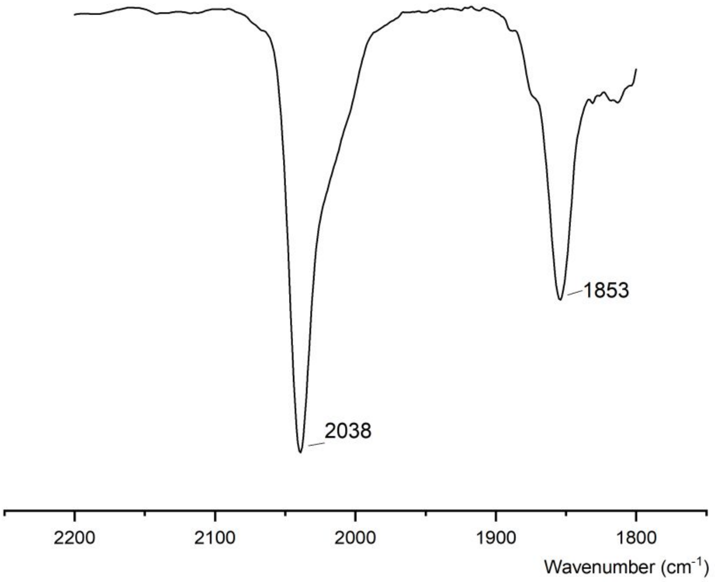

Figure S6. ν_{CO} region of the FT-IR spectrum of $[Pt_{12}(CO)_{22}\{P(OMe)_3\}_2]^{2-}$ in acetone.

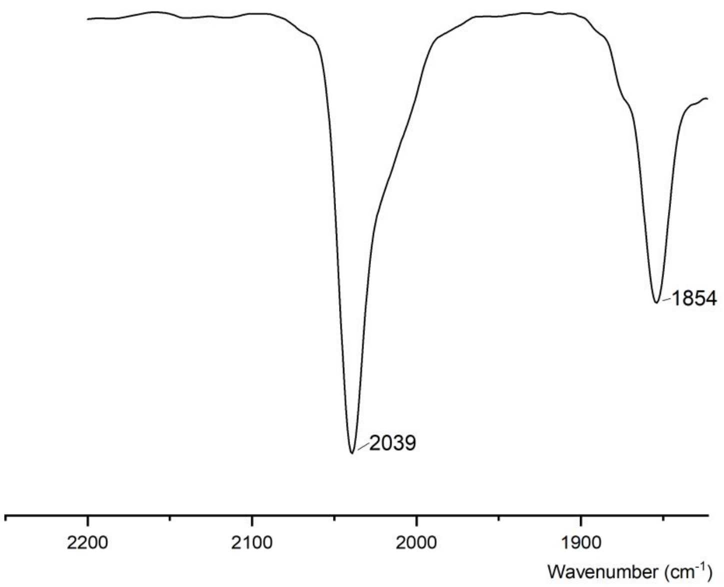

Figure S7. ν_{CO} region of the FT-IR spectrum of $[Pt_{12}(CO)_{22}\{P(OEt)_3\}_2]^{2-}$ in acetone.

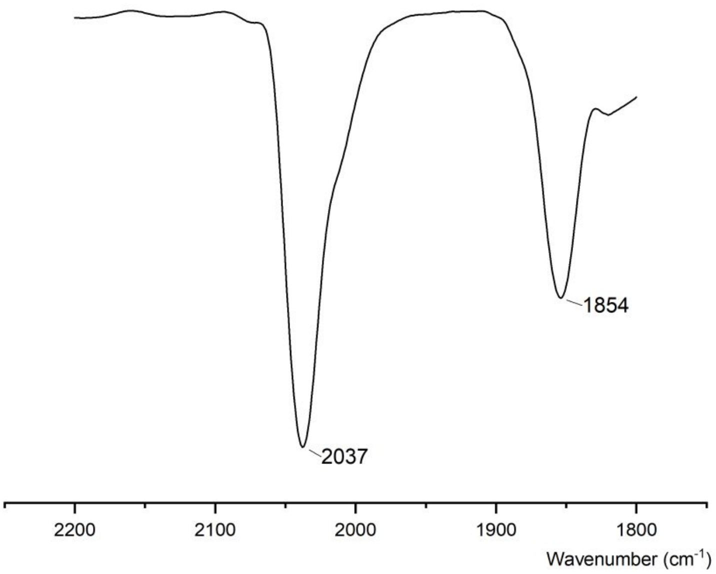

Figure S8. ν_{CO} region of the FT-IR spectrum of $[Pt_{12}(CO)_{22}\{P(OPh)_3\}_2]^{2-}$ in acetone.

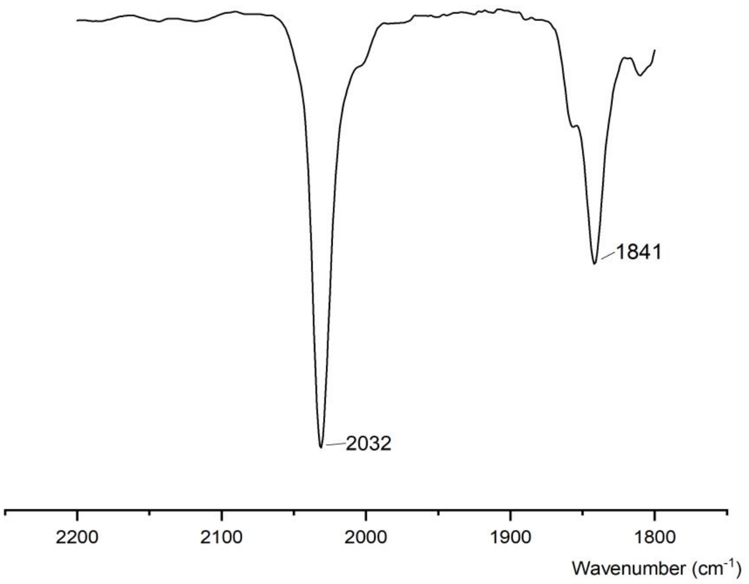

Figure S9. ν_{CO} region of the FT-IR spectrum of $[Pt_9(CO)_{18}]^{2-}$ in acetone.

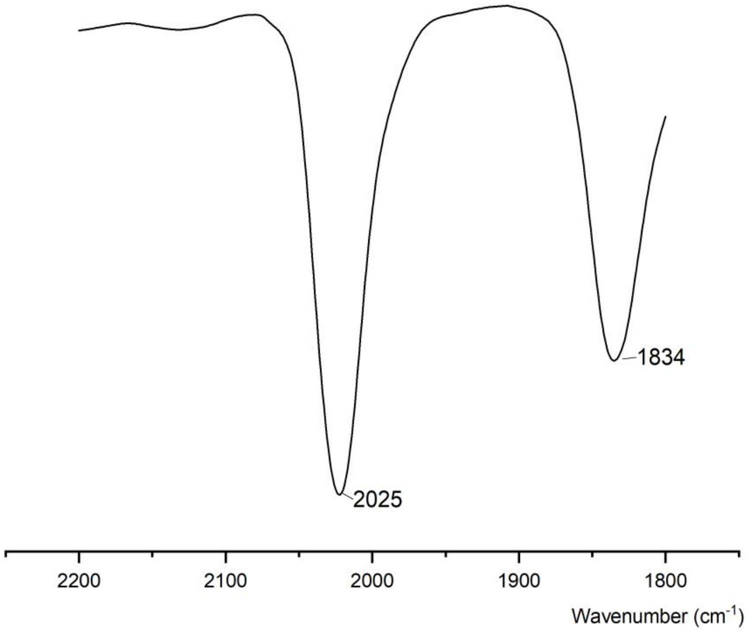

Figure S10. ν_{CO} region of the FT-IR spectrum of $[Pt_9(CO)_{17}\{P(OMe)_3\}]^{2-}$ in acetone.

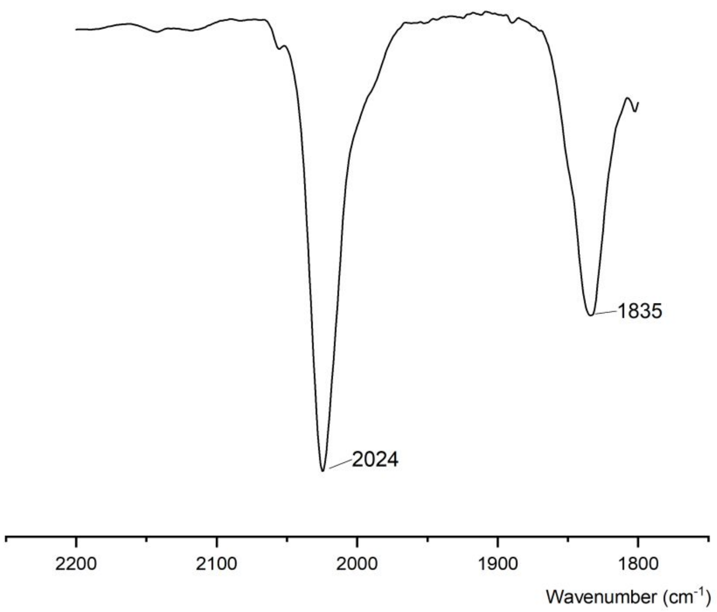

Figure S11. ν_{CO} region of the FT-IR spectrum of $[Pt_9(CO)_{17}\{P(OEt)_3\}]^{2-}$ in acetone.

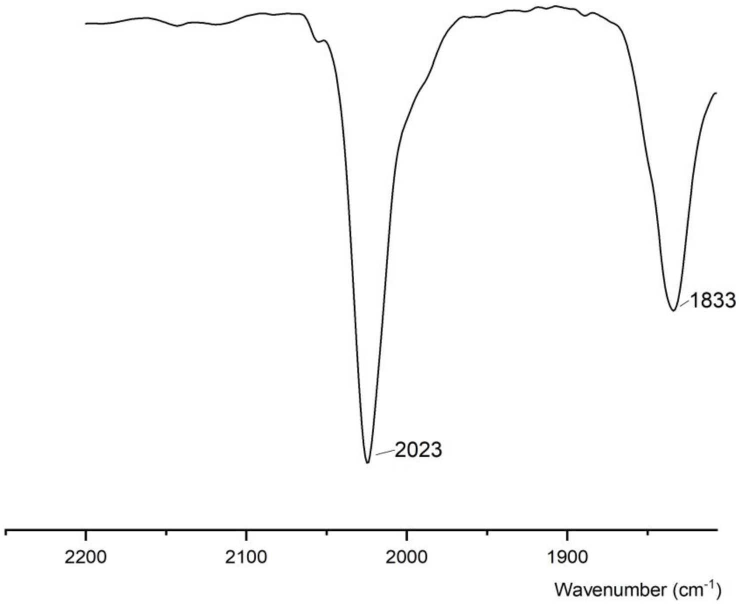

Figure S12. ν_{CO} region of the FT-IR spectrum of $[Pt_9(CO)_{17}\{P(OPh)_3\}]^{2-}$ in acetone.

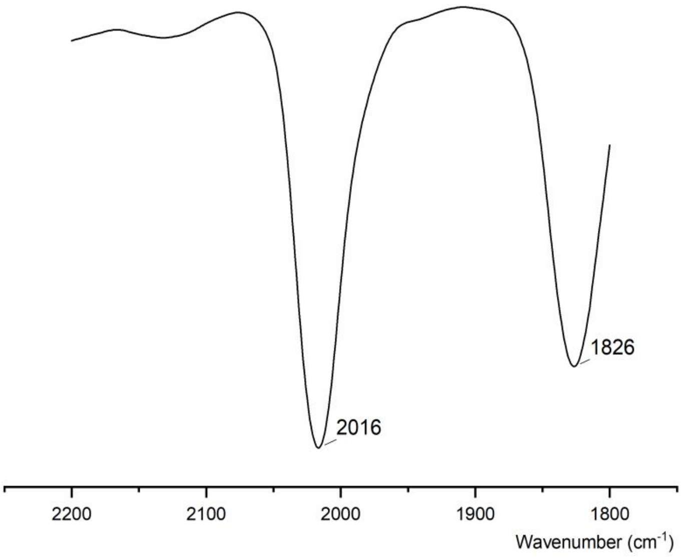

Figure S13. ν_{CO} region of the FT-IR spectrum of $[Pt_9(CO)_{16}\{P(OMe)_3\}_2]^{2-}$ in acetone.

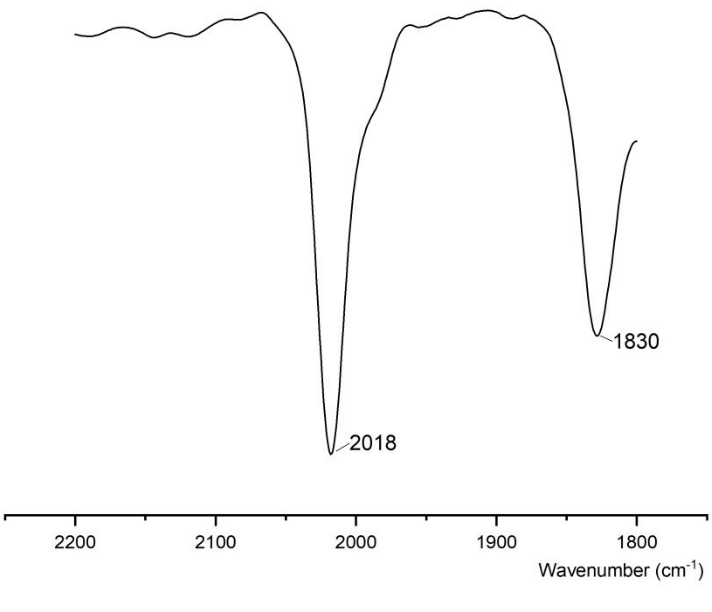

Figure S14. ν_{CO} region of the FT-IR spectrum of $[Pt_9(CO)_{16}\{P(OEt)_3\}_2]^{2-}$ in acetone.

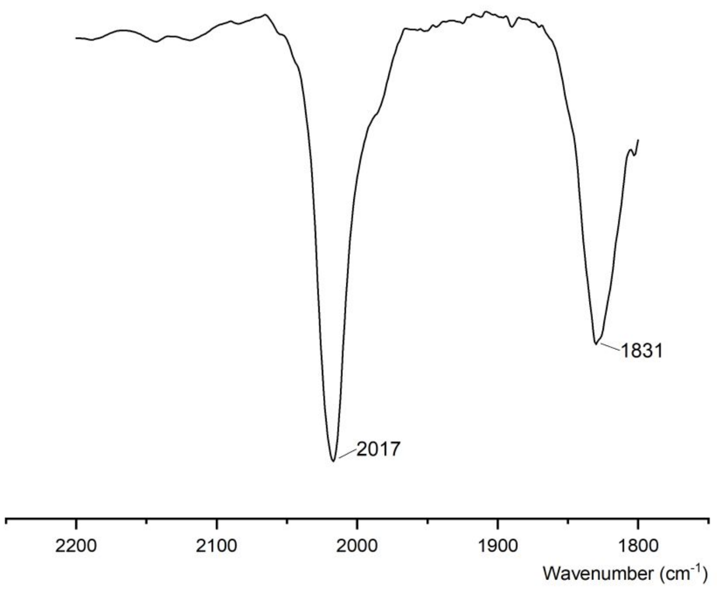

Figure S15. ν_{CO} region of the FT-IR spectrum of $[Pt_9(CO)_{16}\{P(OPh)_3\}_2]^{2-}$ in acetone.

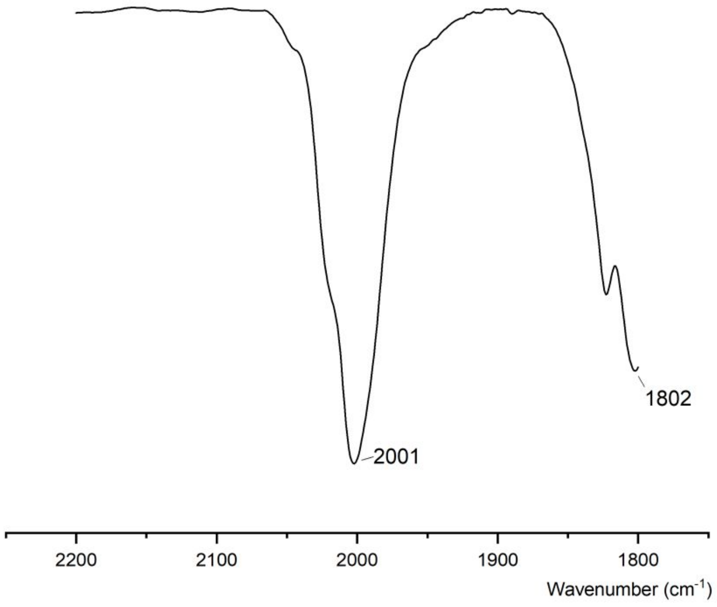

Figure S16. ν_{CO} region of the FT-IR spectrum of $[Pt_6(CO)_{12}]^{2-}$ in acetone.

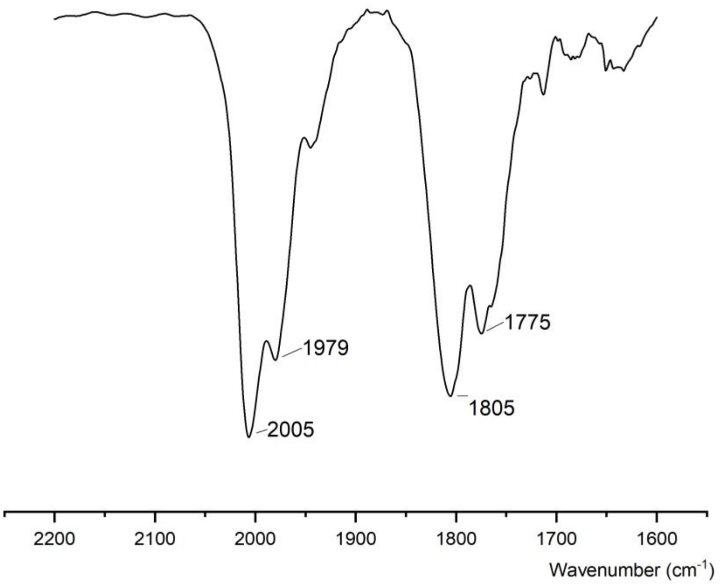

Figure S17. ν_{CO} region of the FT-IR spectrum of the reaction between $[Pt_6(CO)_{12}]^{2-}$ and $P(OMe)_3$ in acetonitrile.

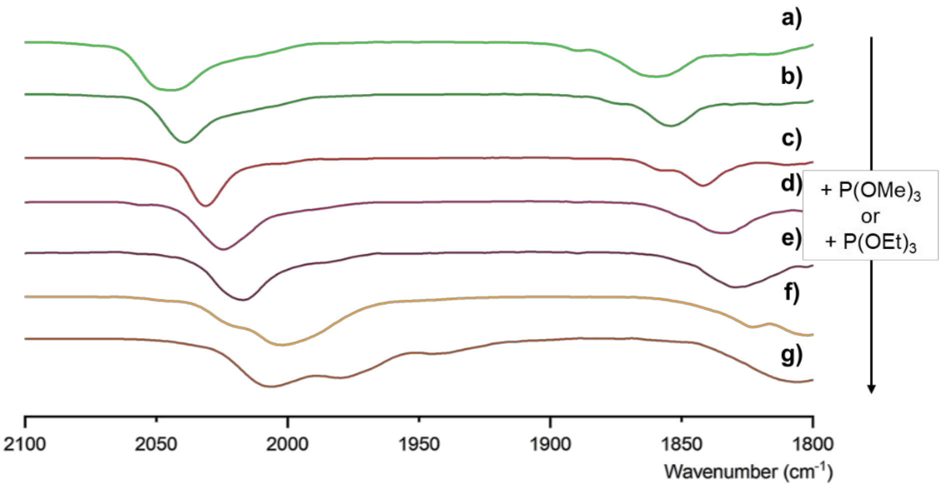

Figure S18. FT-IR spectra in the ν_{CO} region, recorded at room temperature in acetone solution, obtained upon the stepwise addition of $P(OMe)_3$ or $P(OEt)_3$ to $[Pt_{12}(CO)_{24}]^{2-}$. **a)** $[Pt_{12}(CO)_{24}]^{2-}$, **b)** $[Pt_{12}(CO)_{22}\{P(OR)_3\}_2]^{2-}$, **c)** $[Pt_9(CO)_{18}]^{2-}$, **d)** $[Pt_9(CO)_{17}\{P(OR)_3\}]^{2-}$, **e)** $[Pt_9(CO)_{16}\{P(OR)_3\}_2]^{2-}$, **f)** $[Pt_6(CO)_{12}]^{2-}$, **g)** $[Pt_6(CO)_{11}\{P(OR)_3\}]^{2-}$.

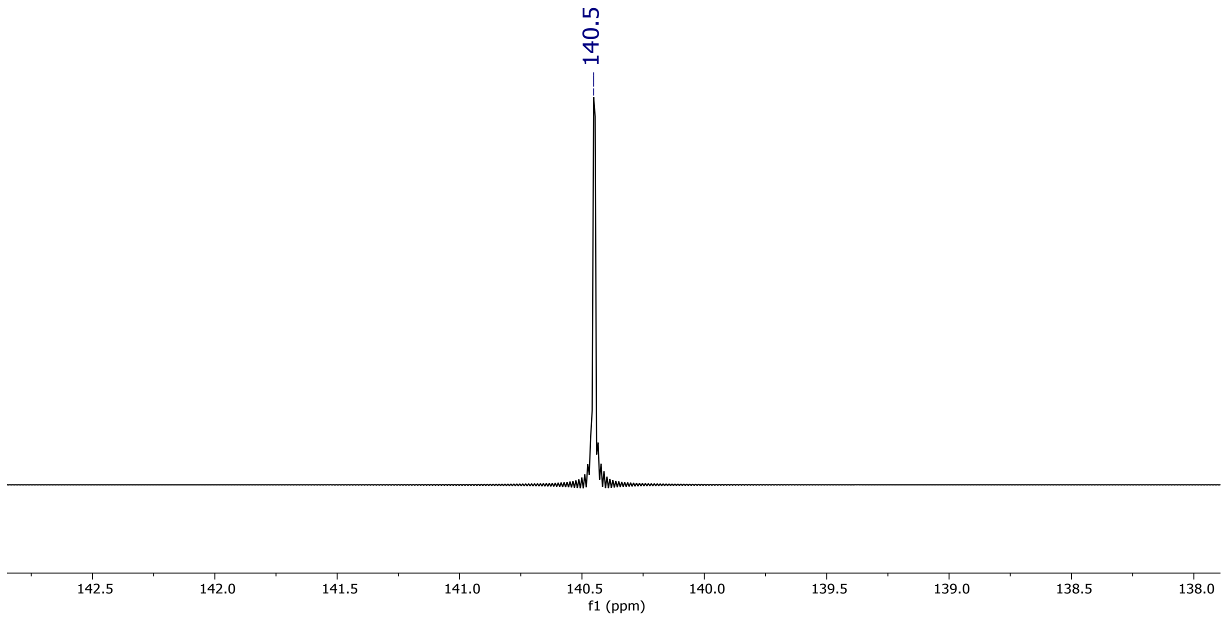

Figure S19. $^{31}P\{^1H\}$ NMR spectrum of $P(OMe)_3$ in CD_3Cl at 298 K.

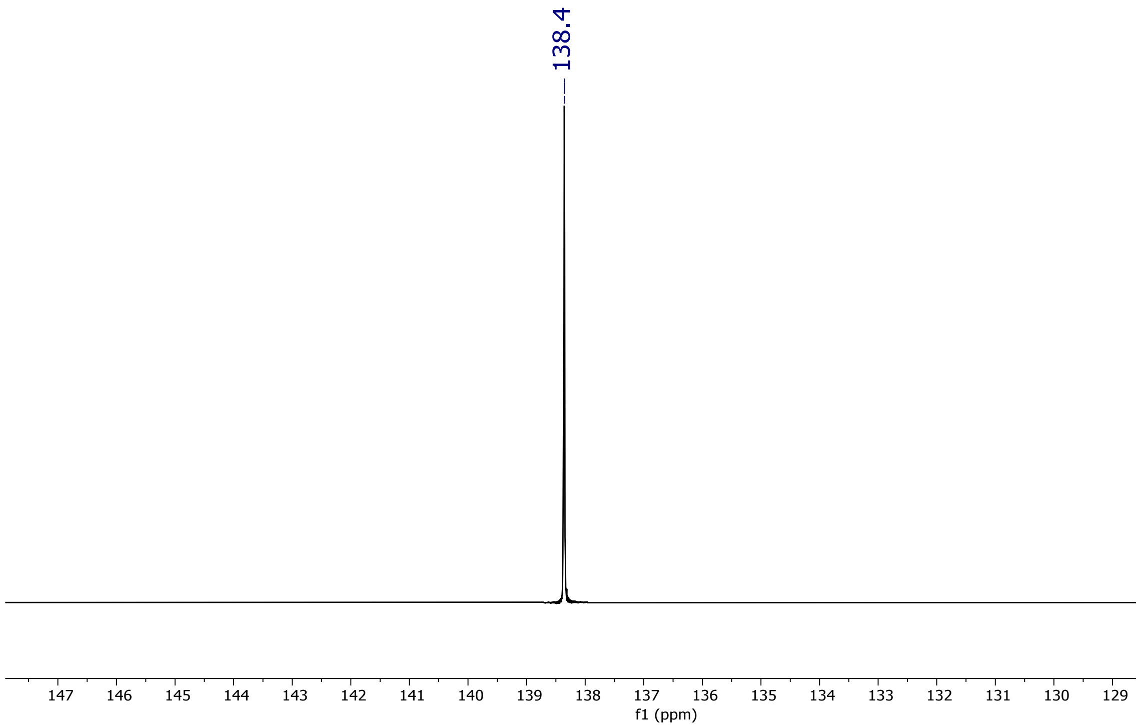

Figure S20. $^{31}P\{^1H\}$ NMR spectrum of $P(OEt)_3$ in CD_3Cl at 298 K.

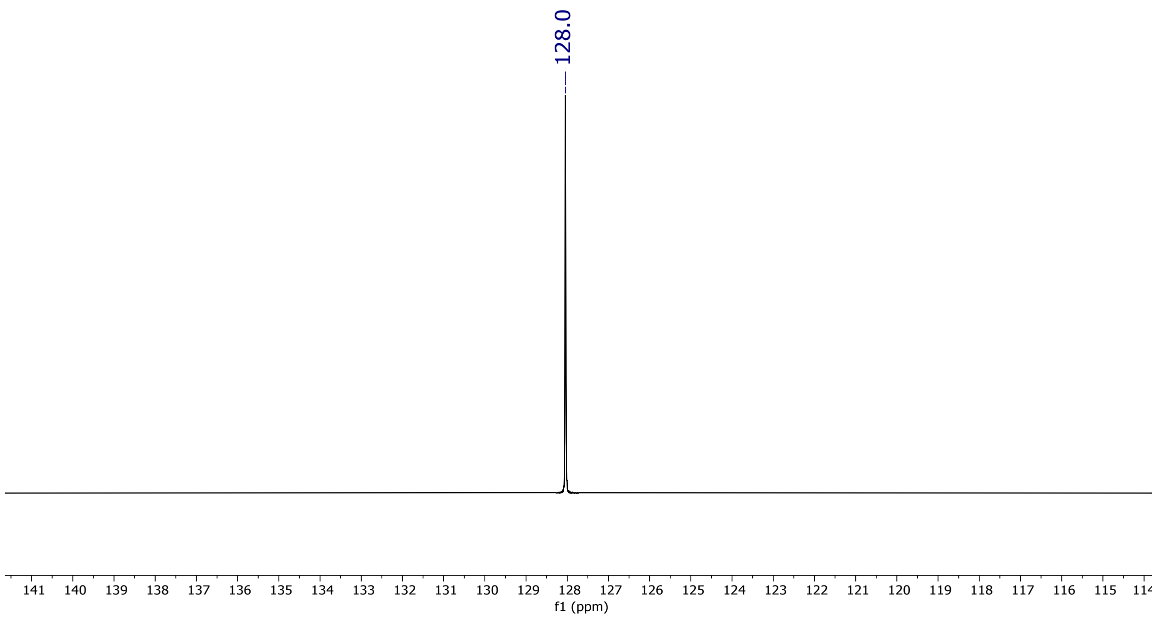

Figure S21. $^{31}P\{^1H\}$ NMR spectrum of $P(OPh)_3$ in CD_3Cl at 298 K.

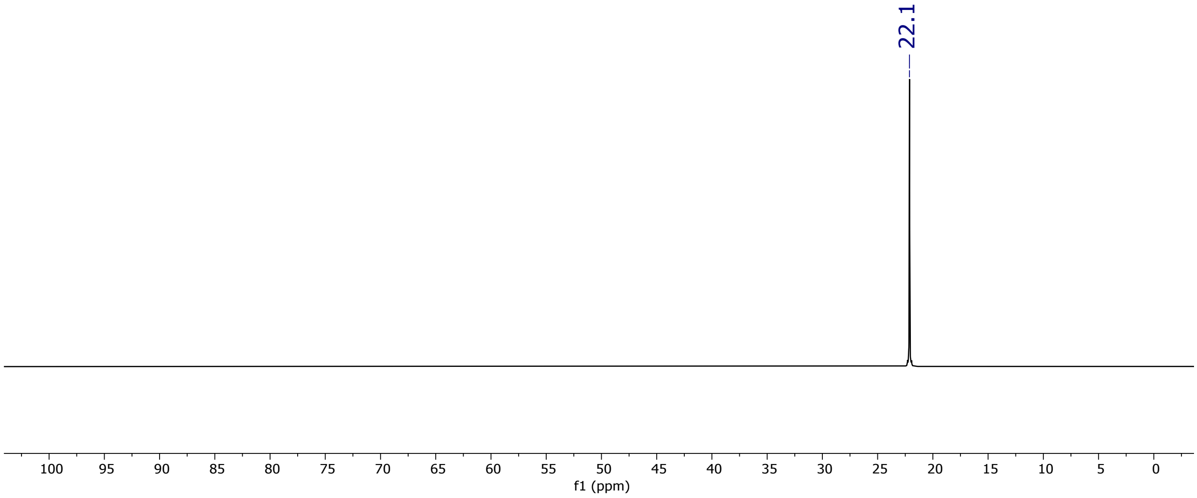

Figure S22. $^{31}P\{^1H\}$ NMR spectrum in acetone-d_6 at 298 K of $[PMePh_3]_2[Pt_{15}(CO)_{29}\{P(OPh)_3\}]$ showing the singlet of the $[PMePh_3]^+$ cation. This resonance is present in all the spectra where this cation has been employed as counterion, but in Figures S23-S28 and S30-S32 only the region corresponding to the $P(OR)_3$ ligands bonded to the cluster anion has been reported for sake of clarity.

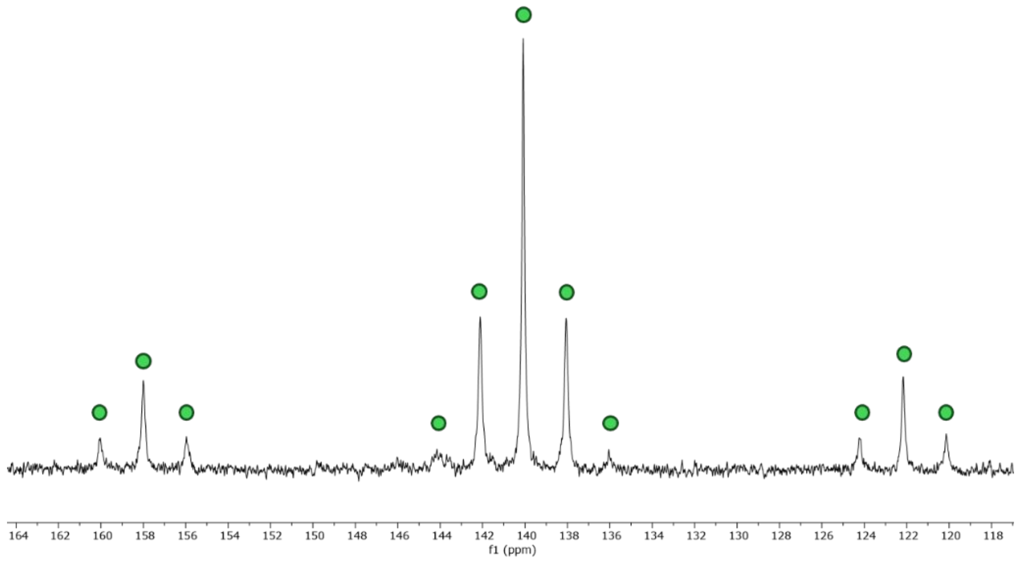

Figure S23. $^{31}P\{^{1}H\}$ NMR spectrum of $[PMePh_3]_2[Pt_{15}(CO)_{29}\{P(OPh)_3\}]$ in acetone-d_6 at 298 K.

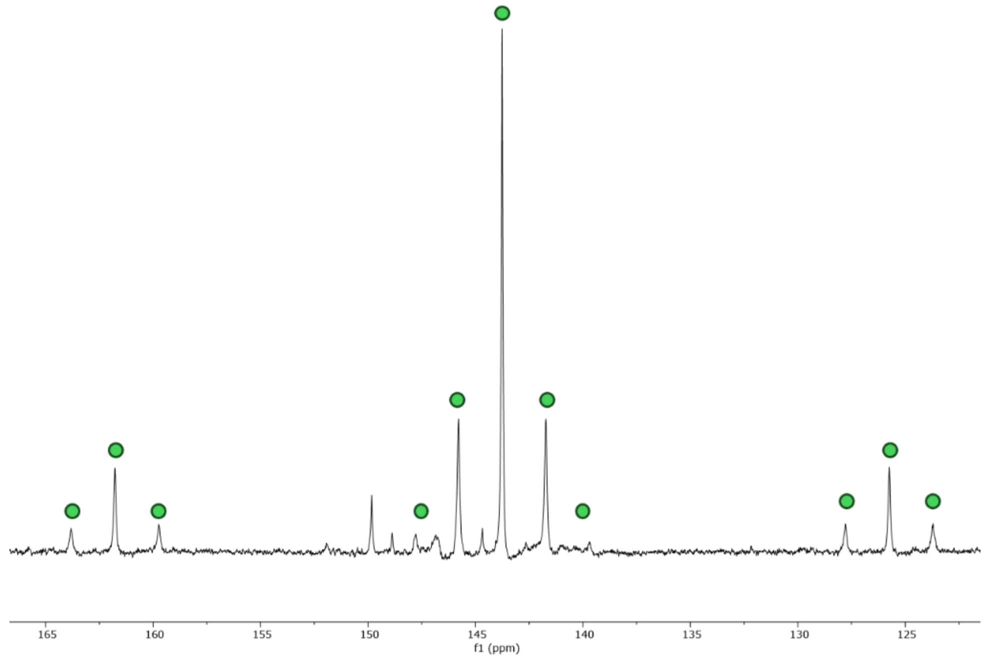

Figure S24. $^{31}P\{^{1}H\}$ NMR spectrum of $[PMePh_3]_2[Pt_{15}(CO)_{28}\{P(OPh)_3\}_2]$ in acetone-d_6 at 298 K.

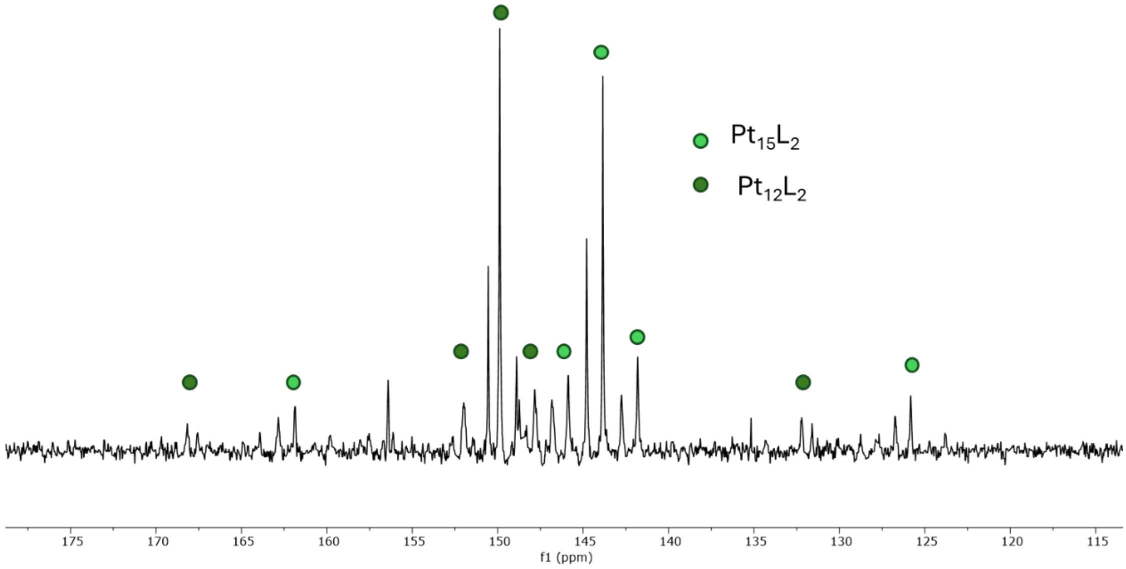

Figure S25. $^{31}P\{^{1}H\}$ NMR spectrum of a mixture of $[PMePh_3]_2[Pt_{12}(CO)_{22}\{P(OPh)_3\}_2]$ and $[PMePh_3]_2[Pt_{15}(CO)_{28}\{P(OPh)_3\}_2]$ in acetone-d$_6$ at 298 K.

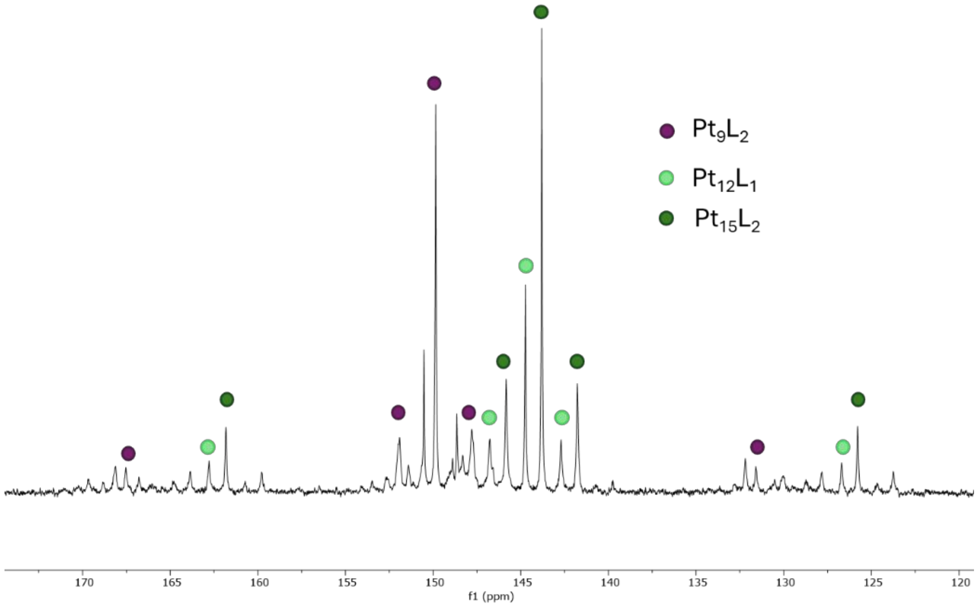

Figure S26. $^{31}P\{^{1}H\}$ NMR spectrum of a mixture of $[PMePh_3]_2[Pt_9(CO)_{16}\{P(OPh)_3\}_2]$, $[PMePh_3]_2[Pt_{12}(CO)_{23}\{P(OPh)_3\}]$ and $[PMePh_3]_2[Pt_{15}(CO)_{28}\{P(OPh)_3\}_2]$ in acetone-d$_6$ at 298 K.

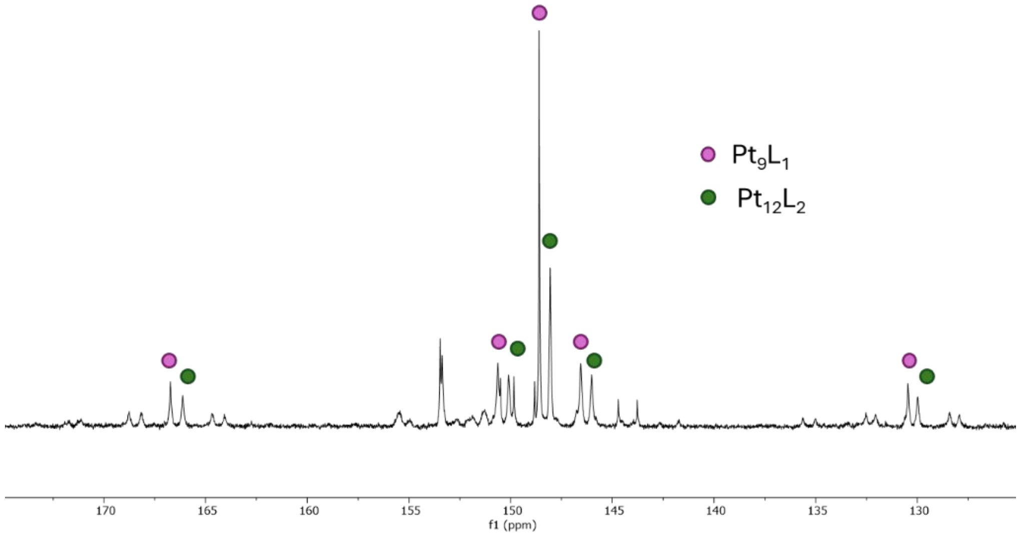

Figure S27. $^{31}P\{^{1}H\}$ NMR spectrum of a mixture of $[PMePh_3]_2[Pt_9(CO)_{17}\{P(OPh)_3\}]$ and $[PMePh_3]_2[Pt_{12}(CO)_{22}\{P(OPh)_3\}_2]$ in acetone-d_6 at 298 K.

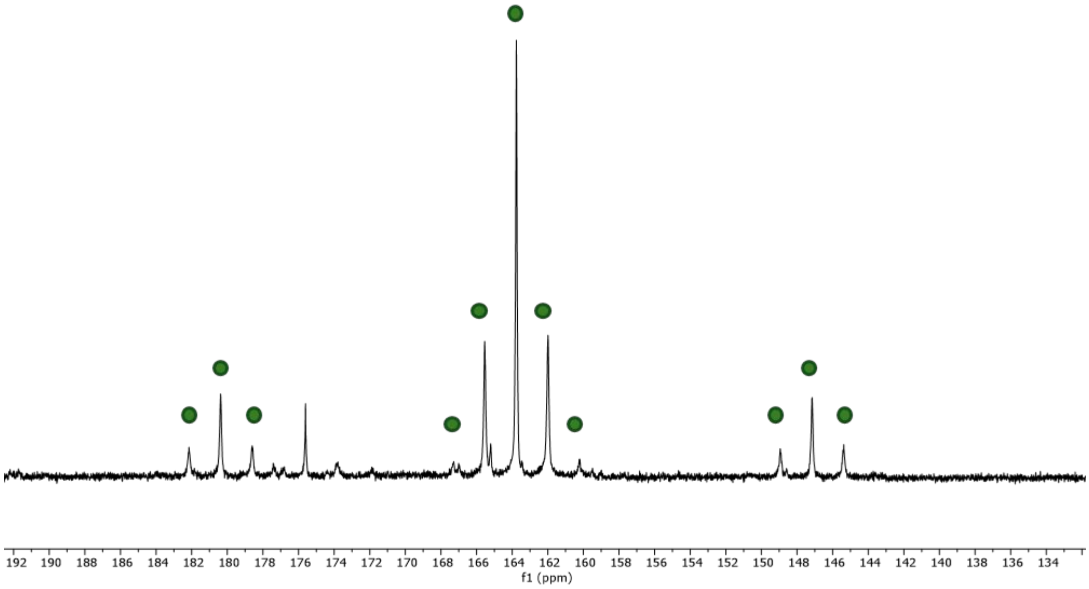

Figure S28. $^{31}P\{^{1}H\}$ NMR spectrum of $[PMePh_3]_2[Pt_{12}(CO)_{22}\{P(OMe)_3\}_2]$ in acetone-d_6 at 298 K.

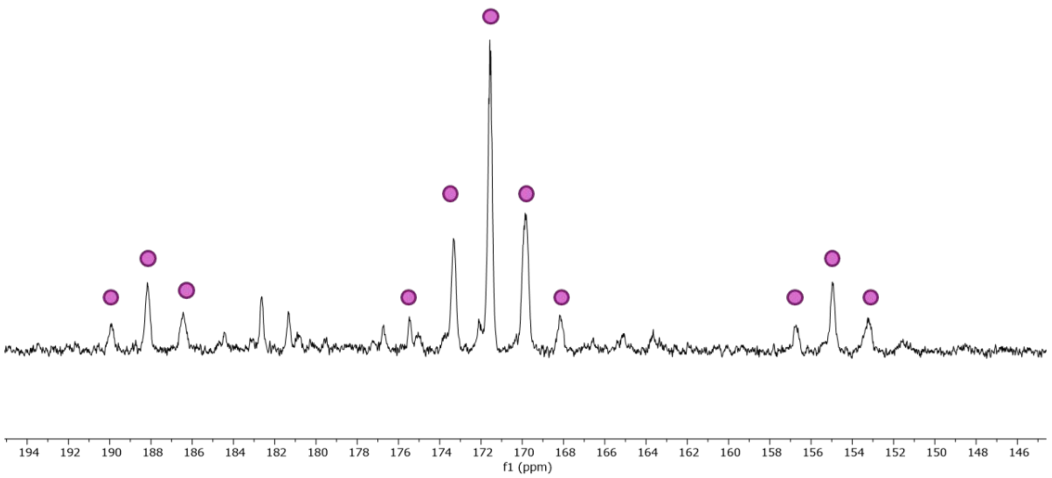

Figure S29. $^{31}P\{^1H\}$ NMR spectrum of $[NEt_4]_2[Pt_9(CO)_{16}\{P(OMe)_3\}_2]$ in acetone-d_6 at 298 K.

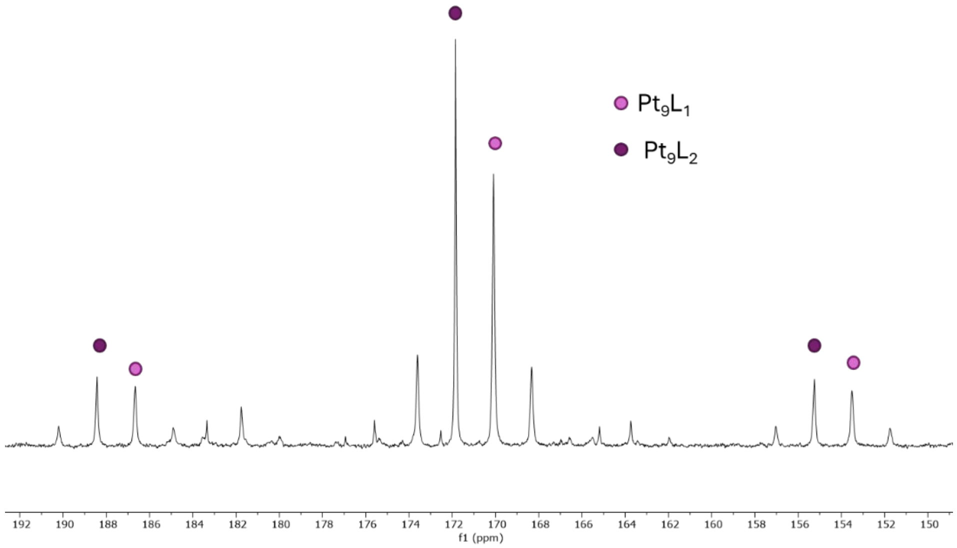

Figure S30. $^{31}P\{^1H\}$ NMR spectrum of a mixture of $[PMePh_3]_2[Pt_9(CO)_{17}\{P(OMe)_3\}]$ and $[PMePh_3]_2[Pt_9(CO)_{16}\{P(OMe)_3\}_2]$ in acetone-d_6 at 298 K.

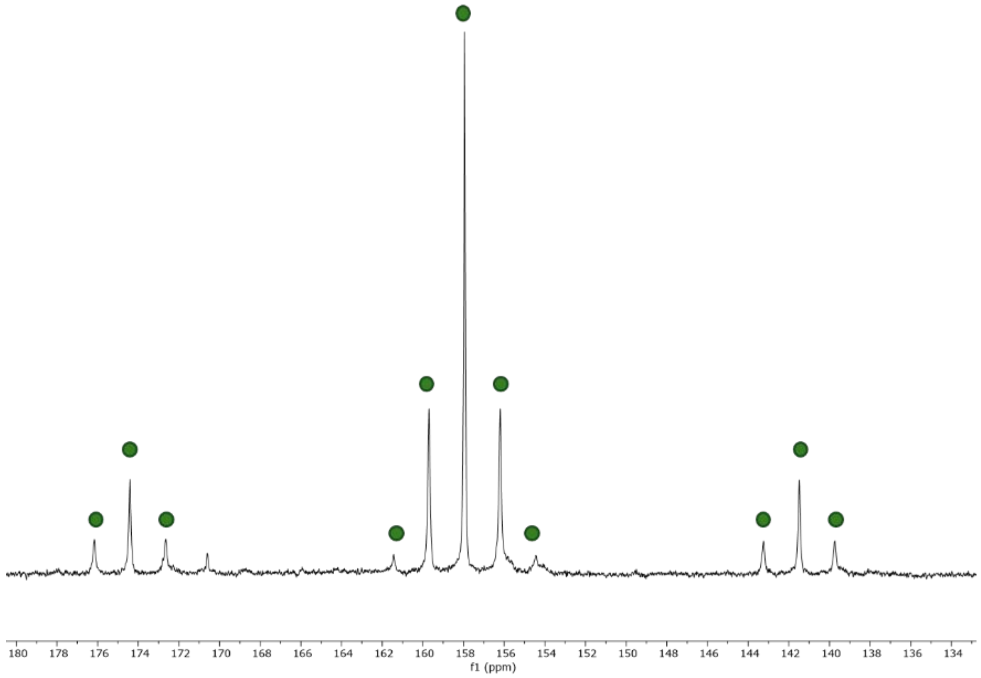

Figure S31. $^{31}P\{^1H\}$ NMR spectrum of $[PMePh_3]_2[Pt_{12}(CO)_{22}\{P(OEt)_3\}_2]$ in acetone d_6 at 298 K.

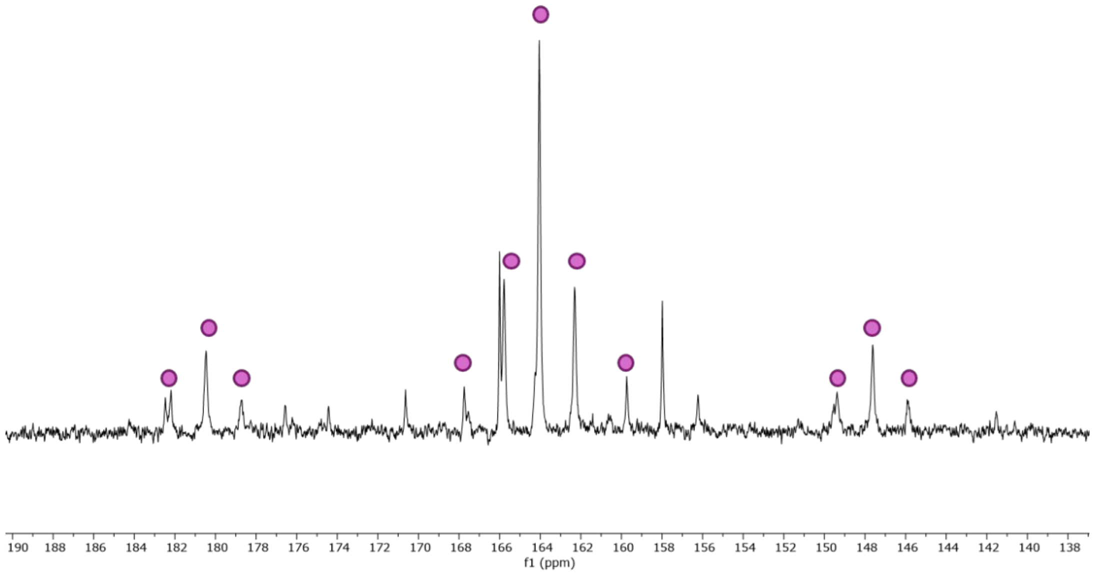

Figure S32. $^{31}P\{^1H\}$ NMR spectrum of $[PMePh_3]_2[Pt_9(CO)_{17}\{P(OEt)_3\}]$ in acetone-d_6 at 298 K.

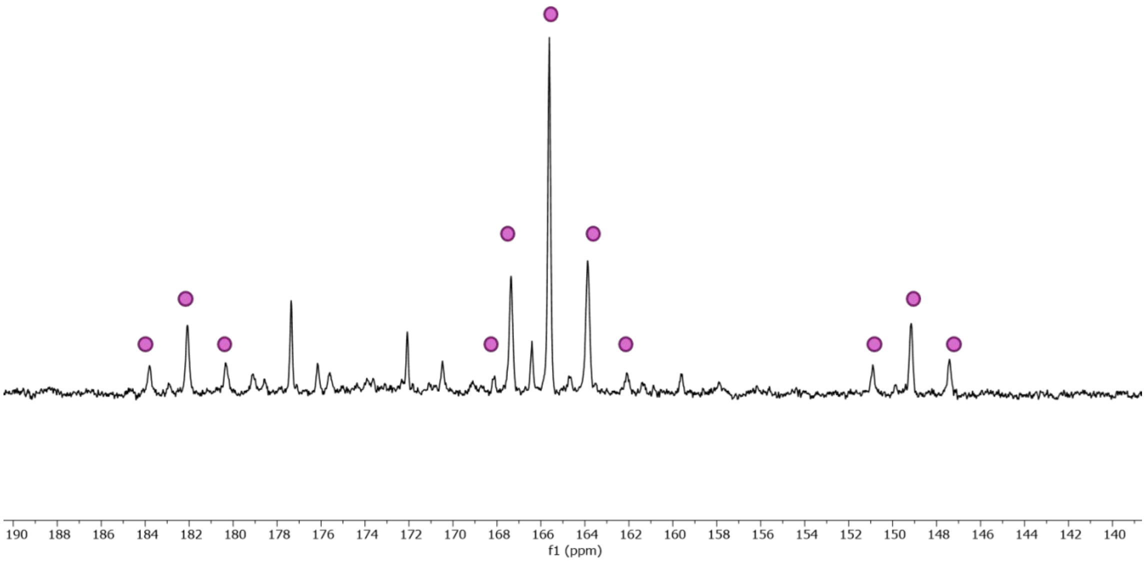

Figure S33. $^{31}P\{^{1}H\}$ NMR spectrum of $[NEt_4]_2[Pt_9(CO)_{16}\{P(OEt)_3\}_2]$ in acetone-d_6 at 298 K.

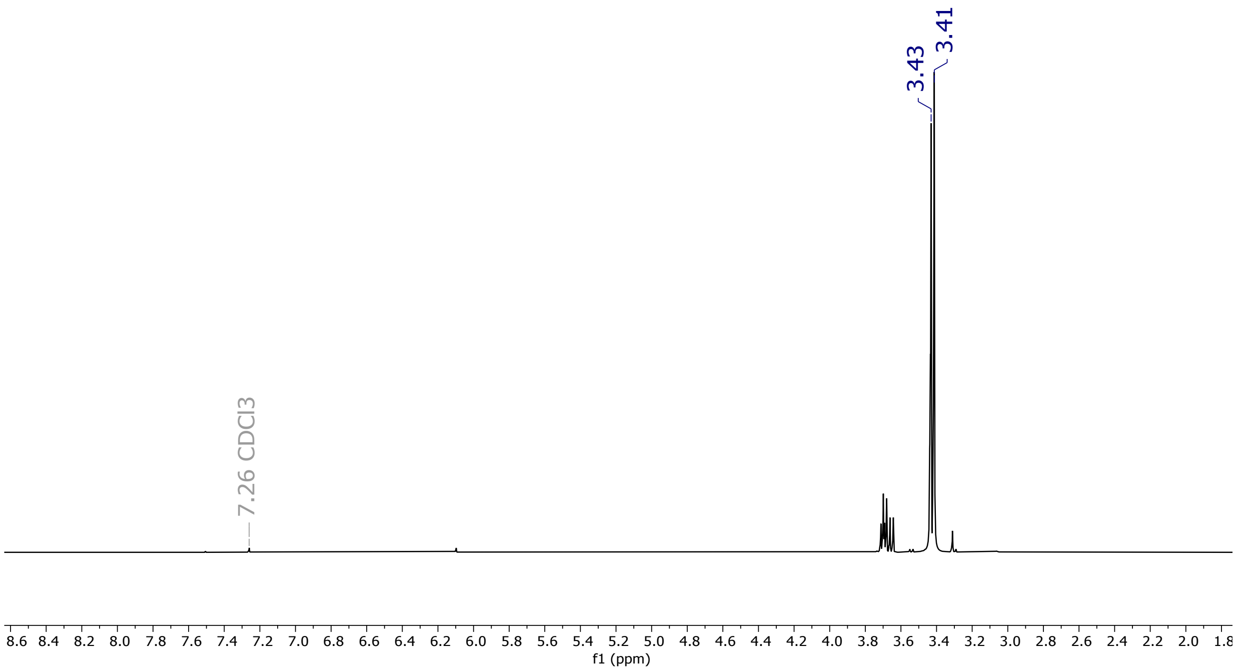

Figure S34. ^{1}H NMR spectrum of $P(OMe)_3$ in CD_3Cl at 298 K.

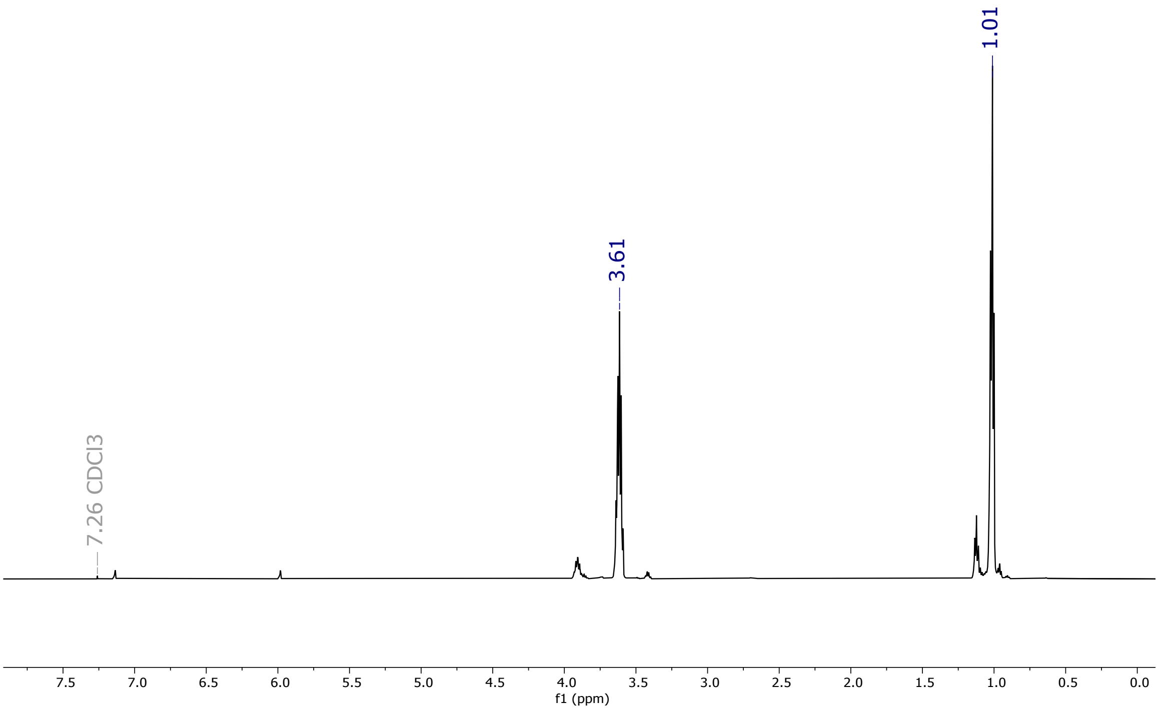

Figure S35. ^{1}H NMR spectrum of $P(OEt)_3$ in CD_3Cl at 298 K.

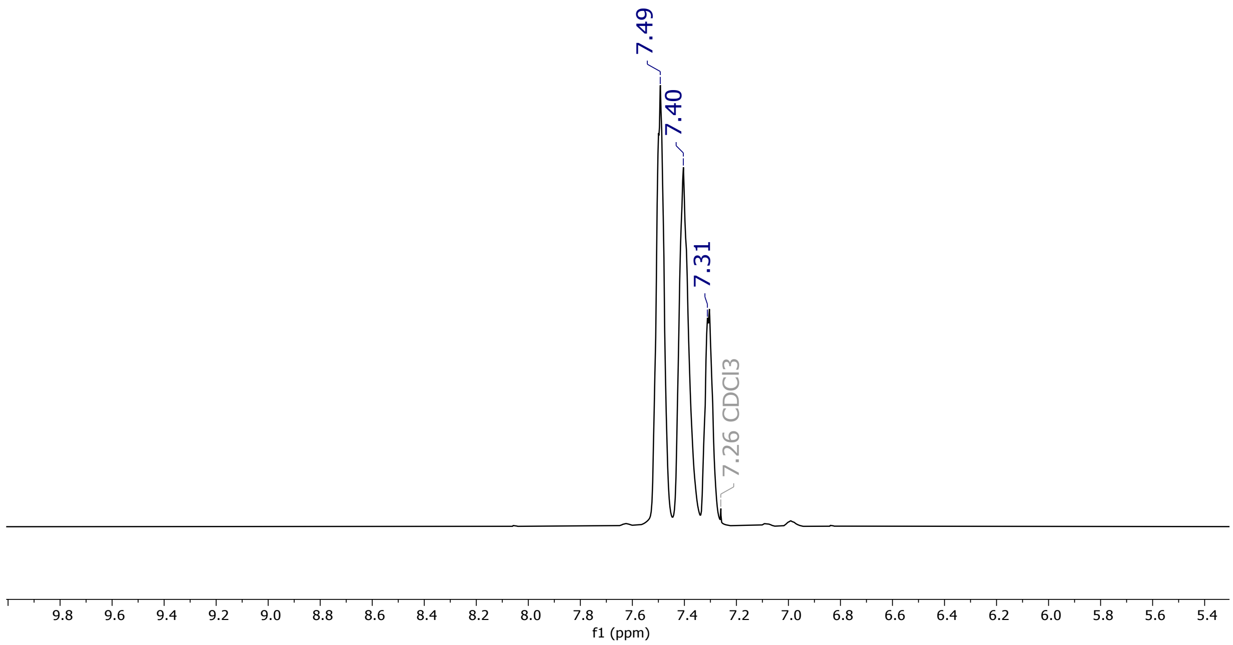

Figure S36. ^{1}H NMR spectrum of $P(OPh)_3$ in CD_3Cl at 298 K.

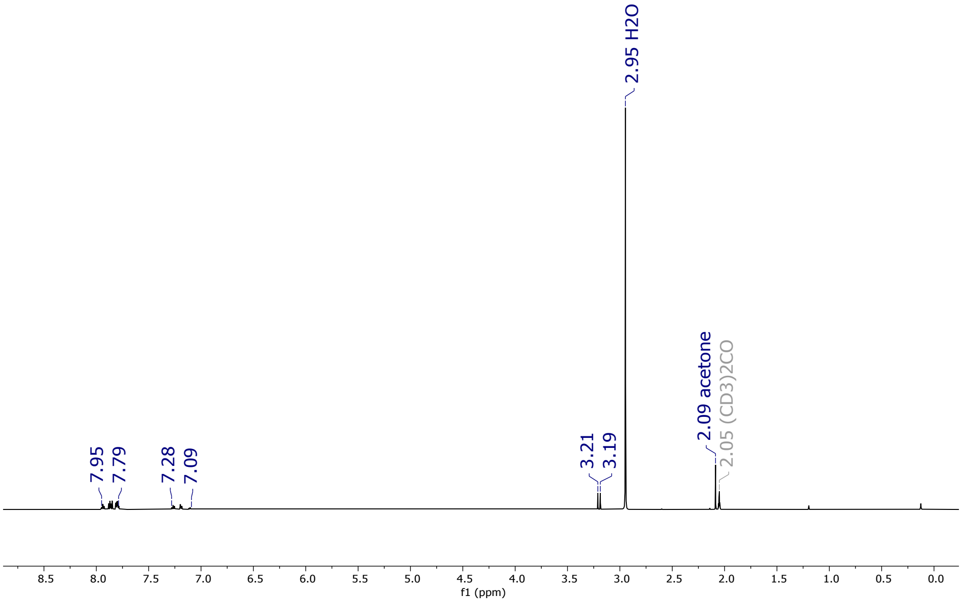

Figure S37. ^{1}H NMR spectrum of $[PMePh_3]_2[Pt_{15}(CO)_{29}\{P(OPh)_3\}]$ in acetone-d_6 at 298 K.

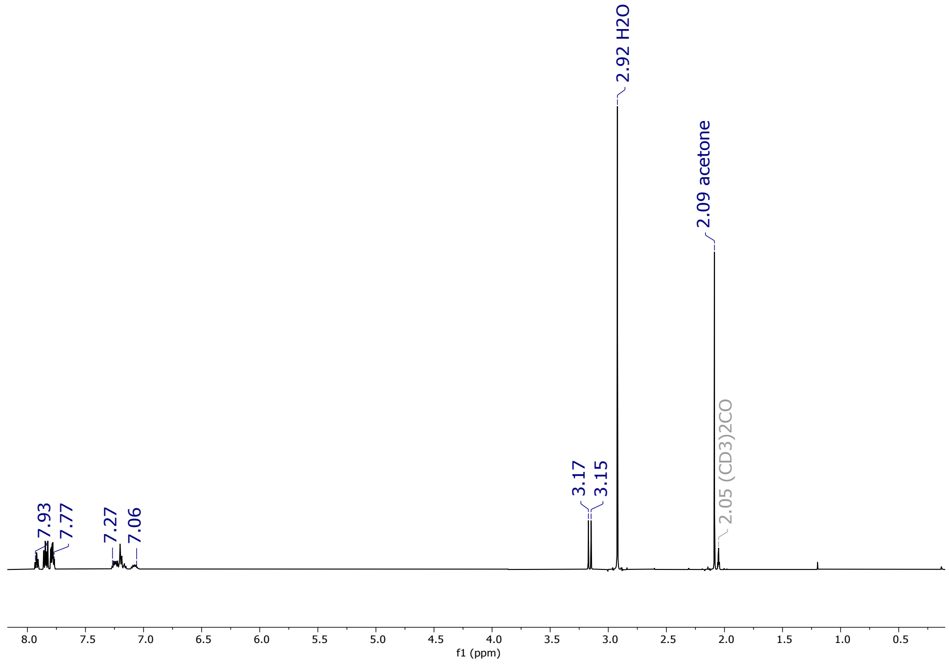

Figure S38. ^{1}H NMR spectrum of $[PMePh_3]_2[Pt_{15}(CO)_{28}\{P(OPh)_3\}_2]$ in acetone-d_6 at 298 K.

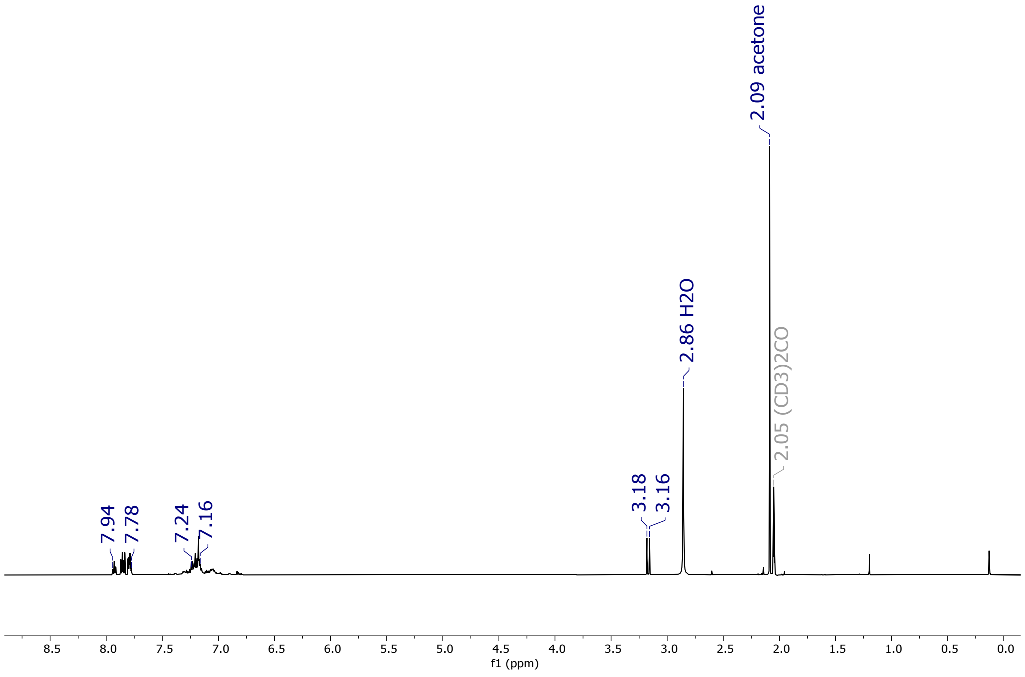

Figure S39. ^{1}H NMR spectrum of a mixture of $[PMePh_3]_2[Pt_{12}(CO)_{22}\{P(OPh)_3\}_2]$ and $[PMePh_3]_2[Pt_{15}(CO)_{28}\{P(OPh)_3\}_2]$ in acetone-d_6 at 298 K.

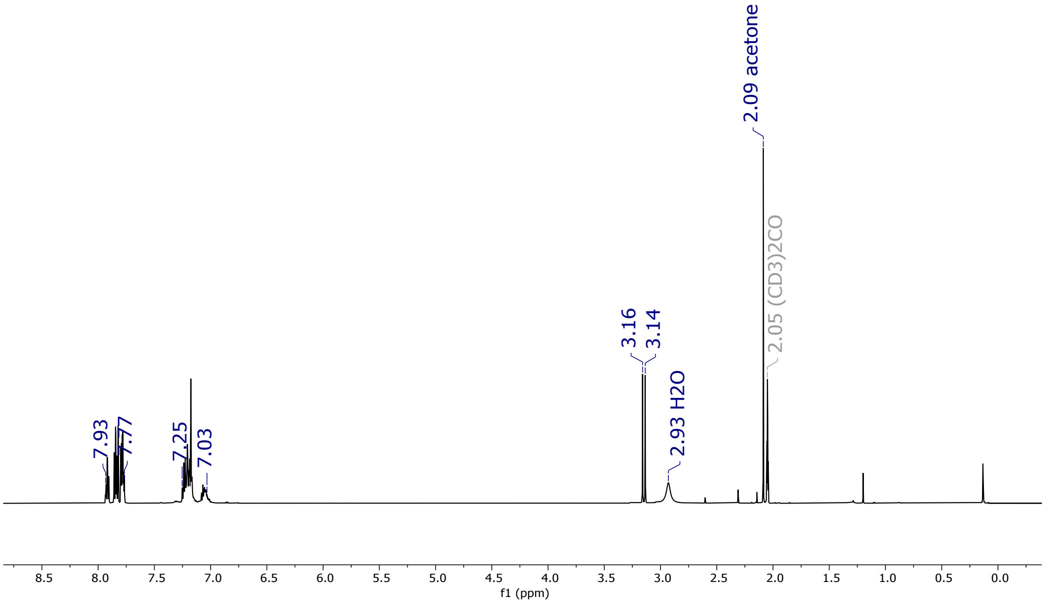

Figure S40. ^{1}H NMR spectrum of a mixture of $[PMePh_3]_2[Pt_9(CO)_{16}\{P(OPh)_3\}_2]$, $[PMePh_3]_2[Pt_{12}(CO)_{23}\{P(OPh)_3\}]$ and $[PMePh_3]_2[Pt_{15}(CO)_{28}\{P(OPh)_3\}_2]$ in acetone-d_6 at 298 K.

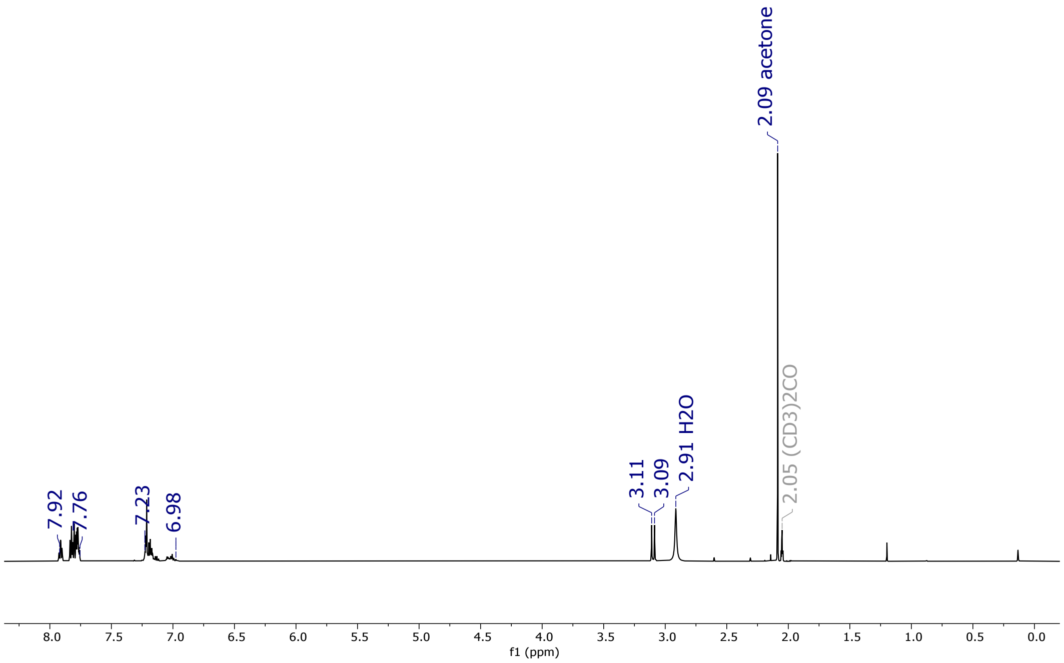

Figure S41. ^{1}H NMR spectrum of a mixture of $[PMePh_3]_2[Pt_9(CO)_{17}\{P(OPh)_3\}]$ and $[PMePh_3]_2[Pt_{12}(CO)_{22}\{P(OPh)_3\}_2]$ in acetone-d_6 at 298 K.

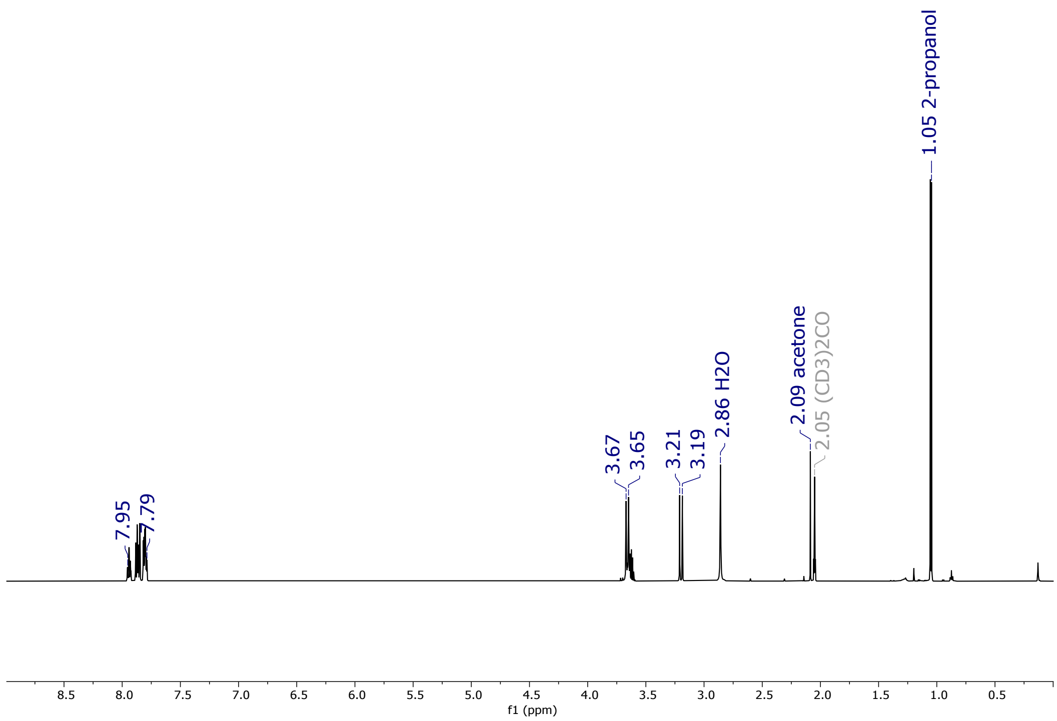

Figure S42. ^{1}H NMR spectrum of $[PMePh_3]_2[Pt_{12}(CO)_{22}\{P(OMe)_3\}_2]$ in acetone-d_6 at 298 K.

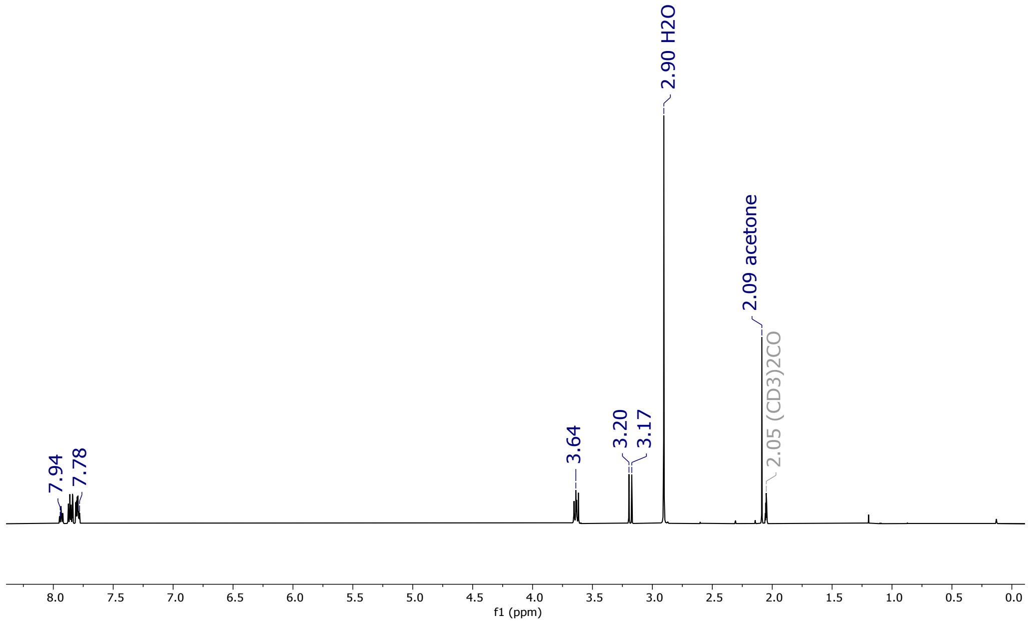

Figure S43. ^{1}H NMR spectrum of a mixture of $[PMePh_3]_2[Pt_9(CO)_{17}\{P(OMe)_3\}]$ and $[PMePh_3]_2[Pt_9(CO)_{16}\{P(OMe)_3\}_2]$ in acetone-d_6 at 298 K.

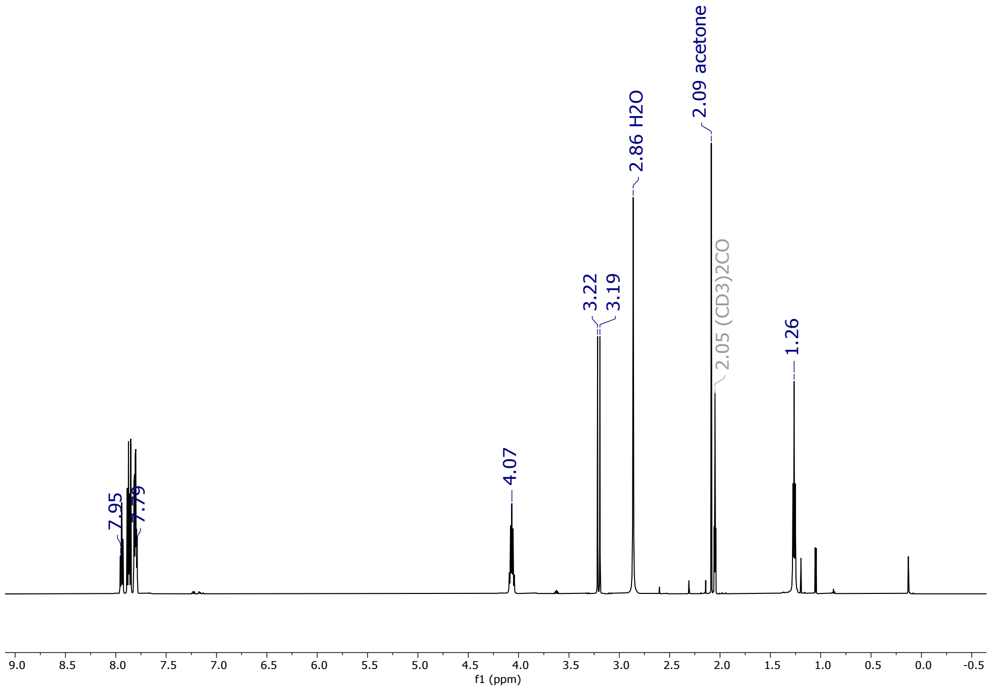

Figure S44. ^{1}H NMR spectrum of $[PMePh_3]_2[Pt_{12}(CO)_{22}\{P(OEt)_3\}_2]$ in acetone-d_6 at 298 K.

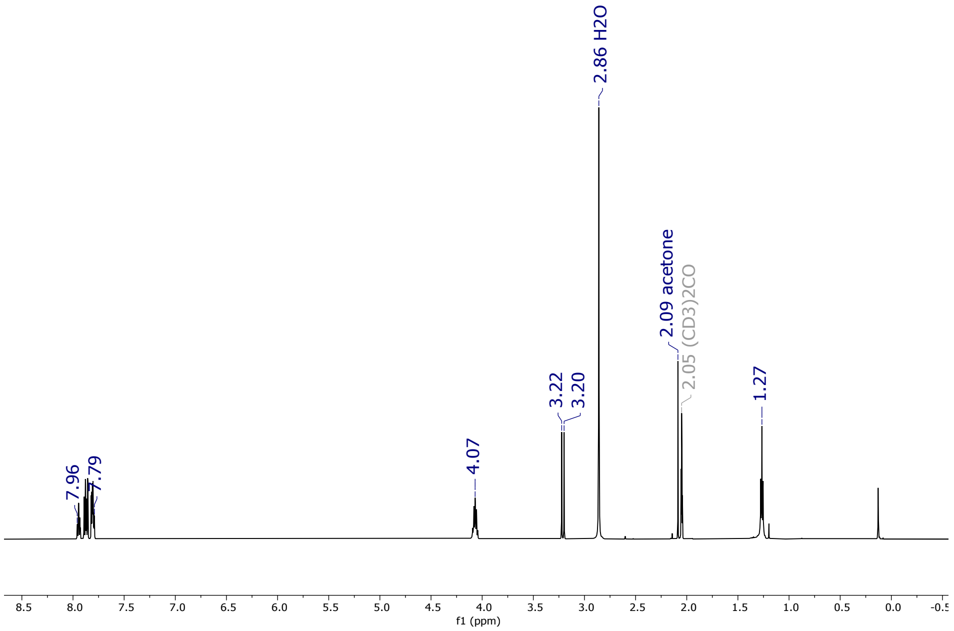

Figure S45. ^{1}H NMR spectrum of $[PMePh_3]_2[Pt_{12}(CO)_{22}\{P(OEt)_3\}_2]$ in acetone-d_6 at 298 K.

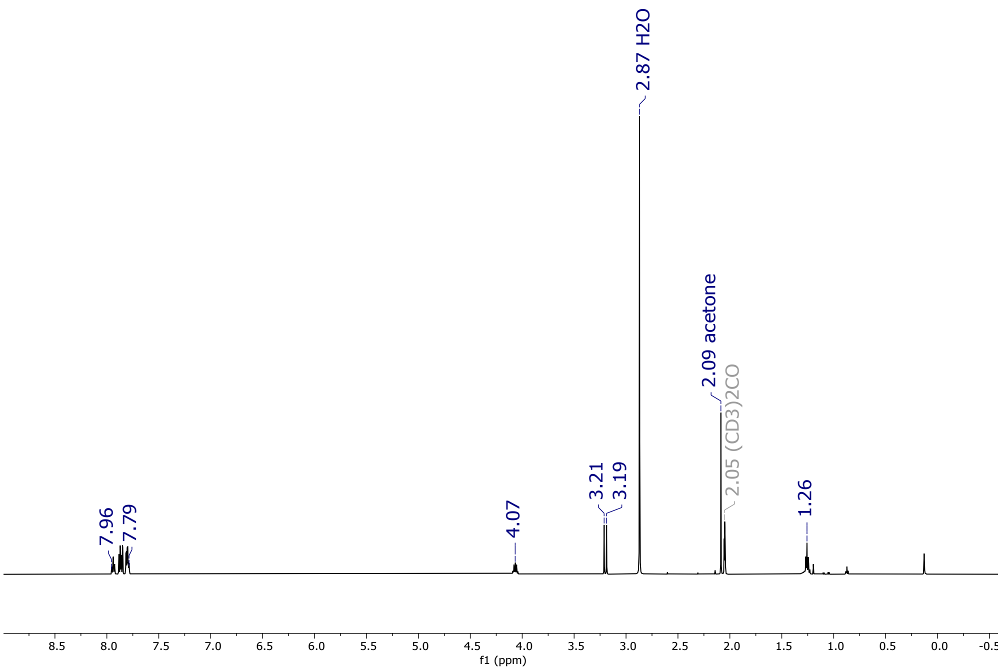

Figure S46. ^{1}H NMR spectrum of $[PMePh_3]_2[Pt_9(CO)_{17}\{P(OEt)_3\}]$ in acetone-d_6 at 298 K.

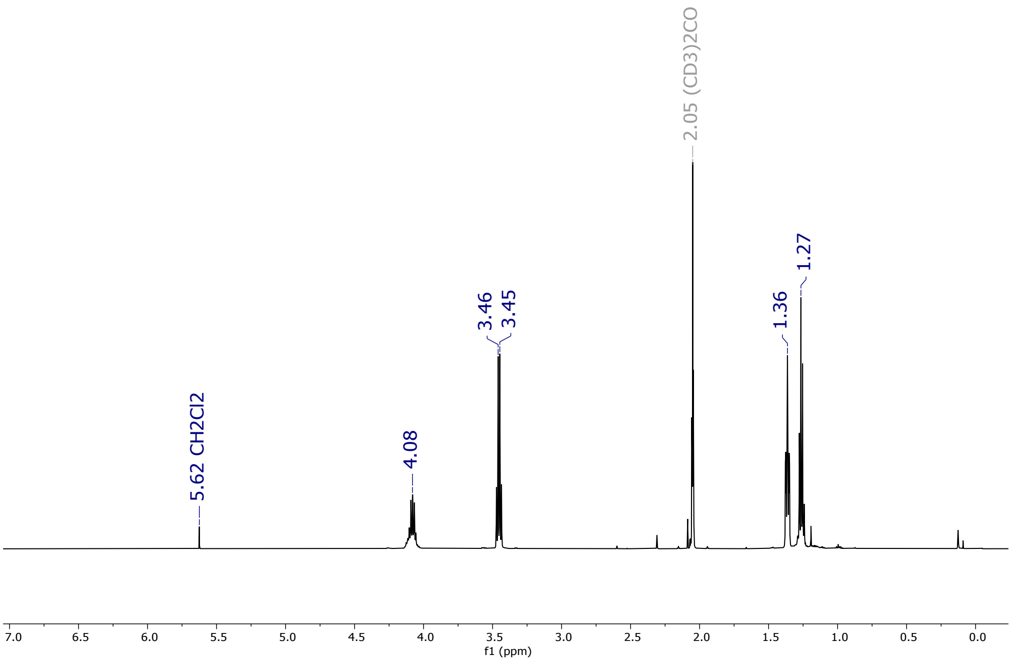

Figure S47. ^{1}H NMR spectrum of $[NEt_4]_2[Pt_9(CO)_{16}\{P(OEt)_3\}_2]$ in acetone-d_6 at 298 K.

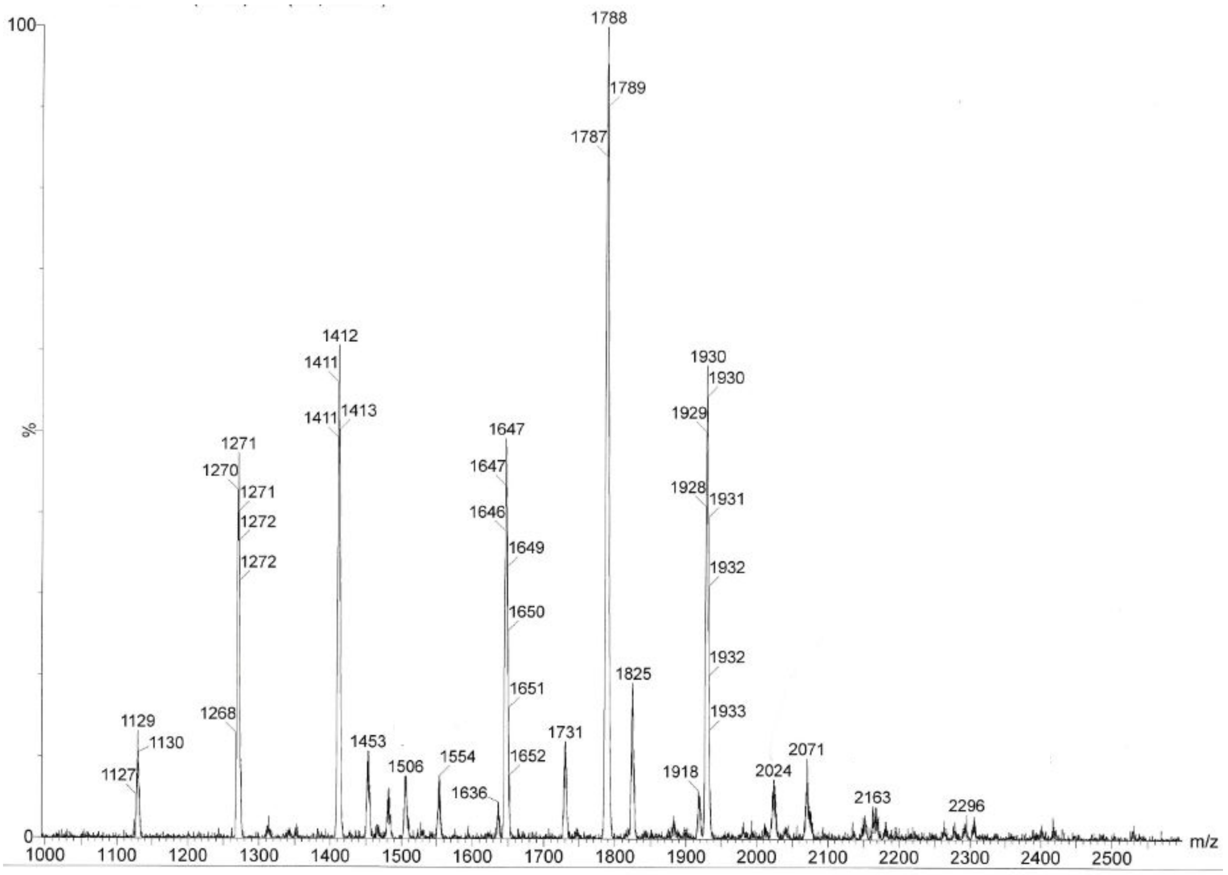

Figure S48. ESI-MS spectrum (relative intensity (%) vs m/z) in MeCN solution (ES-) of the crude of the reaction of $[Pt_9(CO)_{18}]^{2-}$ with two mole equivalents of $P(OPh)_3$.

Table S1. Peak assignment of the ESI-MS spectrum of $[Pt_9(CO)_{18}]^{2-}$ + 2 $P(OPh)_3$.

m/z	Relative intensity	Ion
1129	15	$[Pt_9(CO)_{18}]^{2-}$
1271	50	$[Pt_9(CO)_{17}\{P(OPh)_3\}]^{2-}$
1412	65	$[Pt_9(CO)_{16}\{P(OPh)_3\}_2]^{2-}$
1506	10	$[Pt_{12}(CO)_{24}]^{2-}$
1554	8	$[Pt_9(CO)_{15}\{P(OPh)_3\}_3]^{2-}$
1647	50	$[Pt_{12}(CO)_{23}\{P(OPh)_3\}]^{2-}$
1788	100	$[Pt_{12}(CO)_{22}\{P(OPh)_3\}_2]^{2-}$
1930	65	$[Pt_{12}(CO)_{21}\{P(OPh)_3\}_3]^{2-}$
2024	10	$[Pt_{15}(CO)_{29}\{P(OPh)_3\}]^{2-}$
2071	15	$[Pt_{12}(CO)_{20}\{P(OPh)_3\}_4]^{2-}$
2163	5	$[Pt_{15}(CO)_{28}\{P(OPh)_3\}_2]^{2-}$

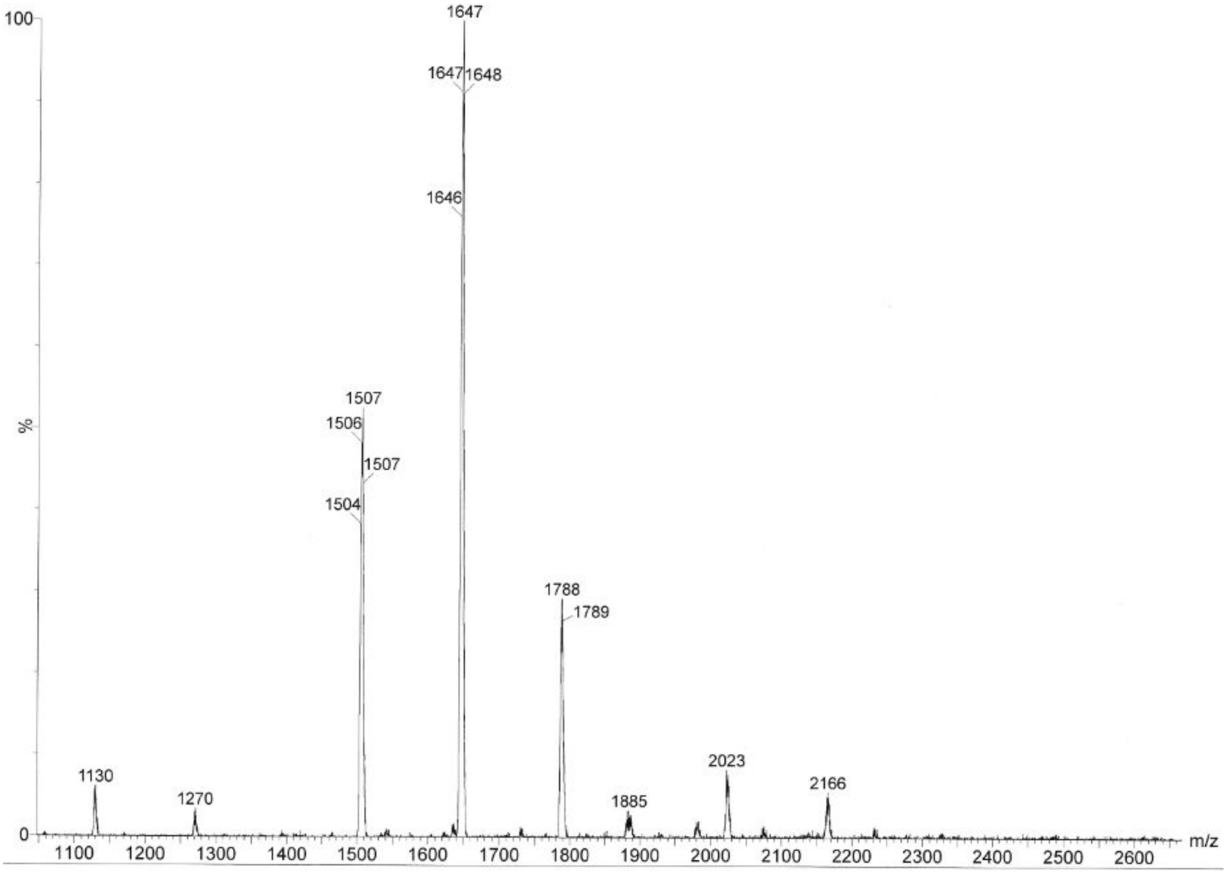

Figure S49. ESI-MS spectrum (relative intensity (%) vs m/z) in MeCN solution (ES-) of the crude of the reaction of $[Pt_{15}(CO)_{30}]^{2-}$ with two mole equivalents of $P(OPh)_3$.

Table S2. Peak assignment of the ESI-MS spectrum of $[Pt_{15}(CO)_{30}]^{2-}$ + 2 $P(OPh)_3$.

**m/z**	**Relative intensity**	**Ion**
1130	8	$[Pt_9(CO)_{18}]^{2-}$
1270	4	$[Pt_9(CO)_{17}\{P(OPh)_3\}]^{2-}$
1507	50	$[Pt_{12}(CO)_{24}]^{2-}$
1647	100	$[Pt_{12}(CO)_{23}\{P(OPh)_3\}]^{2-}$
1788	30	$[Pt_{12}(CO)_{22}\{P(OPh)_3\}_2]^{2-}$
1885	4	$[Pt_{15}(CO)_{30}]^{2-}$
1927	2	$[Pt_{12}(CO)_{21}\{P(OPh)_3\}_3]^{2-}$
2023	10	$[Pt_{15}(CO)_{29}\{P(OPh)_3\}]^{2-}$
2166	8	$[Pt_{15}(CO)_{28}\{P(OPh)_3\}_2]^{2-}$

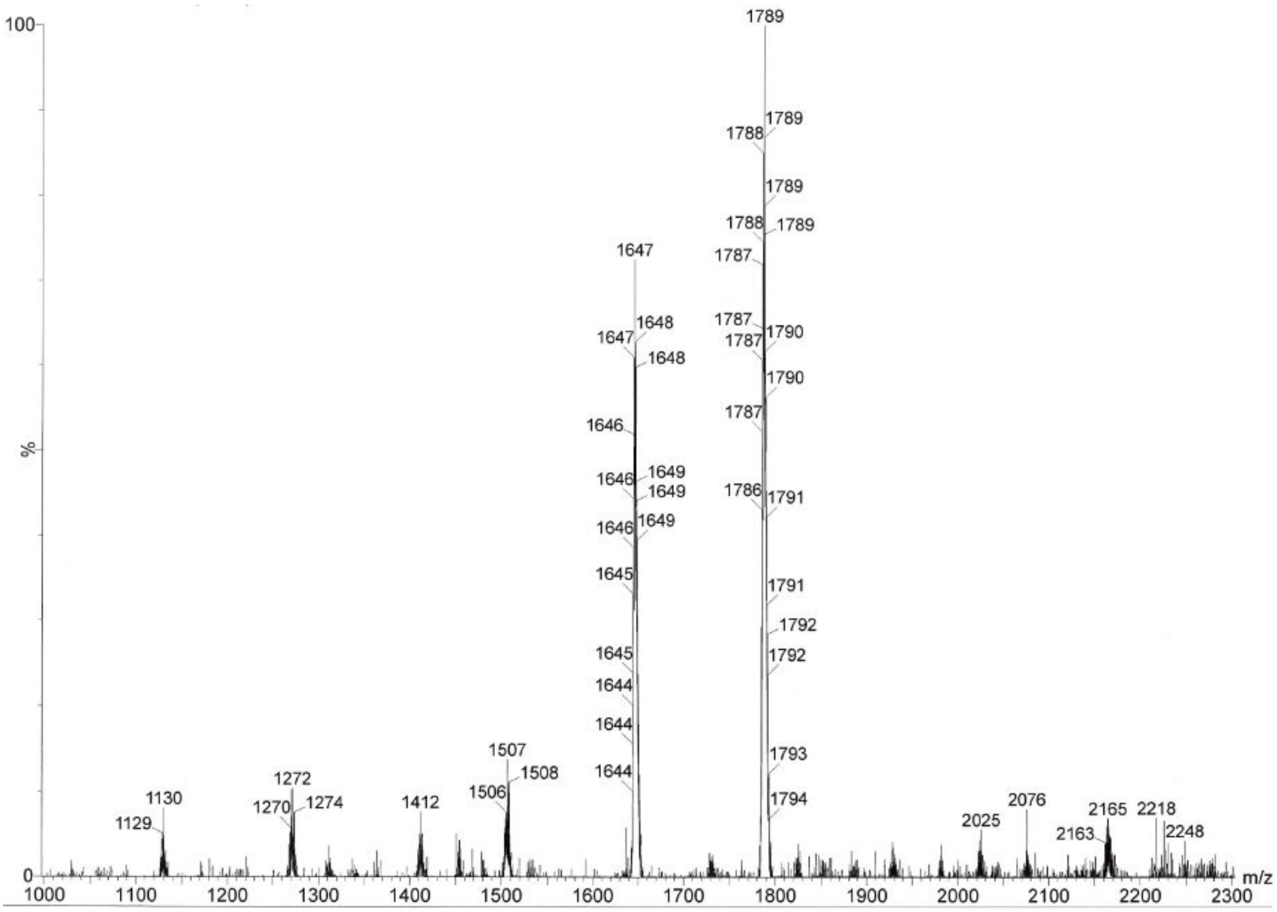

Figure S50. ESI-MS spectrum (relative intensity (%) vs m/z) in MeCN solution (ES-) of the crude of the reaction of $[Pt_{12}(CO)_{24}]^{2-}$ with two mole equivalents of $P(OPh)_3$.

Table S3. Peak assignment of the ESI-MS spectrum of $[Pt_{12}(CO)_{24}]^{2-}$ + 2 $P(OPh)_3$.

m/z	Relative intensity	Ion
1130	4	$[Pt_9(CO)_{18}]^{2-}$
1272	7	$[Pt_9(CO)_{17}\{P(OPh)_3\}]^{2-}$
1412	6	$[Pt_9(CO)_{16}\{P(OPh)_3\}_2]^{2-}$
1507	10	$[Pt_{12}(CO)_{24}]^{2-}$
1647	75	$[Pt_{12}(CO)_{23}\{P(OPh)_3\}]^{2-}$
1789	100	$[Pt_{12}(CO)_{22}\{P(OPh)_3\}_2]^{2-}$
2025	4	$[Pt_{15}(CO)_{29}\{P(OPh)_3\}]^{2-}$
2165	6	$[Pt_{15}(CO)_{28}\{P(OPh)_3\}_2]^{2-}$

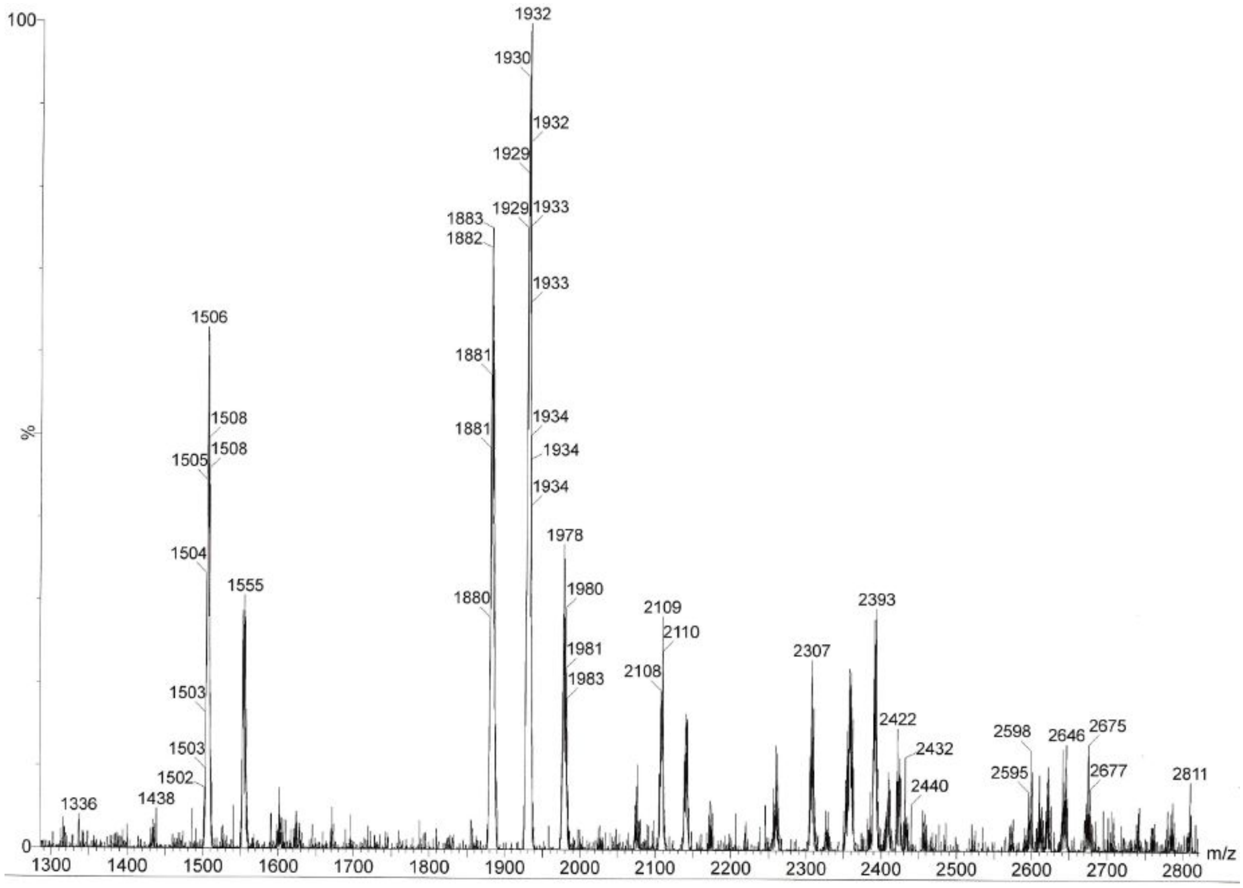

Figure S51. ESI-MS spectrum (relative intensity (%) vs m/z) in MeCN solution (ES-) of the crude of the reaction of $[Pt_{12}(CO)_{22}\{P(OMe)_3\}_2]^{2-}$ with 0.5 mole equivalents of $HBF_4 \cdot Et_2O$.

Table S4. Peak assignment of the ESI-MS spectrum of $[Pt_{12}(CO)_{22}\{P(OMe)_3\}_2]^{2-}$ + 0.5 $HBF_4 \cdot Et_2O$.

m/z	Relative intensity	Ion
1506	65	$[Pt_{12}(CO)_{24}]^{2-}$
1555	30	$[Pt_{12}(CO)_{23}\{P(OMe)_3\}]^{2-}$
1883	75	$[Pt_{15}(CO)_{30}]^{2-}$
1932	100	$[Pt_{15}(CO)_{29}\{P(OMe)_3\}]^{2-}$
1978	40	$[Pt_{15}(CO)_{28}\{P(OMe)_3\}_2]^{2-}$
2307	25	$[Pt_{18}(CO)_{35}\{P(OMe)_3\}_2]^{2-}$

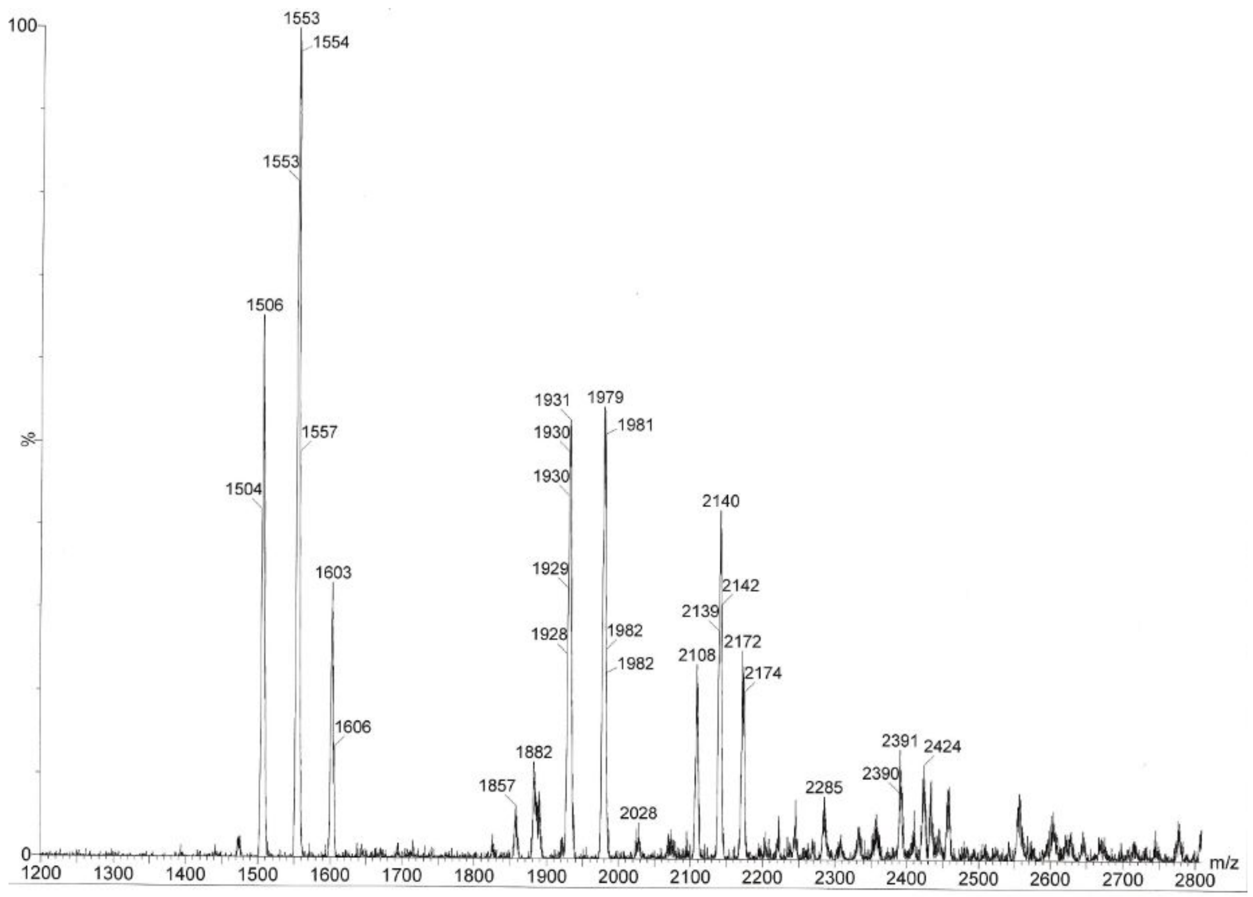

Figure S52. ESI-MS spectrum (relative intensity (%) vs m/z) in MeCN solution (ES-) of the crude of the reaction of $[Pt_9(CO)_{17}\{P(OMe)_3\}]^{2-}$ with one mole equivalent of $HBF_4{\cdot}Et_2O$.

Table S5. Peak assignment of the ESI-MS spectrum of $[Pt_9(CO)_{17}\{P(OMe)_3\}]^{2-}$ + 1 $HBF_4{\cdot}Et_2O$.

m/z	Relative intensity	Ion
1506	65	$[Pt_{12}(CO)_{24}]^{2-}$
1553	100	$[Pt_{12}(CO)_{23}\{P(OMe)_3\}]^{2-}$
1603	35	$[Pt_{12}(CO)_{22}\{P(OMe)_3\}_2]^{2-}$
1882	10	$[Pt_{15}(CO)_{30}]^{2-}$
1931	50	$[Pt_{15}(CO)_{29}\{P(OMe)_3\}]^{2-}$
1979	50	$[Pt_{15}(CO)_{28}\{P(OMe)_3\}_2]^{2-}$
2028	5	$[Pt_{15}(CO)_{27}\{P(OMe)_3\}_3]^{2-}$

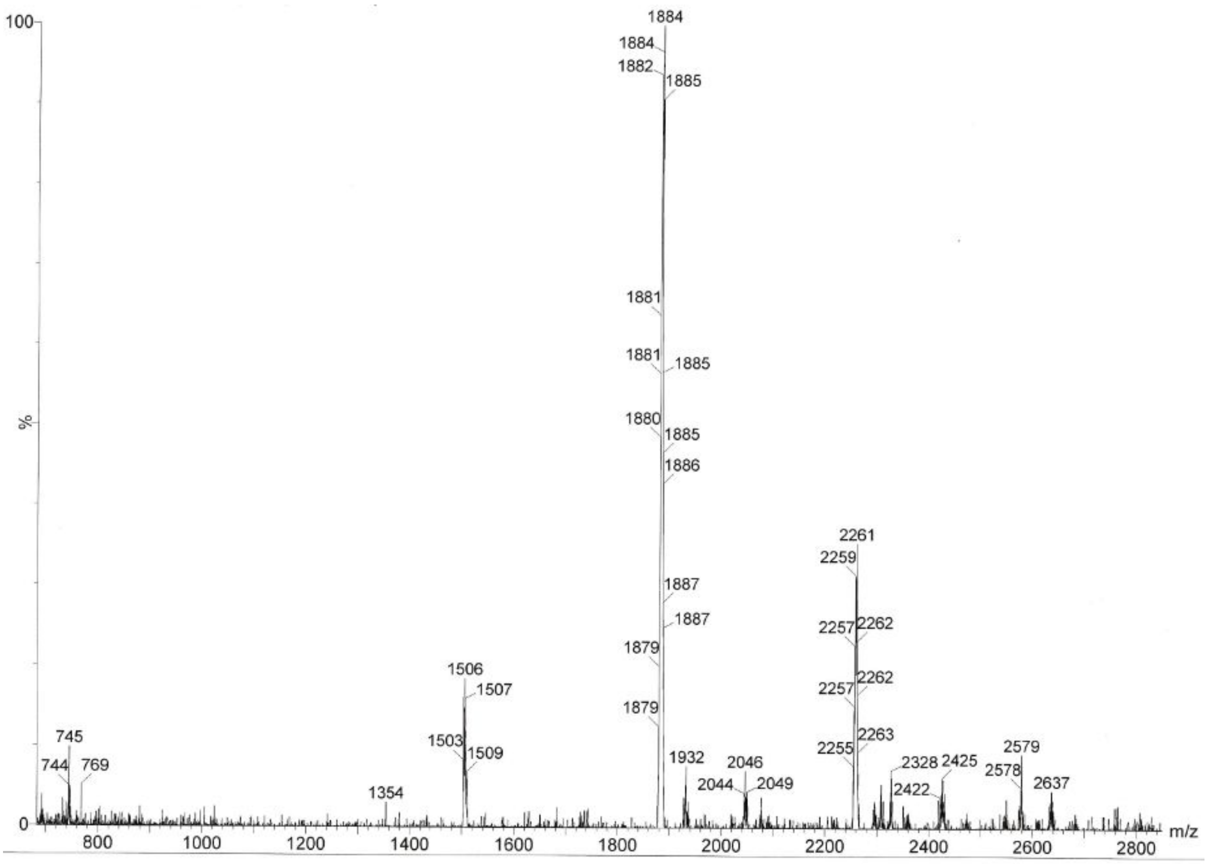

Figure S53. ESI-MS spectrum (relative intensity (%) vs m/z) in MeCN solution (ES-) of the crude of the reaction of $[Pt_9(CO)_{18}]^{2-}$ with two mole equivalents of $HBF_4{\cdot}Et_2O$.

Table S6. Peak assignment of the ESI-MS spectrum of $[Pt_9(CO)_{18}]^{2-}$ + 2 $HBF_4{\cdot}Et_2O$.

m/z	Relative intensity	Ion
1506	20	$[Pt_{12}(CO)_{24}]^{2-}$
1884	100	$[Pt_{15}(CO)_{30}]^{2-}$
2261	35	$[Pt_{18}(CO)_{36}]^{2-}$

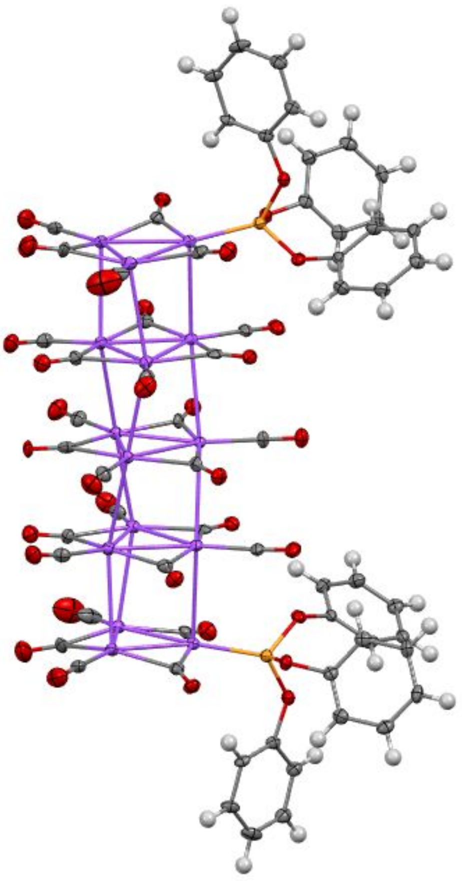

Figure S54. Molecular structure of $[Pt_{15}(CO)_{28}\{P(OPh)_3\}_2]^{2-}$ (purple, Pt; orange, P; red, O; grey, C; white, H). Thermal ellipsoids are at the 30% probability level.

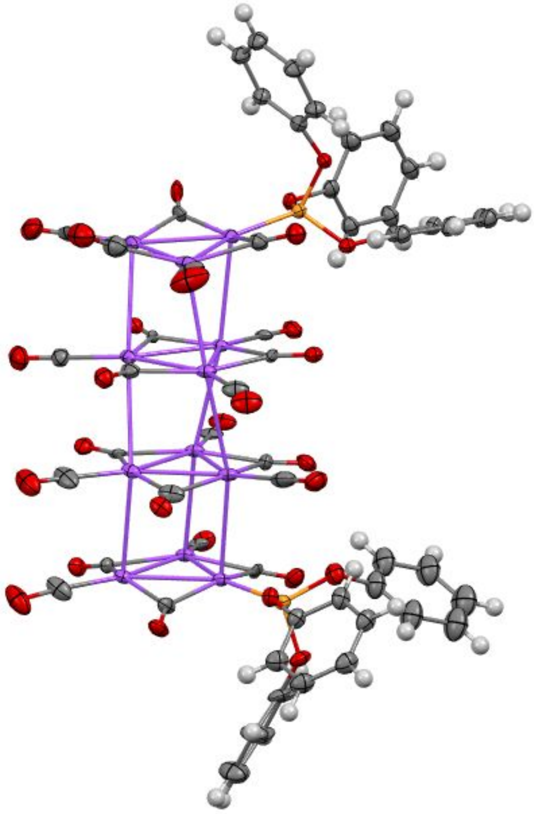

Figure S55. Molecular structure of $[Pt_{12}(CO)_{22}\{P(OPh)_3\}_2]^{2-}$ (purple, Pt; orange, P; red, O; grey, C; white, H). Thermal ellipsoids are at the 30% probability level.

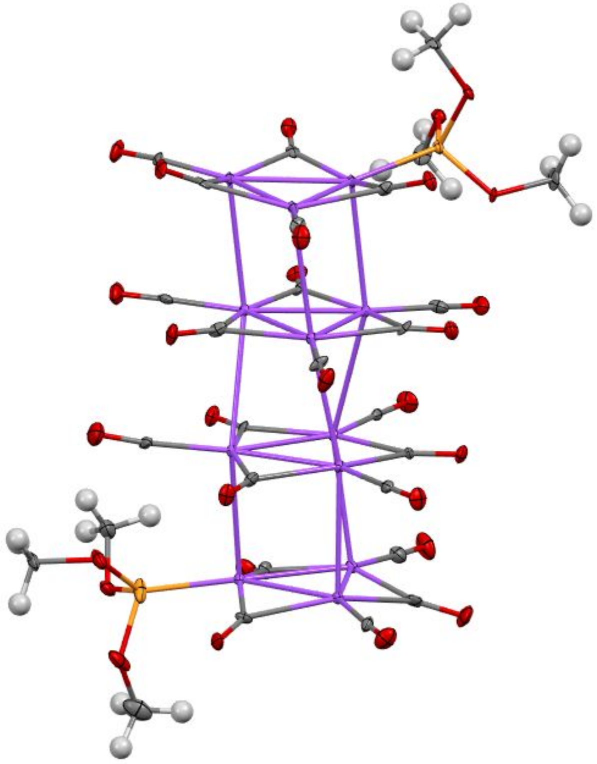

Figure S56. Molecular structure of $[Pt_{12}(CO)_{22}\{P(OMe)_3\}_2]^{2-}$ (purple, Pt; orange, P; red, O; grey, C; white, H). Thermal ellipsoids are at the 30% probability level.

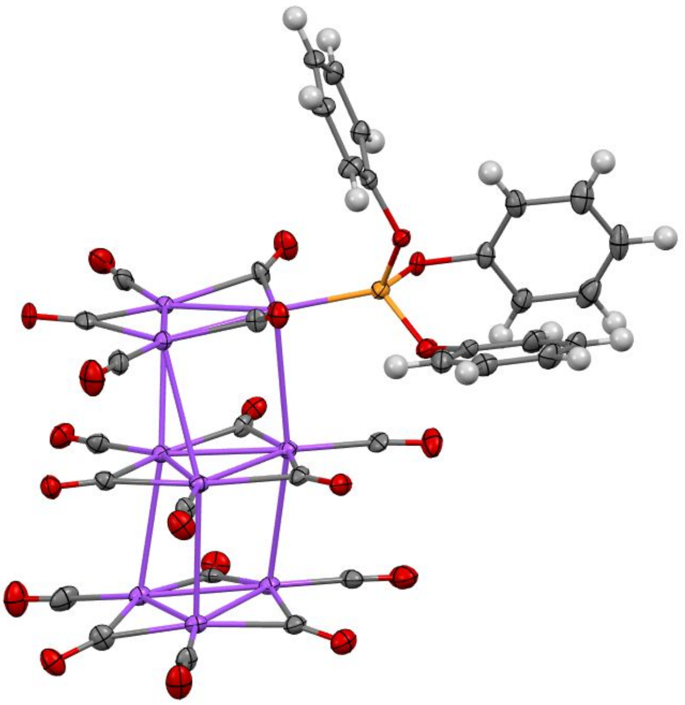

Figure S57. Molecular structure of $[Pt_9(CO)_{17}\{P(OPh)_3\}]^{2-}$ (purple, Pt; orange, P; red, O; grey, C; white, H). Thermal ellipsoids are at the 30% probability level.

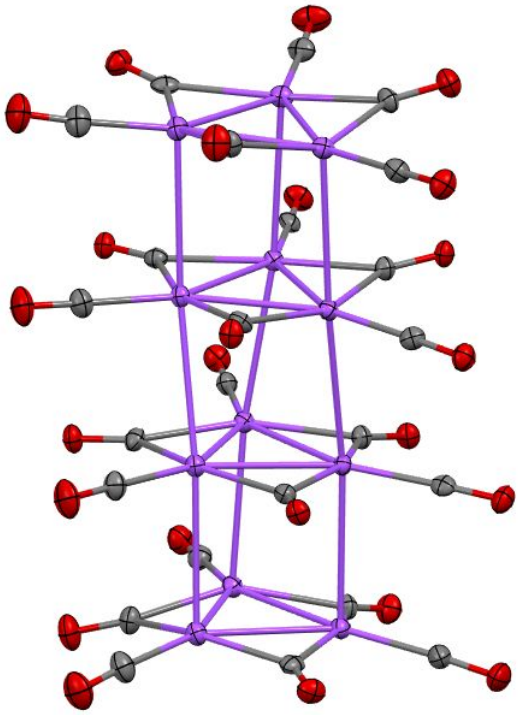

Figure S58. Molecular structure of $[Pt_{12}(CO)_{24}]^{2-}$ (purple, Pt; red, O; grey, C). Thermal ellipsoids are at the 30% probability level.

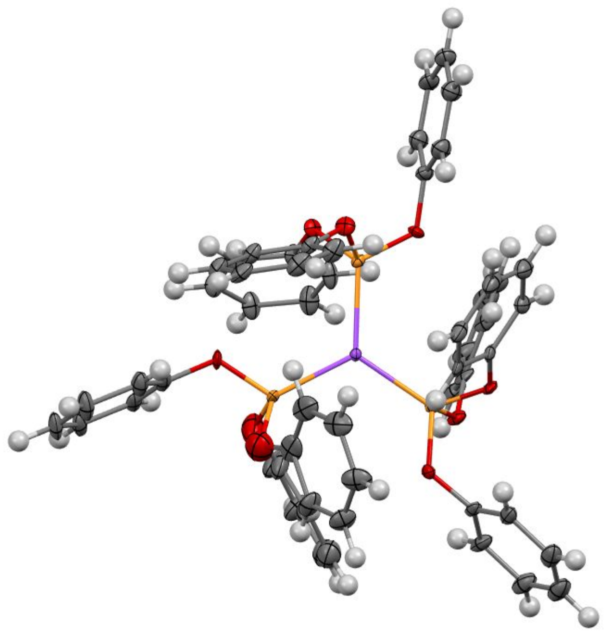

Figure S59. Molecular structure of [Pt{P(OPh)$_3$}$_3$] (purple, Pt; orange, P; red, O; grey, C; white, H). Thermal ellipsoids are at the 30% probability level.

X-ray Crystallographic Study.

Crystal data and collection details for $[PMePh_3]_2[Pt_9(CO)_{17}\{P(OPh)_3\}]\cdot CH_3COCH_3$, $[PMePh_3]_2[Pt_{12}(CO)_{22}\{P(OPh)_3\}_2]\cdot$solv, $[PMePh_3]_2[Pt_{12}(CO)_{22}\{P(OMe)_3\}_2]$, $[PMePh_3]_2[Pt_{15}(CO)_{28}\{P(OPh)_3\}_2]\cdot 2CH_3COCH_3\cdot C_6H_{14}$, $[PMePh_3]_2[Pt_{12}(CO)_{24}]$ and $[Pt\{P(OPh)_3\}_3]\cdot$THF are reported in Table S7. The diffraction experiments were carried out on a Bruker APEX II diffractometer equipped with a PHOTON2 detector using Mo–Kα radiation. Data were corrected for Lorentz polarization and absorption effects (empirical absorption correction SADABS).[1] Structures were solved by direct methods and refined by full-matrix least-squares based on all data using F^2.[2] Hydrogen atoms were fixed at calculated positions and refined by a riding model. All non-hydrogen atoms were refined with anisotropic displacement parameters, unless otherwise stated.

$[PMePh_3]_2[Pt_9(CO)_{17}\{P(OPh)_3\}]\cdot CH_3COCH_3$: The asymmetric unit of the unit cell contains one cluster anion, two $[PMePh_3]^+$ cations and one CH_3COCH_3 molecule, all located on general positions. The external Pt_3 triangle (not bonded to the phosphite ligand) is slightly disordered and it has been split into two positions.

$[PMePh_3]_2[Pt_{12}(CO)_{22}\{P(OPh)_3\}_2]\cdot$solv: The asymmetric unit of the unit cell contains one cluster anion and one $[PMePh_3]^+$ cation located on general positions, and two halves of one $[PMePh_3]^+$ cation disordered over two symmetry related (by an inversion center) positions. Also, one $Pt_3(CO)_6$ unit of the cluster anion is disordered and has been split into two positions. Because of the disorder, several restraints have been employed during the refinement. The unit cell contains an additional total potential solvent accessible void of 549 Å^3 (*ca.* 10 % of the Cell Volume) which is likely to be occupied by highly disordered solvent molecules. These voids have been treated using the SQUEEZE routine of PLATON.[3,4]

$[PMePh_3]_2[Pt_{12}(CO)_{22}\{P(OMe)_3\}_2]$: The asymmetric unit of the unit cell contains one cluster anion and two $[PMePh_3]^+$ cations, all located on general positions. The two external Pt_3 triangles and one $P(OMe)_3$ ligand are disordered and have been split each into two positions. Some restraints have been employed to model the disorder.

$[PMePh_3]_2[Pt_{15}(CO)_{28}\{P(OPh)_3\}_2]\cdot 2CH_3COCH_3\cdot C_6H_{14}$: The asymmetric unit of the unit cell contains half of one cluster anion (located on a 2-fold axis), one $[PMePh_3]^+$ cation (located on a general position), one CH_3COCH_3 molecule (located on a general position), and half of a C_6H_{14} molecule disordered over two symmetry related (by a 2-fold axis) positions. The external Pt_3 triangle is slightly disordered and it has been split into two positions.

$[PMePh_3]_2[Pt_{12}(CO)_{24}]$: The asymmetric unit of the unit cell contains half of one cluster anion (located on a 2-fold axis) and one $[PMePh_3]^+$ cation (located on a general position).

[Pt{P(OPh)$_3$}$_3$]·THF: The asymmetric unit of the unit cell contains two Pt complexes and two THF molecules, all located on general positions. The crystals are racemically twinned with refined batch factor 0.341(12).

Table S7. Crystal data and experimental details [PMePh$_3$]$_2$[Pt$_9$(CO)$_{17}${P(OPh)$_3$}]·CH$_3$COCH$_3$, [PMePh$_3$]$_2$[Pt$_{12}$(CO)$_{22}${P(OPh)$_3$}$_2$], [PMePh$_3$]$_2$[Pt$_{12}$(CO)$_{22}${P(OMe)$_3$}$_2$], [PMePh$_3$]$_2$[Pt$_{15}$(CO)$_{28}${P(OPh)$_3$}$_2$]·2CH$_3$COCH$_3$·C$_6$H$_{14}$, [PMePh$_3$]$_2$[Pt$_{12}$(CO)$_{24}$] and [Pt{P(OPh)$_3$}$_3$]·THF.

	**[PMePh$_3$]$_2$[Pt$_9$(CO)$_{17}${P(OPh)$_3$}]·CH$_3$COCH$_3$**	**[PMePh$_3$]$_2$ [Pt$_{12}$(CO)$_{22}${P(OPh)$_3$}$_2$]·solv**	**[PMePh$_3$]$_2$ [Pt$_{12}$(CO)$_{22}${P(OMe)$_3$}$_2$]**
Formula	C$_{76}$H$_{57}$O$_{21}$P$_3$Pt$_9$	C$_{96}$H$_{66}$O$_{28}$P$_4$Pt$_{12}$	C$_{66}$H$_{54}$O$_{28}$P$_4$Pt$_{12}$
Fw	3154.93	4132.44	3760.04
T, K	100(2)	100(2)	100(2)
λ, Å	0.71073	0.71073	0.71073
Crystal system	Monoclinic	Triclinic	Triclinic
Space Group	*P*2$_1$/n	$P\bar{1}$	$P\bar{1}$
a, Å	14.7156(5)	15.8789(8)	11.5342(5)
b, Å	23.9491(9)	18.1353(10)	14.4687(6)
c, Å	23.4817(9)	19.8642(10)	24.6706(10)
α, °	90	96.299(2)	99.7420(10)
β, °	107.3040(10)	102.304(2)	96.3820(10)
γ, °	90	101.498(2)	94.0600(10)
Cell Volume, Å^3	7901.0(5)	5407.1(5)	4015.7(3)
Z	4	2	2
D$_c$, g cm^{-3}	2.652	2.538	3.110
μ, mm^{-1}	15.999	15.583	20.965
F(000)	5712	3724	3340
Crystal size, mm	0.16×0.12×0.09	0.21×0.16×0.11	0.18×0.16×0.12
θ limits, °	1.680–25.099	2.478–25.499	1.529–25.198
Index ranges	-17 ≤ h ≤ 17<br>-28 ≤ k ≤ 28<br>-28 ≤ l ≤ 28	-18 ≤ h ≤ 18<br>-21 ≤ k ≤ 21<br>-23 ≤ l ≤ 23	-13 ≤ h ≤ 13<br>-17 ≤ k ≤ 17<br>-29 ≤ l ≤ 29
Reflections collected	74198	64921	53484

Independent reflections	14076 [R_{int} = 0.0656]	19126 [R_{int} = 0.0602]	14435 [R_{int} = 0.0661]
Completeness to θ max	100.0%	99.8%	99.7%
Data / restraints / parameters	14076 / 6 / 992	19126 / 849 / 1094	14435 / 228 / 1042
Goodness on fit on F^2	1.065	1.163	1.110
R_1 ($I > 2\sigma(I)$)	0.0374	0.0754	0.0390
wR_2 (all data)	0.0833	0.1719	0.0980
Largest diff. peak and hole, e Å^{-3}	2.296 / –1.472	9.380 / –2.638	1.864 / –2.377

	**$[PMePh_3]_2[Pt_{15}(CO)_{28}\{P(OPh)_3\}_2]\cdot 2CH_3COCH_3\cdot C_6H_{14}$**	**$[PMePh_3]_2[Pt_{12}(CO)_{24}]$**	**$[Pt\{P(OPh)_3\}_3]\cdot THF$**
Formula	$C_{114}H_{92}O_{36}P_4Pt_{15}$	$C_{62}H_{36}O_{24}Pt_{12}$	$C_{58}H_{53}O_{10}P_3Pt$
Fw	5088.10	3567.93	1198.00
T, K	100(2)	100(2)	100(2)
λ, Å	0.71073	0.71073	0.71073
Crystal system	Orthorhombic	Monoclinic	Orthorhombic
Space Group	*Ibca*	*C*2/c	*Pna*2_1
a, Å	18.8393(5)	30.3086(9)	19.4550(9)
b, Å	31.1043(8)	16.3915(5)	12.1884(6)
c, Å	42.2195(11)	15.0508(5)	43.3006(18)
α, °	90	90	90
β, °	90	110.9490(10)	90
γ, °	90	90	90
Cell Volume, Å^3	24739.9(11)	6983.0(4)	10267.7(8)
Z	8	4	8
D_c, g cm^{-3}	2.732	3.394	1.550
μ, mm^{-1}	17.015	24.055	2.887
F(000)	18352	6264	4832
Crystal size, mm	0.16×0.13×0.09	0.16×0.12×0.09	0.14×0.11×0.07
θ limits, °	1.590–25.200	1.848–25.999	1.736–25.050

Index ranges	$-22 \leq h \leq 22$<br>$-37 \leq k \leq 37$<br>$-50 \leq l \leq 50$	$-37 \leq h \leq 37$<br>$-20 \leq k \leq 20$<br>$-18 \leq l \leq 18$	$-23 \leq h \leq 23$<br>$-14 \leq k \leq 14$<br>$-51 \leq l \leq 51$
Reflections collected	117003	36717	94463
Independent reflections	11163 [R_{int} = 0.0754]	6870 [R_{int} = 0.0560]	18192 [R_{int} = 0.0852]
Completeness to θ max	100.0%	100.0%	100.0%
Data / restraints / parameters	11163 / 21 / 774	6870 / 0 / 452	18192 / 848 / 1032
Goodness on fit on F^2	1.139	1.057	1.099
R_1 ($I > 2\sigma(I)$)	0.0390	0.0304	0.0670
wR_2 (all data)	0.0915	0.0759	0.1480
Largest diff. peak and hole, e Å^{-3}	2.071 / –1.594	1.931 / –1.836	2.899 / –3.520

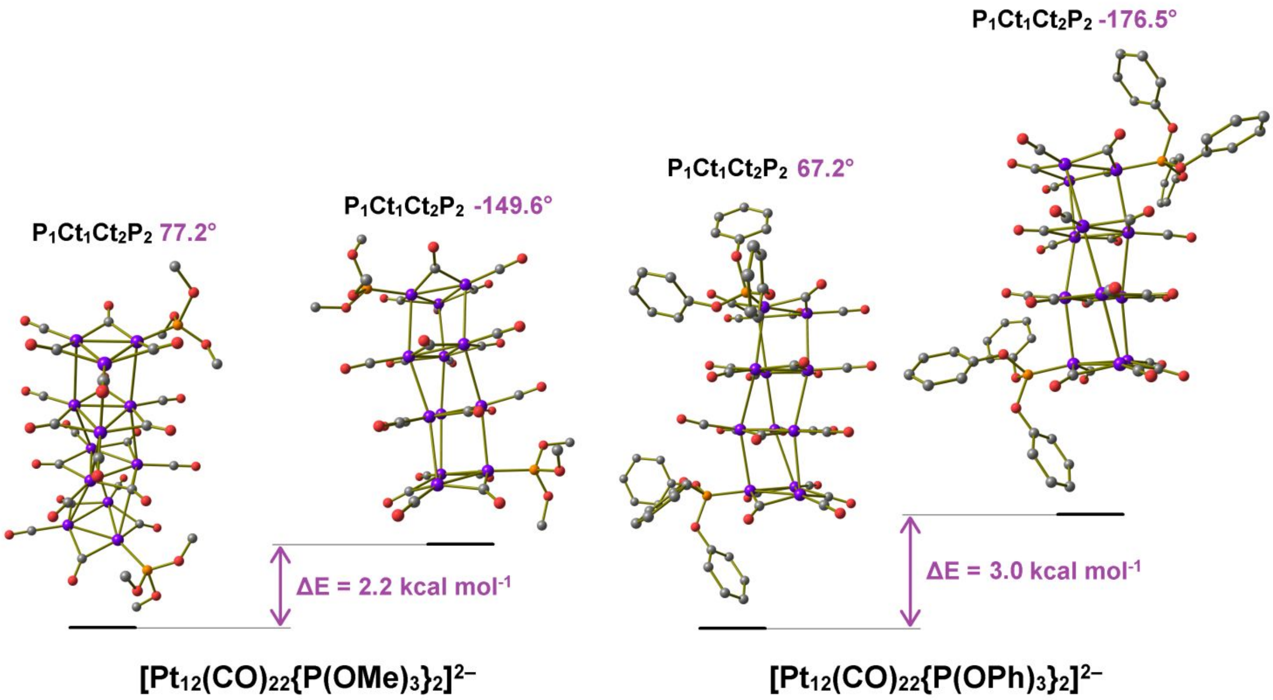

Figure S60. DFT-optimized structures and relative energy differences of the torsional isomers of $[Pt_{12}(CO)_{22}\{P(OR)_3\}_2]^{2-}$ (R = Me, Ph).

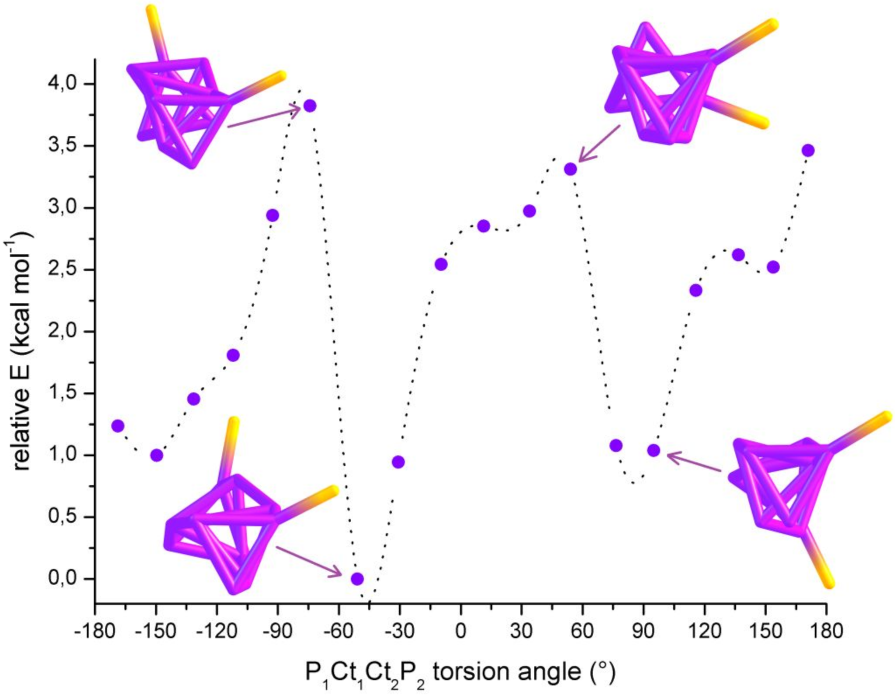

Figure S61. Relative energy values of the DFT-optimized torsional isomers of $[Pt_{12}(CO)_{22}\{P(OMe)_3\}_2]^{2-}$ obtained during relaxed scan calculations. The points are connected with a dotted spline for clarity purposes. Selected DFT-optimized structures are shown (purple, Pt; orange, P; other atoms are omitted). All the DFT-optimized structures are merged in an animated .gif file provided as Supporting Material.

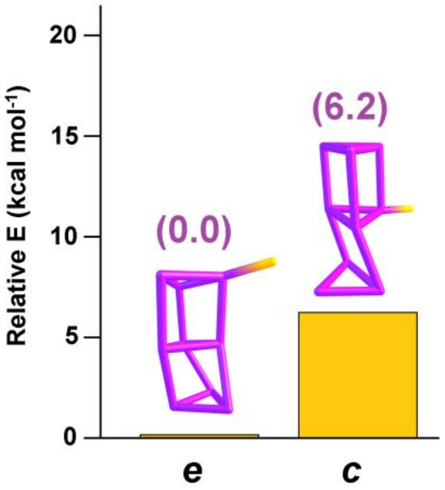

Figure S62. DFT-optimized structures (PBEh-3c method) of the positional isomers of $[Pt_9(CO)_{17}\{P(OMe)_3\}]^{2-}$ with relative energy values (kcal mol^{-1}). Purple, Pt; orange, P; other atoms omitted. Energy difference at TPSS0/def2-TVZP level equal to 7.9 kcal mol^{-1}.

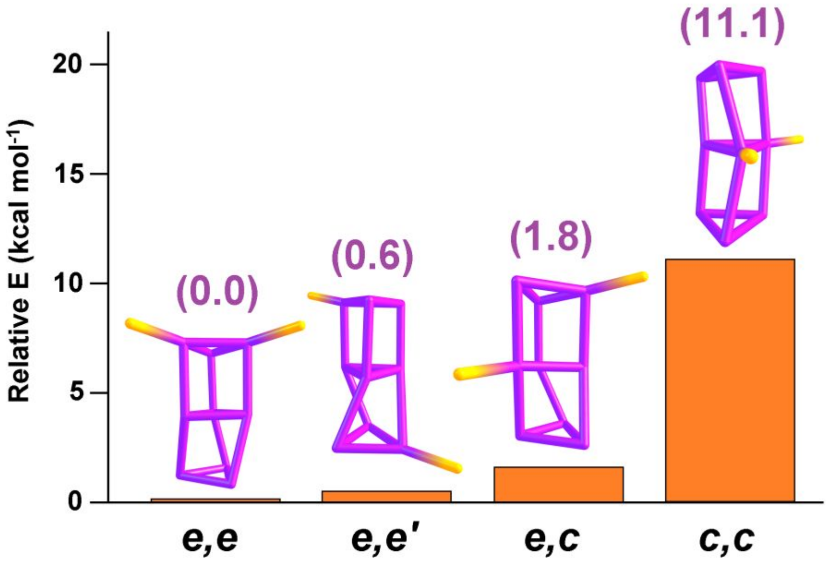

Figure S63. DFT-optimized structures (PBEh-3c method) of the positional isomers of $[Pt_9(CO)_{16}\{P(OMe)_3\}_2]^{2-}$ (staggered configurations) with relative energy values (kcal mol^{-1}). Purple, Pt; orange, P; other atoms omitted.

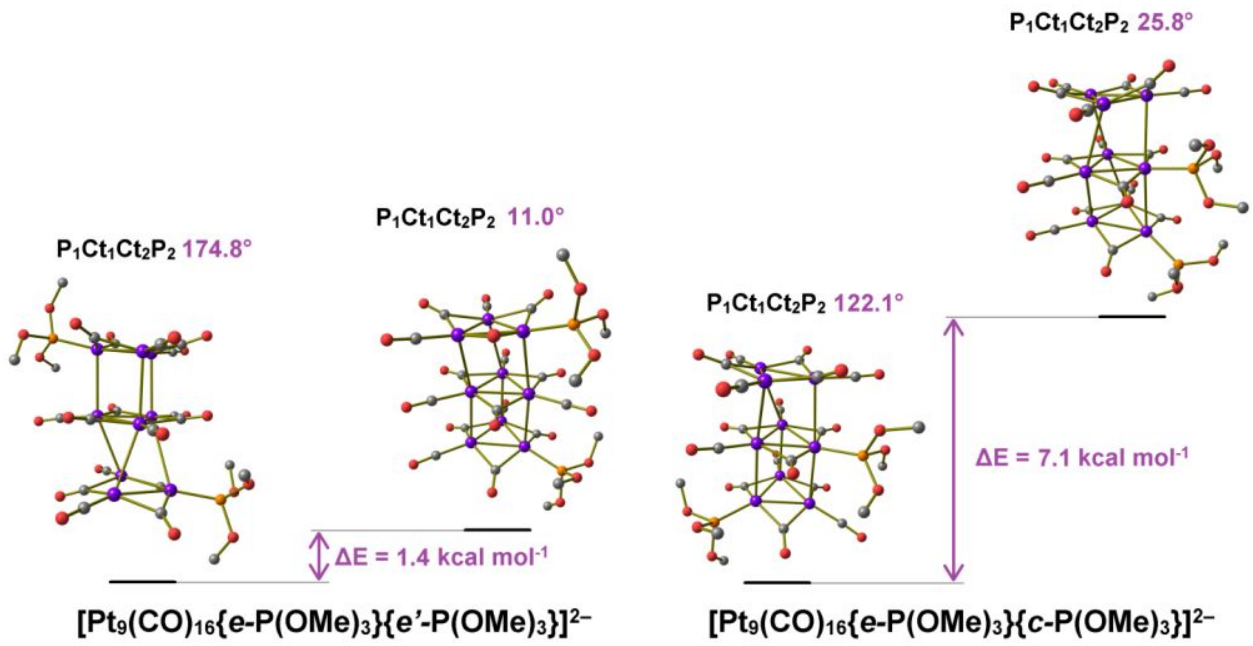

Figure 64. DFT-optimized structures (PBEh-3c method) and relative energy differences of the torsional isomers of $[Pt_9(CO)_{16}\{e\text{-}P(OMe)_3\}\{e'\text{-}P(OMe)_3\}]^{2-}$ and $[Pt_9(CO)_{16}\{e\text{-}P(OMe)_3\}\{c\text{-}P(OMe)_3\}]^{2-}$. Purple, Pt; orange, P; red, O; C, grey. Hydrogen atoms are omitted.

Table S8. Relative energy values (kcal mol^{-1}) of the isomers of $[Pt_9(CO)_{16}\{e\text{-}P(OMe)_3\}_2]^{2-}$.

Cluster	PBEh-3c	C-PCM/PBEh-3c	TPSS0/def2-TZVP
$[Pt_9(CO)_{16}\{e\text{-}P(OMe)_3\}_2]^{2-}$	0.0	0.3	0.0
$[Pt_9(CO)_{16}\{e\text{-}P(OMe)_3\}\{e'\text{-}P(OMe)_3\}]^{2-}$ (staggered)	0.6	0.0	0.2
$[Pt_9(CO)_{16}\{e\text{-}P(OMe)_3\}\{e'\text{-}P(OMe)_3\}]^{2-}$ (eclipsed)	2.0	1.7	0.8
$[Pt_9(CO)_{16}\{e\text{-}P(OMe)_3\}\{c\text{-}P(OMe)_3\}]^{2-}$ (staggered)	1.8		3.4
$[Pt_9(CO)_{16}\{e\text{-}P(OMe)_3\}\{c\text{-}P(OMe)_3\}]^{2-}$ (eclipsed)	8.8		10.8
$[Pt_9(CO)_{16}\{c\text{-}P(OMe)_3\}_2]^{2-}$	11.1		12.9

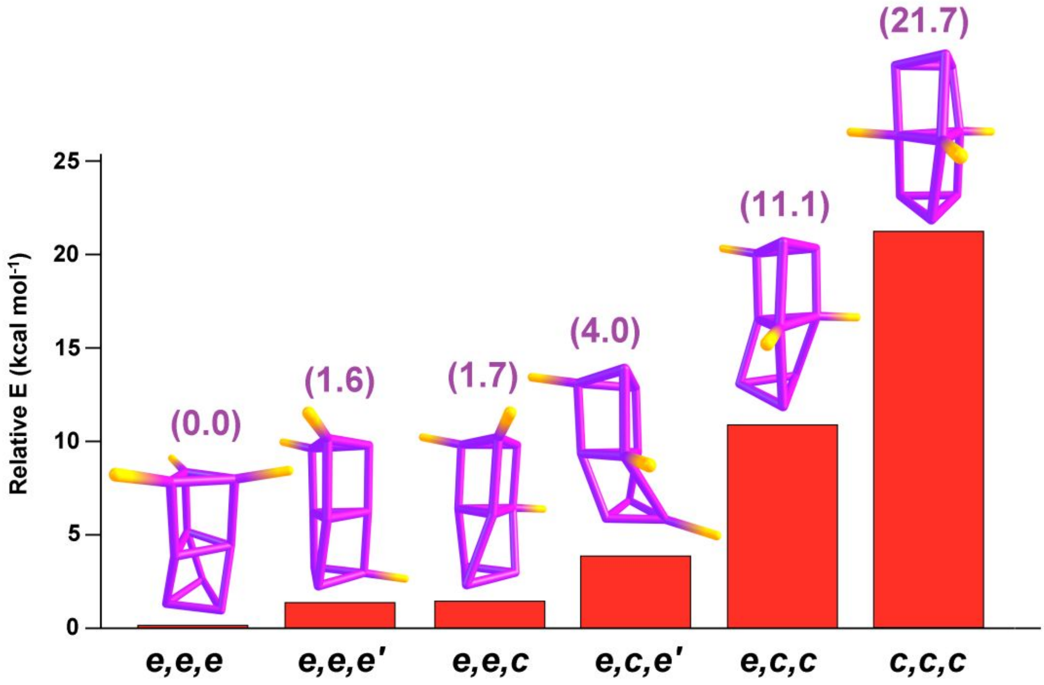

Figure S65. DFT-optimized structures (PBEh-3c method) of the positional isomers of $[Pt_9(CO)_{15}\{P(OMe)_3\}_3]^{2-}$ (staggered configurations) with relative energy values (kcal mol^{-1}). Purple, Pt; orange, P; other atoms omitted.

REFERENCES

(1) Sheldrick, G. M. *SADABS-2008/1-Bruker AXS Area Detector Scaling and Absorption Correction*; Bruker AXS: Madison, WI, 2008.

(2) Sheldrick, G. M. Crystal Structure Refinement with SCHELXL. *Acta Crystallogr., Sect. C: Struct. Chem.* **2015**, *71*, 3-8.

(3) Spek, A. L. Single-crystal structure validation with the program *PLATON*. *J. Appl. Crystallogr.* **2003**, *36*, 7-13.

(4) Spek, A. L. Structure validation in chemical crystallography. *Acta Crystallogr.* **2009**, *D65*, 148-155.
